# Discovery of Novel Dihydrolipoamide S-Succinyltransferase Inhibitors Based on Fragment Virtual Screening

**DOI:** 10.3390/ijms222312953

**Published:** 2021-11-30

**Authors:** Chengqian Wei, Junjie Huang, Yu Wang, Yifang Chen, Xin Luo, Shaobo Wang, Zengxue Wu, Jixiang Chen

**Affiliations:** State Key Laboratory Breeding Base of Green Pesticide and Agricultural Bioengineering, Key Laboratory of Green Pesticide and Agricultural Bioengineering, Ministry of Education, Center for Research and Development of Fine Chemicals, Guizhou University, Huaxi District, Guiyang 550025, China; wcq1996@163.com (C.W.); hjj18744902440@163.com (J.H.); gs.wy20@gzu.edu.cn (Y.W.); gs.chenyf20@gzu.edu.cn (Y.C.); gs.xunluo20@gzu.edu.cn (X.L.); wangshaobo97@163.com (S.W.); wuzx@gzu.edu.cn (Z.W.)

**Keywords:** antibacterial activity, fragment, virtual screening, DLST inhibitors

## Abstract

A series of new oxadiazole sulfone derivatives containing an amide moiety was synthesized based on fragment virtual screening to screen high-efficiency antibacterial agents for rice bacterial diseases. All target compounds showed greater bactericidal activity than commercial bactericides. 3-(4-fluorophenyl)-N-((5-(methylsulfonyl)-1,3,4-oxadiazol-2-yl)methyl)acrylamide (**10**) showed excellent antibacterial activity against *Xanthomonas oryzae* pv. *oryzae* and *Xanthomonas oryzae* pv*. oryzicola*, with EC_50_ values of 0.36 and 0.53 mg/L, respectively, which were superior to thiodiazole copper (113.38 and 131.54 mg/L) and bismerthiazol (83.07 and 105.90 mg/L). The protective activity of compound **10** against rice bacterial leaf blight and rice bacterial leaf streak was 43.2% and 53.6%, respectively, which was superior to that of JHXJZ (34.1% and 26.4%) and thiodiazole copper (33.0% and 30.2%). The curative activity of compound **10** against rice bacterial leaf blight and rice bacterial leaf streak was 44.5% and 51.7%, respectively, which was superior to that of JHXJZ (32.6% and 24.4%) and thiodiazole copper (27.1% and 28.6%). Moreover, compound **10** might inhibit the growth of *Xanthomonas oryzae* pv. *oryzae* and *Xanthomonas oryzae* pv*. oryzicola* by affecting the extracellular polysaccharides, destroying cell membranes, and inhibiting the enzyme activity of dihydrolipoamide S-succinyltransferase.

## 1. Introduction

Rice bacterial diseases threaten global food security due to their high frequency, serious damage, and difficulty to prevent and control [1,2,3]. Among them, rice bacterial leaf blight (RBLB) and rice bacterial leaf streak (RBLS), caused by *Xanthomonas oryzae pv. oryzae (Xoo)* and *Xanthomonas oryzae pv. oryzicola* (*Xoc)*, are two extremely destructive bacterial diseases that can cause 20–50% loss of agricultural production [4,5]. Today, traditional commercial bactericides, such as bismerthiazol and thiodiazole copper, are used to control RBLB and RBLS [6]. However, the long-term repeated use of traditional commercial bactericides has caused the control effect of bacteria to decrease and serious pollution to the environment to increase year by year [7,8,9], hence there is an urgent need for their replacement with new bactericides [10]. Therefore, the discovery of novel antibacterial agents is presently an urgent problem.

The research and development of new drugs is a long and complicated process, which requires significant resources and time costs. According to the report of the Tufts Center for the Study of Drug Development, the development of a new drug requires an average of 10–15 years, USD 1.4 billion in resource costs, and USD 1.16 billion in time costs [11,12]. As a mature drug design method, virtual screening to find candidate compounds has become the main method of drug design [13]. Fragment-based drug discovery (FBDD) could explore a larger chemical space through a smaller number of fragment compounds, discover molecules with higher binding efficiency, reduce the cost of drug development, and improve the efficiency of drug development [14,15]. Therefore, the fragment-based virtual screening guided discovery of new drugs is a research hotspot in the field of plant protection.

In our previous work, we found the sulfone candidate antibacterial agent JHXJZ [16]. Dihydrolipoamide *S*-succinyltransferase (DLST) was verified to be the target of JHXJZ by the click chemistry and quantitative chemical proteomic approach [17,18]. In this work, based on DLST, a model was established by rapidly identifying the non-effect fragments, thereby selectively replacing and optimizing the affinity of the drugs. A series of compounds was designed and synthesized, and some candidate drugs were selected. Then, their antibacterial activities against rice bacterial diseases were evaluated, and their mechanism of action was initially explored.

## 2. Results

### 2.1. Molecular Design

**Figure 1:**
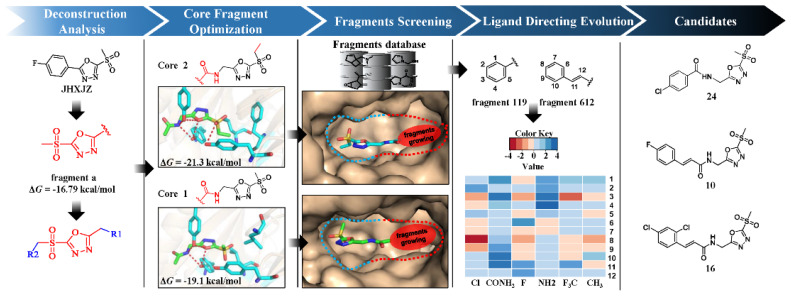
The fragment-based virtual screening: the structural optimization of JHXJZ.

JHXJZ was dismantled into fragments and their ligand efficiency (LE) was evaluated. Fragment **a** was chosen due to its better binding free energy (Δ*G*). Because changing the number of carbon atoms in the sulfone had a significant impact on the affinity, we chose compounds **a1** and **a2** (core **1** and core **2**) as promising starting core fragments for lead compound generation. Structure-based fragment virtual screening was conducted on core **1** and **2** in combination with DLST. Soon afterwards, the top 10 candidates with favorable ΔG values of the two core fragments were obtained. According to the feasibility of the synthesis and purification of the fragments, we considered whether it was possible to further synthesize and optimize. The six fragments were synthesized out together and the result showed that most of the fragments had excellent bactericidal activity except for the pyridine fragments. We believed that this type of structure had further enhanced value in the later stage, for instance, fragments **119** and **612**. Finally, fragments were determined according to the feasibility of the synthesis and derivatization of the fragments. Additionally, based on the combination mode of the fragments, we performed a single-replacement optimization scan and further optimized multiple-replacement optimization for the derivation of new compounds. From the later experimental results, compounds **24**, **10**, and **16**, which showed better bactericidal activity, had become the candidates. The construction process of this model based on DLST is shown in Figure 1.

**Figure 2:**
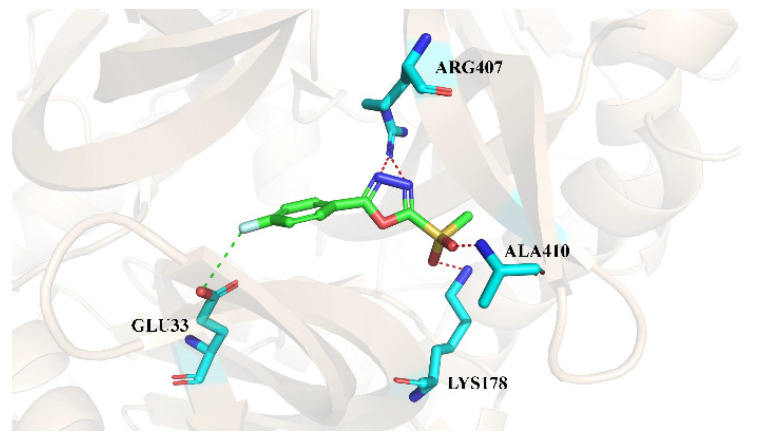
The binding mode of JHXJZ.

DLST was verified as the target of JHXJZ by the click chemistry and quantitative chemical proteomic approach. Therefore, we performed the fragment-based virtual screening based on JHXJZ (Figure 2). We found that the binding mode of JHXJZ was important to the structure-based lead optimization, and we predicted the binding mode of JHXJZ with DLST through molecular docking (Figure 2). Then, we analyzed the binding mode of JHXJZ to identify a prioritized pharmacophore. The stability of the binding mode was confirmed by a 10 ns molecular dynamic simulation. For DLST and JHXJZ, the binding mode was in an extended conformation and located in a hydrophobic pocket. Moreover, JHXJZ could form a hydrogen bond with ARG407, ALA410, and LYS178, and then form a halogen bond with GLU33. The JHXJZ bound away from the hydrophobic region that consisted of Arg180, Arg407, and Arg636.

Based on the binding mode of JHXJZ, we deconstructed JHXJZ into fragments and evaluated their ligand efficiency (LE). The binding free energy (Δ*G*) was determined using the Molecular Mechanics/Poisson–Boltzmann Surface Area (MM/PBSA) (Table 1). Fragment **a** showed the lowest binding free energy and the highest ligand efficiency (Δ*G* = −16.79 kcal/mol, LE = 1.87). Fragment **c** showed the lowest ligand efficiency and the highest binding free energy (Δ*G* = −6.55 kcal/mol, LE = 0.81). Hence, fragment **c** was a portion incapable of contributing binding free energy, and the compound showed modification potential for structural evolution. The structural modification was performed on fragment **a** to optimize binding affinity. Typically, the addition of non-bond interactions and hydrophobic interactions can effectively improve the affinity (Table 2). It was noticed that changing the number of carbon atoms in the sulfone had a significant impact on the affinity (**a1**, Δ*G* = −19.11 kcal/mol, LE = 1.47; **a2**, Δ*G* = −21.31 kcal/mol, LE = 1.52). Therefore, we chose compounds **a1** and **a2** (core **1** and core **2**) as promising starting core fragments for lead compound generation.

Structure-based fragment virtual screening was conducted on core **1** and **2** in combination with DLST. All linked fragments were refined. The top 10 candidates with favorable Δ*G* values of the two core fragments were obtained (Table 3). According to the feasibility of the synthesis and purification of the fragments, we considered whether it was possible to further synthesize and optimize. In the process of screening the fragments, we tried to synthesize the target compounds. The six fragments were synthesized out together and the result showed that most of fragments had excellent bactericidal activity except for the pyridine fragments (Table 4). Among them, according to the feasibility of the synthesis and derivatization of the fragments, we believed that fragments **119** and **612** had the potential for further optimization. To further optimize the binding free energy of compounds **9** and **11**, the ligand-directing evolution strategy was performed using the AILDE web server. A series of compounds was generated based on compounds **9** and **11**, and the top 10 candidates with favorable ΔΔ*G* values are shown in the Supplementary Materials. In total, 26 compounds were synthesized by ranking the binding energy and synthetic accessibility, and the antibacterial activity of all the target compounds was evaluated (Table 4, Tables S1 and S2).

**Figure 3:**
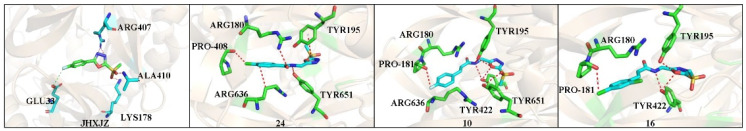
The binding modes of JHXJZ and compounds **24**, **10**, and **16**.

Among target compounds **1–26**, compounds **24**, **10**, and **16** showed better bactericidal activity. The binding modes of JHXJZ and compounds **24**, **10**, and **16** against DLST are shown in Figure 3. Compared to the binding mode of JHXJZ, compounds **24**, **10**, and **16** formed hydrogen bonds with TYR195 and TRY422, and formed pi stacking with TYR195. In addition, a stronger hydrophobic interaction was formed due to molecular collisions with Arg180/407/636. We believe that this computational framework might be used to eliminate non-effect fragments and optimize binding affinity.

### 2.2. Chemicals

**Figure 4:**
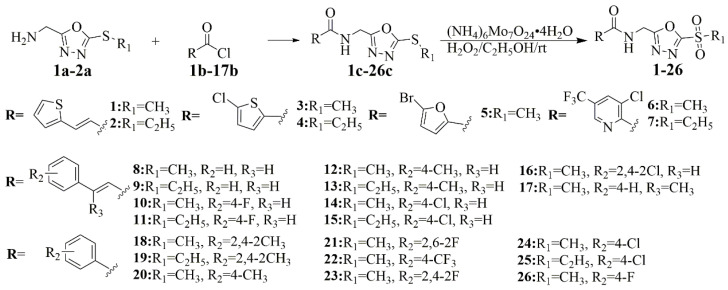
Synthesis route of compounds **1**–**26**.

The different substituted oxadiazole thioethers (intermediates **1a**–**2a**) were prepared by glycine ethyl ester hydrochloride, di-tert-butyl carbonate, N_2_H_4_∙H_2_O, CS_2_, bromide, and trifluoroacetic acid. Different acids containing aromatic groups were added to SOCl_2_ and refluxed to obtain intermediates **1b**–**17b**. The intermediates **1c**–**26c** were oxidized by (NH_4_)_6_Mo_7_O_24_·4H_2_O and hydrogen peroxide solution to obtain the target compounds **1**–**26** (Figure 4). The details of ^1^H NMR, ^13^C NMR, physicochemical property, melting point, yield, and HRMS are provided in the Supplementary Materials.

### 2.3. Antibacterial Activity In Vitro Test

All the target compounds showed excellent antibacterial activity against *Xoo* at 50 and 10 mg/L (Table S3). In addition to compounds **2** and **7**, the antibacterial activity of all target compounds against *Xoo* was greater than 90% at 10 mg/L. Interestingly, the antibacterial activity of compound **9** was 97.6% at 50 mg/L. However, when the concentration was reduced to 10 mg/L, the antibacterial activity of compound **9** was 100%. This difference may be related to the solubility of compound **9**. When the antibacterial activity was tested, we found that the solution with a concentration of 50 mg/L of compound **9** was turbid. However, the solution of compound **9** was transparent at 10 mg/L. The EC_50_ values of compounds **1**–**26** against *Xoo* were 0.36–7.45 mg/L, which were superior to those of bismerthiazol (83.07 mg/L) and thiodiazole copper (113.38 mg/L). Among them, 24 compounds showed EC_50_ values less than 1.0 mg/L. For example, the EC_50_ values of compounds **4**, **10**, **14**, **15**, **16**, and **24** against *Xoo* were 0.45, 0.36, 0.43, 0.43, 0.42, and 0.40 mg/L, respectively. 

Meanwhile, all the target compounds showed good antibacterial activity against *Xoc* at 50 and 10 mg/L (Table S4). The antibacterial activity of compounds **1**–**26** was greater than 90% at 50 mg/L. In addition, the EC_50_ values of compounds **1**–**26** against *Xoc* were 0.53–10.77 mg/L, which were superior to those of bismerthiazol (105.90 mg/L) and thiodiazole copper (131.54 mg/L). Among them, 10 compounds showed EC_50_ values less than 1.0 mg/L. For example, the EC_50_ values of compounds **8**, **9**, **10**, **12**, and **14** against *Xoc* were 0.70, 0.78, 0.53, 0.64, and 0.61 mg/L, respectively.

### 2.4. Antibacterial Activity In Vivo Test

The antibacterial activity of compound **10** against RBLB and RBLS was evaluated (Figure S1 and Table 5). The protective activity of compound **10** against RBLB and RBLS was 43.2% and 53.6%, respectively, which was superior to that of JHXJZ (34.1% and 26.4%) and thiodiazole copper (33.0% and 30.2%). The curative activity of compound **10** against RBLB and RBLS was 44.5% and 51.7%, respectively, which was superior to that of JHXJZ (32.6% and 24.4%) and thiodiazole copper (27.1% and 28.6%).

### 2.5. Enzyme Activity Detection of DLST

**Figure 5:**
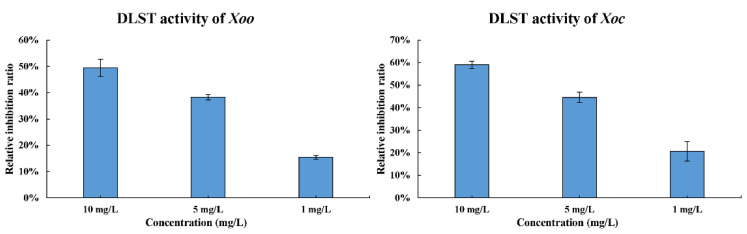
DLST activity of Xoo and Xoc at 10, 5, and 1 mg/L.

DLST was found as the main target of JHXJZ in *Xoo*. JHXJZ may affect the cells by regulating the lysine succinyl modification level energy metabolism process [17,18]. The inhibitory activity of compound **10** on DLST was evaluated (Figure 5). After compound **10** treated *Xoo* and *Xoc*, the relative inhibition rates of DLST were 49.5% and 59.1% at 10 mg/L, respectively. However, when the concentration was reduced to 1 mg/L, the relative inhibition rates of DLST were 15.5% and 20.7%, respectively.

### 2.6. Biofilm Formation

**Figure 6:**
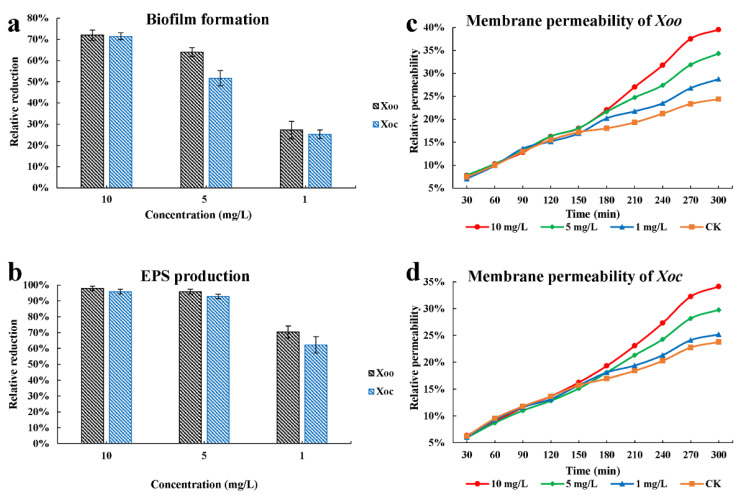
Effects of *Xoo* and *Xoc* on the biofilm formation (**a**), extracellular polysaccharide (EPS) production (**b**), and membrane permeability (**c**,**d**) at 10, 5, and 1 mg/L.

The inhibitory activity of compound **10** on the biofilm formation of *Xoo* and *Xoc* was evaluated (Figure 6). At the concentrations of 10, 5, and 1 mg/L, the inhibition rates of biofilm formation were 72.0%, 64.0%, and 27.2%, and 71.5%, 51.7%, and 25.2%, respectively. Therefore, compound **10** might affect the activity of bacteria through inhibiting the formation of biofilm.

### 2.7. Extracellular Polysaccharide Production

The inhibitory activity of compound **10** on the extracellular polysaccharide production of *Xoo* and *Xoc* was evaluated (Figure 6). The inhibition rates of extracellular polysaccharide production were 98.0%, 95.9%, and 70.4%, and 95.9%, 92.9%, and 62.2% at 10, 5, and 1 mg/L, respectively. Therefore, compound **10** might destroy the normal reproductive cycle of bacteria by inhibiting the production of extracellular polysaccharides.

### 2.8. Membrane Permeability

The effects of compound **10** on the cell membrane permeability of *Xoo* and *Xoc* at 10, 5, and 1 mg/L were determined (Figure 6). After 30 min, the cell membrane permeability increased as the treatment time increased. The cell membrane permeability of the different treatment groups and the negative control group did not differ before 180 min. However, after 180 min, the different treatment groups and the cell membrane permeability of the negative control group showed differential effects and had concentration dependence. 

### 2.9. Morphological Change in Bacteria 

**Figure 7:**
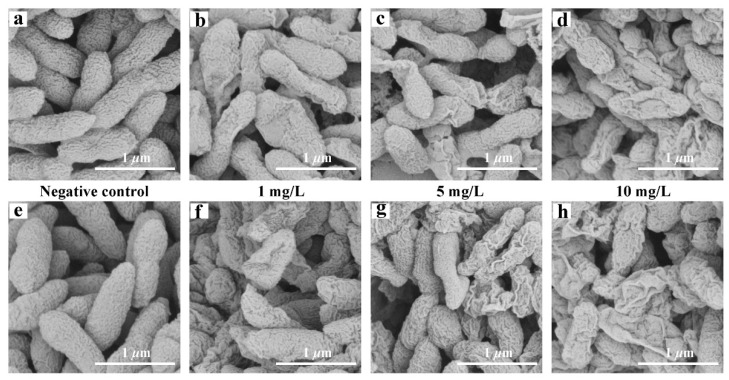
Changes in bacterial morphology of *Xoo (***a**–**d***)* and *Xoc (***e**–**h***)* at 10, 5, and 1 mg/L.

The morphological changes of *Xoo* and *Xoc* were observed by the scanning electron microscope (Figure 7). The cell shapes of the negative control groups of *Xoo* and *Xoc* were full and there were no obvious wrinkles on the surface. At the concentrations of 1, 5, and 10 mg/L of compound **10**, wrinkles and deformations appeared on the cell surface, and the degree of wrinkles and deformation gradually increased as the concentration increased. 

## 3. Discussion

In summary, via fragment-based virtual screening, a series of new oxadiazole sulfone derivatives was synthesized to screen high-efficiency antibacterial agents for rice bacterial diseases. Interestingly, all synthetic target compounds showed excellent antibacterial activities against *Xoo* and *Xoc*. Compound **10** showed good antibacterial activity in vivo against RBLB and RBLS. Compound **10** showed good inhibitory activity against DLST, which indicated that DLST might be the target of compound **10** in *Xoo* and *Xoc*. In addition, compound **10** might suppress the growth of *Xoo* and *Xoc* by inhibiting the formation of biofilm and the production of extracellular polysaccharides and changing the cell membrane permeability and cell surface morphology. The method of compounds’ structural design based on fragment virtual screening can improve the efficiency of finding highly active compounds. In addition, compound **10** can be studied as a potential antibacterial agent in the future.

## 4. Materials and Methods

### 4.1. Molecular Design

#### 4.1.1. Molecular Docking and Dynamics Simulation

Molecular docking was performed using AutoDock 4.2. The protein crystal was generated on BLAST in Uniprot (A0A0K0GL90) and built by Swiss–Model [19,20]. The homology modeling template 1C4T showed 65.8% sequence identity [21]. The protein structures were prepared by removing the water molecules and then adding hydrogen atoms and repairing the side chains. JHXJZ was docked into DLST, and 10 poses were exported for further analysis. The molecular dynamic was performed using AMBER 16 [22]. The dominant conformation was confirmed by 10 ns of dynamic trajectory analysis, and the binding energy calculation was conducted via the MM–PBSA method [23].

#### 4.1.2. Fragment-Based Virtual Screening

Fragment-based virtual screening was performed using the ACFIS web server (http://chemyang.ccnu.edu.cn/ccb/server/ACFIS/ 11 January 2021). The selected compound, in combination with DLST as the starting structure, was linked to the refined database containing over 1500 fragments. The Δ*G* value of each newly generated compound was calculated by the MM–PBSA method after a minimization. 

#### 4.1.3. Ligand-Directing Evolution

The ligand-directing evolution was performed using the AILDE web server (http://chemyang.ccnu.edu.cn/ccb/server/AILD 15 January 2021) based on the result of the fragment virtual screening. Every hydrogen atom of the selected favored fragment was replaced by the 10 most used substituents (–CH_3_, –OH, –F, –Cl, –Br, –CONH_2_, –CF_3_, –NH_2_, –NO_2_, and –OCH_3_) to generate the potential compounds in each snapshot. We used the MD simulation to refine the newly generated receptor–ligand complex to obtain the final structure. The binding free energy (∆*G*) of the refined complex structures was evaluated using the MM–PBSA method.

### 4.2. Chemicals

The synthesis processes of the intermediates and target compounds were supervised through thin-layer chromatography (TLC). Using an XT-4 binocular microscope (Beijing Tech Instrument Co., Beijing, China), the melting points were measured. ^13^C and ^1^H NMR spectra were obtained by a Bruker Ascend–400 spectrometer (Bruker, Karlsruhe, Germany). The HRMS data were acquired by a Thermo Scientific Q Exactive (Thermo Scientific, Waltham, MA, USA).

General Procedures for the Preparation of Compounds **1**–**26**. The different oxadiazole intermediates **1a**–**2a** were synthesized according to known methods [23]. Different acids containing aromatic groups were added to SOCl_2_ (5–8 mL) and refluxed for 5–8 h to obtain intermediates **1b**–**17b**. Next, the triethylamine (3.8 mmol) and intermediates **1a**–**2a** (2.5 mmol) were added to CH_2_Cl_2_ in ice–water bath conditions. The intermediates **1b**–**17b** were added and stirred for 2.5–5 h. Then, the intermediates **1c**–**26c** were obtained by silica gel column chromatography. Finally, the intermediates **1c**–**26c** (1.5 mmol), (NH_4_)_6_Mo_7_O_24_·4H_2_O (0.3 mmol), and hydrogen peroxide solution (30%, 15 mmol) were admixed and stirred at 25 °C for 3–8 h. The saturated ice saltwater was added to the wash, and the target compounds **1**–**26** were obtained with a yield of 52–89% (Figure 4). The details of compounds **1**–**26** can be found in the Supplementary Materials.

### 4.3. Antibacterial Activity In Vitro Test

The antibacterial activities in vitro of compounds **1**–**26** against *Xoo* and *Xoc* were evaluated according to our previously reported method [24]. The compounds were dissolved in DMSO, and the solutions were diluted with 0.1% Tween 20 and nutrient broth (NB, 1%) media to prepare different concentrations of the solutions. After shaking the bacteria at 28 °C for 1–2 d, the inhibition rates were calculated—by comparing differences of the OD_595_ values between the treatment group and negative controls by a microplate spectrophotometer. The test was repeated three times.

### 4.4. Antibacterial Activity In Vivo Test

The antibacterial activities of compound **10** against RBLB and RBLS were evaluated at 200 mg/L using the leaf-cutting and needleless injector method according to the reported method [25]. The NB (1%) media containing *Xoo* and *Xoc* at the logarithmic growth period were inoculated on rice leaves. In the protective activity, the agent was sprayed onto rice blades, and bacteria were inoculated after 24 h. In the curative activity, the bacteria were inoculated into the rice leaves, and the test compounds were sprayed after 24 h. The antibacterial activities were calculated by the disease index and lesion length of rice leaves at 2 weeks post-spraying.

### 4.5. Enzyme Activity Detection of DLST

The inhibitory activity of compound **10** against DLST was tested according to the previously reported method and the instructions of the enzyme activity kit [17,18]. The *Xoo* and *Xoc* bacteria solutions at the logarithmic growth period were added to the NB medium (5 mL) and shaken at 28 °C until the OD_600_ value reached 0.3. Compound **10** and JHXJZ, at the final concentrations of 10, 5, and 1 mg/L, were mixed into the bacterial suspension, and the mixture was continuously shaken for 12 h. The solution without the compound was used as the negative control in the same experimental conditions. *Xoo* and *Xoc* were cultivated to a logarithmic growth phase (the negative control). Then, the bacterial solution was collected by centrifugation. The DLST proteins of *Xoo* and *Xoc* were extracted by the method described in the kit. Soon afterward, the enzyme activity of DLST was tested by a microplate spectrophotometer (Shanghai Enzyme Link Biotechnology Co., Ltd. Suzhou, China) using the enzyme assays kits.

### 4.6. Biofilm Formation

The effects of compound **10** on the biofilm formation of *Xoo* and *Xoc* were determined according to the previously reported method [26]. Compound **10** and bacteria with a logarithmic growth phase were mixed to prepare solutions of 10, 5, and 1 mg/L, respectively. The bacteria were poured out after standing for 5 d at 28 °C. Next, the crystalline violet (0.1%, *w*/*v*) was added. The ethanol (2 mL) was added to dissolve the crystal violet on the bottle wall. The inhibition activities were calculated by the difference in the OD_590_ values.

### 4.7. Membrane Permeability

The effects of compound **10** on the membrane permeability of *Xoo* and *Xoc* were tested according to the previously reported works [27,28]. *Xoo* and *Xoc*, both with a logarithmic growth phase, were collected by centrifugation. Compound **10** was prepared as solutions of 10, 5 and 1 mg/L, respectively, and the conductivities were measured at 0–300 min.

### 4.8. Extracellular Polysaccharide Production

According to our previously reported work, the effects of compound **10** on the extracellular polysaccharide (EPS) production of *Xoo* and *Xoc* were tested at 10, 5, and 1 mg/L [29]. After shaking at 28 °C for 3 d, the bacteria were centrifuged, and anhydrous ethanol was added to obtain the deposit after 12 h. The EPS production was acquired by centrifugation, drying, and weighing.

### 4.9. Morphological Change in Bacteria 

According to the previously reported method [30,31,32], the changes in bacterial cell morphology were observed via scanning electron microscopy (FEI, Hillsboro, OR, USA). The details of the assay were adequately described in the published previous paper.

## Figures and Tables

**Table 1:** The deconstruction analysis of JHXJZ.

Compound	Structure	Δ*G*	LE
JHXJZ	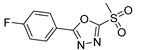	−25.68	1.60
a	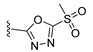	−16.79	1.87
b	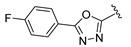	−10.33	0.86
c	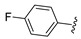	−6.55	0.81
d	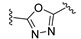	−7.67	1.53

**Table 2:** The optimization analysis of compound **a**.

Compound	Structure	Δ*G*	LE
a	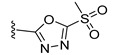	−16.79	1.87
a1	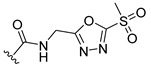	−19.11	1.47
a2	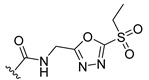	−21.31	1.52
a3	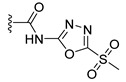	−16.11	1.34
a4	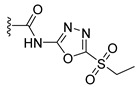	−17.54	1.35
a5	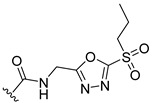	−17.91	1.19

**Table 3:** The fragment structure generated from core**1** and **2** via ACFIS webserver.

Fragment	Structure	Δ*G*	Fragment	Structure	Δ*G*
core1	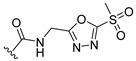	−19.11	core2	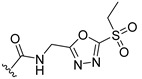	−21.31
858	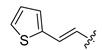	−29.69	677	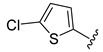	−31.70
677	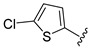	−28.15	1247	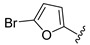	−30.59
612	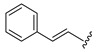	−27.48	288	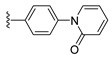	−29.36
1247	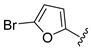	−26.55	858	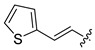	−29.19
119	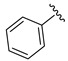	−26.44	1347	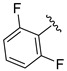	−28.85
708	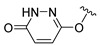	−26.15	119	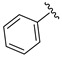	−28.76
1347	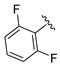	−26.02	612	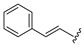	−28.71
1523	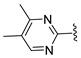	−26.01	260	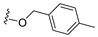	−28.65
1549	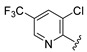	−24.94	254	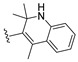	−27.81
266	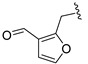	−24.58	1549	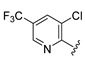	−27.47

**Table 4:** The six screened fragments.

Number	Compound	Fragment	*n*	Δ*G*	EC_50_
**1**	**1**	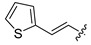	0	−29.69	0.68
**2**	**2**		1	−29.19	0.72
**3**	**3**	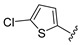	0	−28.15	0.53
**4**	**4**		1	−31.70	0.45
**5**	**5**	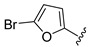	0	−26.55	0.48
**6**			1	−30.59	−
**7**	**6**	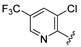	0	−24.94	3.49
**8**	**7**		1	−27.47	7.45
**9**	**8**	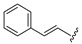	0	−27.48	0.73
**10**	**9**		1	−28.71	0.89
**11**		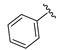	0	−26.44	−
**12**			1	−28.76	−

**Table 5:** Protective and curative activities of compound 10 against two rice bacterial diseases at 200 mg/L.

	Rice Bacterial Leaf Blight		Rice Bacterial Leaf Streak	
Treatment Group	Protective Activity (%)	Curative Activity (%)	Protective Activity (%)	Curative Activity (%)
10	43.2 ± 5.8	44.5 ± 2.7	53.6 ± 1.8	51.7 ± 3.5
JHXJZ	34.1 ± 1.6	32.6 ± 1.5	26.4 ± 5.2	24.4 ± 2.2
BT	39.8 ± 1.6	38.0 ± 2.7	47.4 ± 3.5	45.2 ± 3.3
TC	33.0 ± 3.2	27.1 ± 3.1	30.2 ± 4.4	28.6 ± 5.0

## Data Availability

Characterizations, physical, analytical, and bactericidal activity data of target compounds **1**–**26** were showed in Supplementary Materials.

## Supplementary Materials

The following are available online at https://www.mdpi.com/article/10.3390/ijms222312953/s1.

## Abbreviations

NMR, nuclear magnetic resonance; HRMS, high-resolution mass spectrometry; MM/PBSA, molecular mechanics/Poisson–Boltzman surface area; LE, ligand efficiency; FBDD, fragment-based drug discovery.
