# Supplementary material for: Discovery of Novel Dihydrolipoamide S-Succinyltransferase Inhibitors Based on Fragment Virtual Screening
Source: Int J Mol Sci. 2021 Nov 30;22(23):12953. doi: 10.3390/ijms222312953 (PMC8657855; doi:10.3390/ijms222312953)
Supplement: Supplementary file 1 [file ijms-22-12953-s001.zip › ijms-1447009-supplementary.pdf]

Supplementary Information

Discovery of Novel Dihydrolipoamide S-Succinyltransferase Inhibitors Based on Fragment Virtual Screening

Chengqian Wei, Junjie Huang, Yu Wang, Yifang Chen, Xin Luo, Shaobo Wang, Zengxue Wu, Jixiang Chen *

State Key Laboratory Breeding Base of Green Pesticide and Agricultural Bioengineering, Key Laboratory of Green Pesticide and Agricultural Bioengineering, Ministry of Education, Guizhou University, Huaxi District, Guiyang 550025, China; E-Mails: wcq1996@163.com (C.W.); hjj18744902440@163.com (J.H); gs.wy20@gzu.edu.cn (Y.W); gs.chenyf20@gzu.edu.cn (Y.C.); gs.xinluo20@gzu.edu.cn (X.L); wangshaobo97@163.com (S.W.); wuzx@gzu.edu.cn (Z.W.);

* Correspondence: jxchen@gzu.edu.cn

Contents

Table S1. The top ten candidates with favorable ΔΔG values. 1

Table S1. The top ten candidates with favorable ΔΔG values. 2

Table S3**.** The antibacterial activity of compounds against *Xoo*. 3

Table S4. The antibacterial activity of compounds against Xoc. 4

Figure S1**.** Curative and protective activities of compound **10** against RBLB and RBLS at 200 mg/L. 5

^{1}H NMR, ^{13}C NMR and HRMS spectrum of title compounds **1–26** 6

Table S1. The top ten candidates with favorable ΔΔG values.

**Num.**	**Compound**	**n**	**R1**	**R2**	**R3**	**R4**	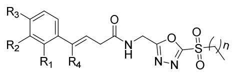	**ΔΔ*G***	**EC_{50}**
**13**	**10**	0	/	/	F	/	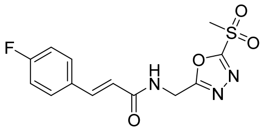	-1.02	0.36
**14**	**11**	1	/	/	F	/	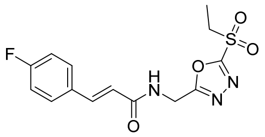	-1.14	0.72
**15**	**12**	0	/	/	CH3	/	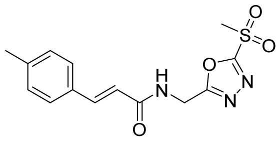	-1.41	0.52
**16**	**13**	1	/	/	CH3	/	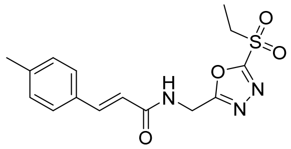	-1.35	0.72
**17**	**14**	0	/	/	Cl	/	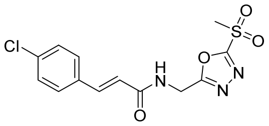	-2.31	0.43
**18**	**15**	1	/	/	Cl	/	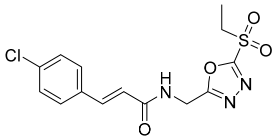	-3.23	0.43
**19**	**16**	0	Cl	/	Cl	/	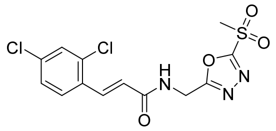	-2.55	0.42
**20**		1	Cl	/	Cl	/		-2.64	-
**21**	**17**	0	/	/	/	CH3	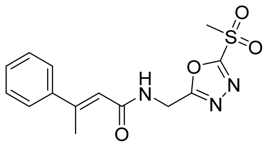	-0.98	0.54
**22**		1	/	/	/	CH3		-1.04	-

Table S2. The top ten candidates with favorable ΔΔ*G* values.

Num.	Compound	n	R1	R2	R3	R4	R5	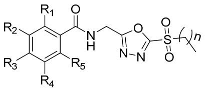	ΔΔ*G*	EC_{50}
**23**	**18**	0	/	/	CH3	/	CH3	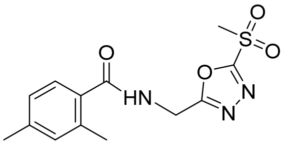	-0.85	0.69
**24**	**19**	1	/	/	CH3	/	CH3	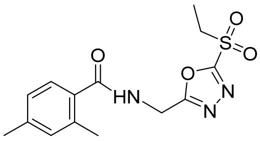	-1.03	1.08
**25**	**20**	0	/	/	CH3	/	/	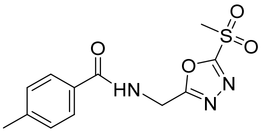	-0.77	0.63
**26**	**21**	0	F	/	/	/	F	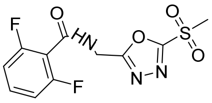	-1.79	0.65
**27**	**22**	0	/	/	CF3	/	/	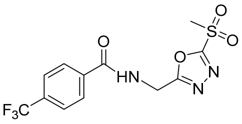	-3.23	0.72
**28**	**23**	0	F	/	F	/	/	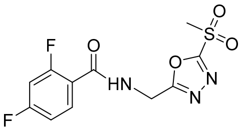	-1.56	0.80
**29**	**24**	0	/	/	Cl	/	/	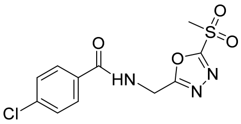	-1.07	0.40
**30**	**25**	1	/	/	Cl	/	/	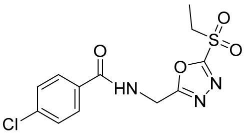	-1.22	0.82
**31**	**26**	0	/	/	F	/	/	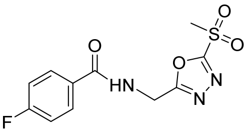	-1.37	0.61
**32**		1	/	/	F	/	/		-1.55	-

Table S3. The antibacterial activity of compounds against *Xoo*.

Compound	Inhibition (%)		R^2	EC_{50} [a] (mg/L)
	50 (mg/L)	10 (mg/L)		
**1**	97.6±1.2	96.2±1.5	0.91	0.68±0.07
**2**	90.6±8.6	85.0±4.1	0.97	0.72±0.07
**3**	95.8±2.6	98.6±0.8	0.92	0.53±0.07
**4**	97.9±1.5	98.3±1.0	0.94	0.45±0.04
**5**	96.6±0.4	96.3±2.5	0.98	0.48±0.01
**6**	99.7±0.2	94.8±1.8	0.95	3.49±0.07
**7**	99.8±0.1	48.1±0.8	0.95	7.45±0.16
**8**	98.9±0.5	99.7±0.3	0.95	0.73±0.08
**9**	97.6±1.4	100	0.95	0.89±0.12
**10**	99.5±0.1	99.0±0.3	0.92	0.36±0.03
**11**	99.2±0.4	98.8±0.1	0.96	0.72±0.05
**12**	99.8±0.2	99.5±0.1	0.94	0.52±0.04
**13**	99.2±0.2	99.5±0.1	0.96	0.72±0.07
**14**	98.7±0.3	97.6±0.8	0.95	0.43±0.06
**15**	98.3±0.2	98.1±0.6	0.93	0.43±0.04
**16**	99.0±0.4	98.9±0.3	0.94	0.42±0.05
**17**	99.7±0.4	99.4±0.5	0.96	0.54±0.04
**18**	98.2±1.4	98.8±0.2	0.97	0.69±0.04
**19**	98.1±0.8	97.2±1.4	0.96	1.08±0.11
**20**	99.6±0.3	99.4±0.3	0.97	0.63±0.06
**21**	96.3±0.4	97.4±0.3	0.95	0.65±0.05
**22**	99.9±0.1	99.7±0.2	0.96	0.72±0.05
**23**	95.4±0.4	95.7±1.9	0.95	0.80±0.09
**24**	99.6±0.2	99.9±0.1	0.91	0.40±0.03
**25**	99.2±0.3	98.7±0.1	0.98	0.82±0.05
**26**	99.1±0.1	99.4±0.2	0.95	0.61±0.03
**JHXJZ**	94.5±2.4	98.7±0.5	0.93	1.35±0.16
**BT**[b]	27.4±5.0	2.5±1.6	0.97	83.07±8.07
**TC**[c]	22.3±4.3	7.9±4.4	0.97	113.38±8.22

[a]Averages of three replicates. [b]Bismerthiazol and [c]Thiodiazole copper were used for a comparison of activities.

Table S4. The antibacterial activity of compounds against *Xoc*.

Compound	Inhibition (%)		R^2	EC_{50} [a] (mg/L)
	50 (mg/L)	10 (mg/L)		
**1**	98.3±0.9	97.3±1.8	0.97	0.86±0.08
**2**	97.5±0.8	96.9±0.3	0.97	3.41±0.05
**3**	98.3±1.3	98.0±0.2	0.98	1.06±0.04
**4**	99.0±0.2	96.9±1.1	0.97	0.83±0.01
**5**	97.8±2.0	99.3±0.1	0.92	2.59±0.05
**6**	99.3±0.2	95.5±0.8	0.99	3.31±0.09
**7**	99.6±0.2	40.1±1.3	0.95	7.58±0.27
**8**	92.3±7.3	98.8±0.7	0.91	0.70±0.01
**9**	98.0±1.7	97.1±1.6	0.97	0.78±0.01
**10**	95.6±1.0	99.0±0.8	0.94	0.53±0.01
**11**	99.0±1.3	50.6±2.1	0.94	10.77±2.91
**12**	95.6±2.1	96.9±2.3	0.98	0.64±0.05
**13**	95.8±1.2	98.1±0.6	0.99	0.88±0.03
**14**	98.2±1.1	98.8±0.7	0.96	0.61±0.03
**15**	98.9±0.3	97.9±0.4	0.94	1.45±0.05
**16**	97.7±0.7	99.2±0.7	0.94	1.15±0.05

**17**	94.7±0.9	67.7±0.9	0.91	5.21±0.11
**18**	96.9±0.2	98.9±1.0	0.92	1.74±0.11
**19**	95.8±0.8	98.2±1.0	0.96	1.69±0.18
**20**	99.0±0.9	67.8±6.8	0.97	1.66±0.21
**21**	98.4±1.1	97.1±2.3	0.96	1.51±0.02
**22**	99.3±0.3	90.3±5.5	0.96	1.70±0.09
**23**	99.5±0.4	69.6±1.8	0.91	7.41±1.30
**24**	97.8±2.7	96.9±0.4	0.99	0.90±0.04
**25**	98.8±1.0	99.6±0.3	0.96	0.94±0.09
**26**	99.0±1.0	98.3±1.6	0.91	2.30±0.06
**JHXJZ**	97.0±2.1	51.0±0.8	0.90	7.43±0.28
**BT**[b]	27.0±1.7	7.0±1.7	0.92	105.90±0.56
**TC**[c]	30.5±2.4	13.0±3.3	0.98	131.54±9.99

[a]Averages of three replicates. [b]Bismerthiazol and [c]Thiodiazole copper were used for a comparison of activities.

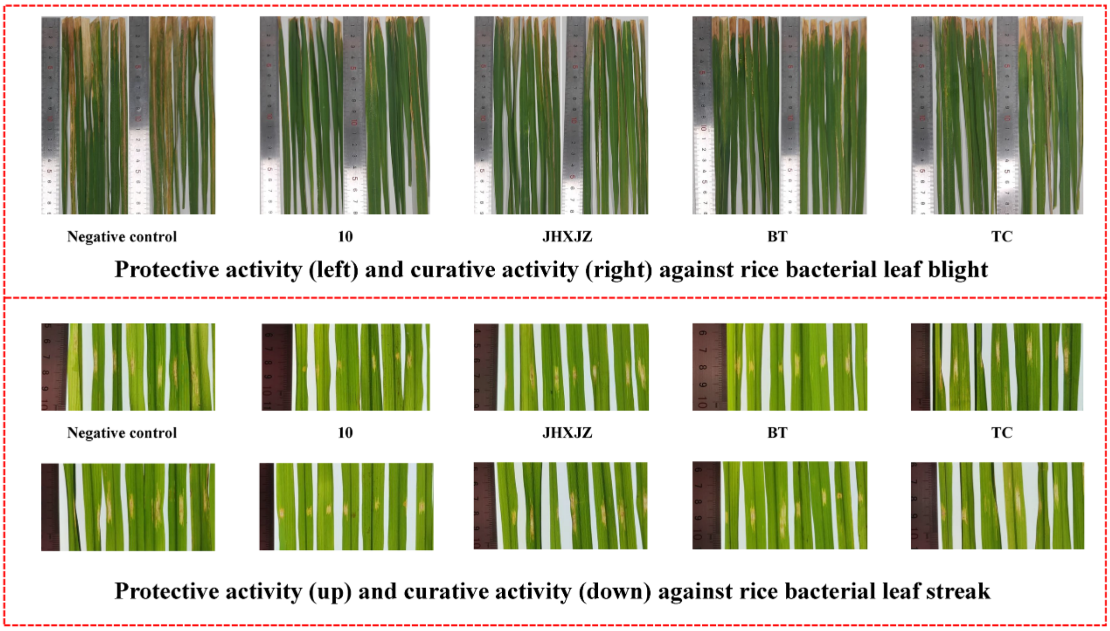

Figure S1. Curative and protective activities of compound **10** against RBLB and RBLS at 200 mg/L.

^{1}H NMR, ^{13}C NMR and HRMS spectrum of title compounds 1–26

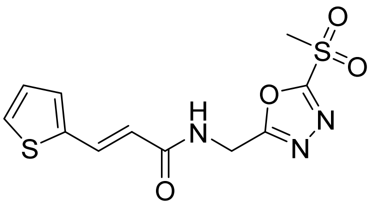

N-((5-(methylsulfonyl)-1,3,4-oxadiazol-2-yl)methyl)-3-(thiophen-2-yl)acrylamide **(1)**. White solid, m.p. 134.2–134.9 °C, yield, 72%; ^{1}H NMR (400 MHz,

DMSO-d_6) δ 8.98 (t, J = 5.5 Hz, 1H, -CONH-), 7.75 – 7.57 (m, 2H, Ar-H), 7.43 (d, J = 3.5 Hz, 1H, Ar-H), 7.16 – 7.09 (m, 1H, -CH=), 6.45 (d, J = 15.6 Hz, 1H, -COCH=), 4.76 (d, J = 5.6 Hz, 2H, -CH_2-), 3.68 (s, 3H, -SO_2CH_3). ^{13}C NMR (100 MHz, DMSO-d_6) δ 167.15, 165.73, 162.89, 139.91, 133.69, 131.82, 129.00, 128.92, 119.76, 43.17, 34.60. HRMS (ESI): calcd for ($C_{11}H_{11}O_4N_3S_2Na$) ([M+Na]$^+$), 336.00832; found, 336.00803.

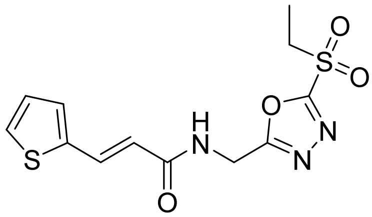

N-((5-(ethylsulfonyl)-1,3,4-oxadiazol-2-yl)methyl)-3-(thiophen-2-yl)acrylamide **(2)**. White solid, m.p. 149.0–149.6 °C, yield, 68%; ^{1}H NMR (400 MHz, DMSO-d_6) δ 8.97 (t, J = 5.6 Hz, 1H, -CONH-), 7.68 – 7.64 (m, 2H, Ar-H), 7.44 – 7.43 (m, 1H, Ar-H), 7.14 – 7.12 (m, 1H, -CH=), 6.44 (d, J = 15.6 Hz, 1H, -COCH=), 4.76 (d, J = 5.7 Hz, 2H, -CONH$\underline{CH_2}$-), 3.76 (q, J = 7.3 Hz, 2H, -SO_2CH_2-), 1.29 (t, J = 7.3 Hz, 3H, -CH_3). ^{13}C NMR (100 MHz, DMSO-d_6) δ 167.56, 165.75, 161.75, 139.91, 133.68, 131.83, 129.01, 128.92, 119.73, 49.82, 34.71, 7.11. HRMS (ESI): calcd for ($C_{12}H_{13}O_4N_3S_2Na$) ([M+Na]$^+$), 350.02397; found, 350.02350.

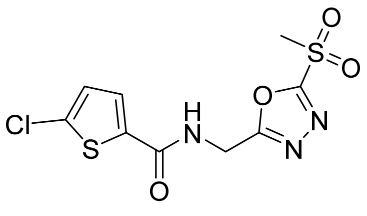

5-chloro-N-((5-(methylsulfonyl)-1,3,4-oxadiazol-2-yl)methyl)thiophene-2-carboxamide **(3)**. White solid, m.p. 156.1–156.8 °C, yield, 73%; ^{1}H NMR (400 MHz,

DMSO-d_6) δ 9.56 (t, J = 5.5 Hz, 1H, -CONH-), 7.78 (d, J = 4.1 Hz, 1H, Ar-H), 7.31 (d, J = 4.0 Hz, 1H, Ar-H), 4.87 (d, J = 5.6 Hz, 2H, -CONHCH$_2$-), 3.74 (s, 3H, -CH$_3$). ^{13}C NMR (100 MHz, DMSO-d_6) δ 166.86, 162.95, 161.06, 137.94, 134.35, 129.53, 128.86, 43.16, 34.87. HRMS (ESI): calcd for ($C_9H_8O_4N_3ClS_2Na$) ([M+Na]$^+$), 343.95370; found, 343.95328.

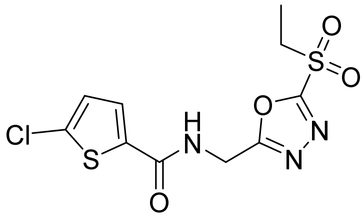

5-chloro-N-((5-(ethylsulfonyl)-1,3,4-oxadiazol-2-yl)methyl)thiophene-2-carboxamide **(4)**. White solid, m.p. 136.8–137.7 °C, yield, 65%; ^{1}H NMR (400 MHz, DMSO-d_6) δ 9.49 (t, J = 5.5 Hz, 1H, -CONH-), 7.71 (d, J = 4.1 Hz, 1H, Ar-H), 7.25 (d, J = 4.1 Hz, 1H, Ar-H), 4.81 (d, J = 5.6 Hz, 2H, -CONHCH$_2$-), 3.77 (q, J = 7.3 Hz, 2H, -SO$_2$CH$_2$-), 1.29 (t, J = 7.3 Hz, 3H, -CH$_3$). ^{13}C NMR (100 MHz, DMSO-d_6) δ 167.27, 161.83, 161.08, 137.91, 134.35, 129.53, 128.86, 49.83, 34.97, 7.10. HRMS (ESI): calcd for ($C_{10}H_{10}O_4N_3ClS_2Na$) ([M+Na]$^+$), 357.96935; found, 357.96881.

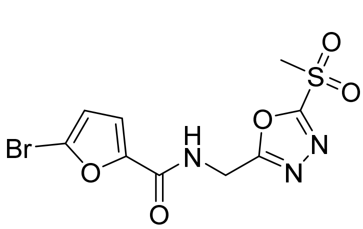

5-bromo-N-((5-(methylsulfonyl)-1,3,4-oxadiazol-2-yl)methyl)furan-2-carboxamide **(5)**. White solid, m.p. 142.5–143.1 °C, yield, 64%; ^{1}H NMR (400 MHz, DMSO-d_6) δ 9.37 (t, J = 5.7 Hz, 1H, -CONH-), 7.30 (d, J = 3.6 Hz, 1H, Ar-H), 6.88 (d, J = 3.6 Hz, 1H, Ar-H), 4.84 (d, J = 5.7 Hz, 2H, -CONHCH$_2$-), 3.74 (s, 3H,

-CH_3). ^{13}C NMR (100 MHz, DMSO-*d*6) *δ* 166.84, 162.93, 157.39, 149.16, 125.79, 117.54, 114.76, 43.17, 34.45. HRMS (ESI): calcd for ($C_9H_8O_5N_3BrSNa$) ([M+Na]$^+$), 371.92602; found, 371.92548.

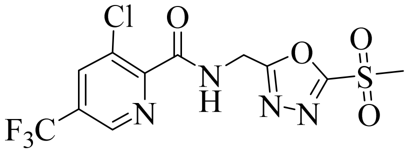

3-chloro-N-((5-(methylsulfonyl)-1,3,4-oxadiazol-2-yl)methyl)-5-(trifluoromethyl)picolinamide **(6)**. White solid, m.p. 88.6–89.5 °C, yield, 83%; ^{1}H NMR (400 MHz, DMSO-*d*6) *δ* 9.70 (t, *J* = 5.8 Hz, 1H, -CONH-), 9.04 (s, 1H, Ar-H), 8.67 (s, 1H, Ar-H), 4.89 (d, *J* = 5.8 Hz, 2H, -CONH$\underline{CH_2}$-), 3.69 (s, 3H, -CH_3). ^{13}C NMR (100 MHz, DMSO-*d*6) *δ* 166.54, 164.62, 162.97, 153.76, 144.58, 137.02, 129.75, 128.36, 128.03, 127.70, 124.29, 121.57, 43.15, 34.79. HRMS (ESI): calcd for ($C_{11}H_8ClF_3O_4N_4SNa$) ([M+Na]$^+$), 406.97991; found, 406.97913.

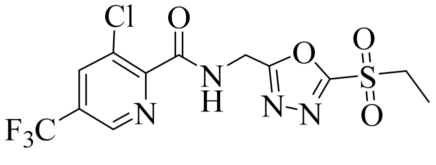

3-chloro-N-((5-(ethylsulfonyl)-1,3,4-oxadiazol-2-yl)methyl)-5-(trifluoromethyl)picolinamide **(7)**. White solid, m.p. 76.8–77.3 °C, yield, 78%; ^{1}H NMR (400 MHz, DMSO-*d*6) *δ* 9.70 (t, *J* = 5.8 Hz, 1H, -CONH-), 9.04 (s, 1H, Ar-H), 8.67 (s, 1H, Ar-H), 4.89 (d, *J* = 5.7 Hz, 2H, -CONH$\underline{CH_2}$-), 3.77 (q, *J* = 7.3 Hz, 2H, -SO_2CH_2-), 1.30 (t, *J* = 7.3 Hz, 3H, -CH_3). ^{13}C NMR (100 MHz, DMSO-*d*6) *δ* 166.95, 164.63, 161.85, 153.78, 144.59, 137.02, 129.76, 128.36, 128.03, 127.70, 124.28, 121.56, 49.85, 34.88, 7.17. HRMS (ESI): calcd for ($C_{12}H_{10}ClF_3O_4N_4SNa$) ([M+Na]$^+$), 420.99556; found, 420.99484.

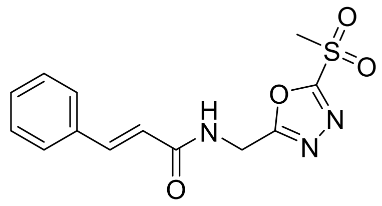

***N*-((5-(methylsulfonyl)-1,3,4-oxadiazol-2-yl)methyl)cinnamamide** **(8)**. White solid, m.p. 150.3–151.5 °C, yield, 86%; ^{1}H NMR (400 MHz, DMSO-d_6) δ 9.01 (t, *J* = 5.6 Hz, 1H, -CONH-), 7.62 (s, 1H, Ar-H), 7.60 (s, 1H, Ar-H), 7.52 (d, *J* = 15.9 Hz, 1H, Ar-H), 7.47 – 7.37 (m, 3H, Ar-H, -CH=CH-), 6.71 (d, *J* = 15.9 Hz, 1H, Ar-H), 4.78 (d, *J* = 5.7 Hz, 2H, -CONH$\underline{CH_2}$-), 3.69 (s, 3H, -SO_2CH_3). ^{13}C NMR (100 MHz, DMSO-d_6) δ 167.19, 165.98, 162.89, 140.69, 134.99, 130.31, 129.46, 128.22, 121.23, 43.18, 34.64. HRMS (ESI): calcd for ($C_{13}H_{13}O_4N_3SNa$) ([M+Na]$^+$), 330.05190; found, 330.05129.

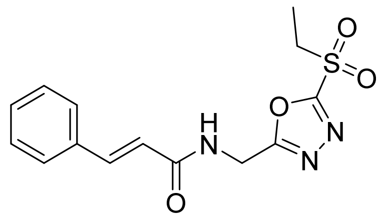

***N*-((5-(ethylsulfonyl)-1,3,4-oxadiazol-2-yl)methyl)cinnamamide** **(9)**. White solid, m.p. 117.1–117.9 °C, yield, 80%; ^{1}H NMR (400 MHz, $CDCl_3$) δ 7.69 (d, *J* = 15.6 Hz, 1H, -CONH-), 7.54 – 7.48 (m, 2H, -CH=CH-), 7.42 – 7.33 (m, 3H, Ar-H), 6.72 (t, *J* = 5.6 Hz, 1H, Ar-H), 6.53 (d, *J* = 15.6 Hz, 1H, Ar-H), 4.92 (d, *J* = 5.9 Hz, 2H, -CONH$\underline{CH_2}$-), 3.54 (q, *J* = 7.4 Hz, 2H, -SO_2CH_2-), 1.49 (t, *J* = 7.4 Hz, 3H, -CH_3). ^{13}C NMR (100 MHz, $CDCl_3$) δ 166.33, 166.26, 162.02, 143.12, 134.26, 130.27, 128.94, 128.08, 118.86, 50.11, 34.89, 6.74. HRMS (ESI): calcd for ($C_{14}H_{15}O_4N_3SNa$) ([M+Na]$^+$), 344.06755; found, 344.06702.

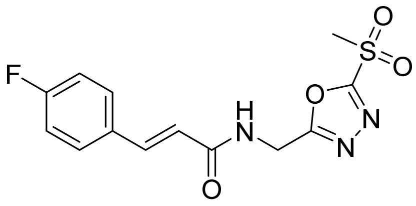

***3-(4-fluorophenyl)-N-((5-(methylsulfonyl)-1,3,4-oxadiazol-2-yl)methyl)acryl amide* (10)**. White solid, m.p. 147.5–148.9 °C, yield, 85%; ^{1}H NMR (400 MHz, DMSO-d_6) δ 8.98 (t, *J* = 5.7 Hz, 1H, -CONH-), 7.68 (dd, *J* = 8.6, 5.6 Hz, 2H, -CH=CH-), 7.51 (d, *J* = 15.9 Hz, 1H, Ar-H), 7.27 (t, *J* = 8.8 Hz, 2H, Ar-H), 6.65 (d, *J* = 15.9 Hz, 1H, Ar-H), 4.77 (d, *J* = 5.7 Hz, 2H, -CONH$\underline{CH_2}$-), 3.68 (s, 3H, -CH_3). ^{13}C NMR (100 MHz, DMSO-d_6) δ 167.18, 165.91, 162.89, 139.50, 131.66, 130.49, 121.13, 116.55, 116.34, 43.18, 34.64. HRMS (ESI): calcd for ($C_{13}H_{12}O_4N_3FSNa$) ($[M+Na]^+$), 348.04248; found, 348.04175.

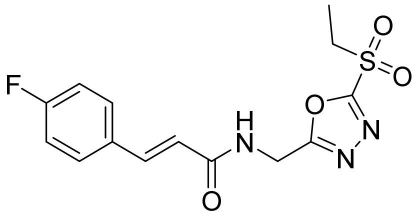

***N-((5-(ethylsulfonyl)-1,3,4-oxadiazol-2-yl)methyl)-3-(4-fluorophenyl)acryla mide* (11)**. White solid, m.p. 112.2–113.3 °C, yield, 82%; ^{1}H NMR (400 MHz, $CDCl_3$) δ 7.56 (d, *J* = 15.6 Hz, 1H, -CONH-), 7.46 – 7.36 (m, 2H, Ar-H), 7.00 – 6.96 (m, 2H, Ar-H), 6.74 (t, *J* = 5.7 Hz, 1H, Ar-H), 6.38 (d, *J* = 15.6 Hz, 1H, Ar-H), 4.85 (d, *J* = 5.9 Hz, 2H, -CONH$\underline{CH_2}$-), 3.47 (q, *J* = 7.4 Hz, 2H, -SO_2CH_2-), 1.42 (t, *J* = 7.4 Hz, 3H, -CH_3). ^{13}C NMR (100 MHz, $CDCl_3$) δ 166.39, 166.16, 162.02, 141.82, 129.99, 129.90, 118.59, 116.18, 115.96, 50.11, 34.89, 6.73. HRMS (ESI): calcd for ($C_{14}H_{14}O_4N_3FSNa$) ($[M+Na]^+$), 362.05813; found, 362.05719.

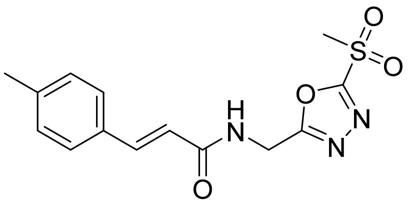

***N-((5-(methylsulfonyl)-1,3,4-oxadiazol-2-yl)methyl)-3-(p-tolyl)acrylamide* (12)**. White solid, m.p. 148.7–149.6 °C, yield, 85%; ^{1}H NMR (400 MHz, $CDCl_3$) *δ* 7.60 (d, *J* = 15.6 Hz, 1H, -CONH-), 7.35 (d, *J* = 7.9 Hz, 2H, Ar-H), 7.11 (d, *J* = 7.8 Hz, 2H, Ar-H), 6.43 – 6.32(m, 2H, -CH=CH-), 4.85 (d, *J* = 5.8 Hz, 2H, -CONH$\underline{CH_2}$-), 3.39 (s, 3H, $-SO_2CH_3$), 2.30 (s, 3H, Ph-CH_3). ^{13}C NMR (100 MHz, $CDCl_3$) *δ* 166.30, 166.11, 162.85, 143.25, 140.71, 131.47, 129.66, 128.04, 117.61, 42.98, 34.82, 21.48. HRMS (ESI): calcd for ($C_{14}H_{15}O_4N_3SNa$) ($[M+Na]^+$), 344.06755; found, 344.06696.

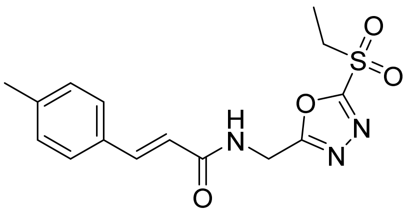

***N-((5-(ethylsulfonyl)-1,3,4-oxadiazol-2-yl)methyl)-3-(p-tolyl)acrylamide* (13)**. White solid, m.p. 112.2–113.7 °C, yield, 75%; ^{1}H NMR (400 MHz, $CDCl_3$) *δ* 7.59 (d, *J* = 15.6 Hz, 1H, -CONH-), 7.33 (d, *J* = 8.0 Hz, 2H, Ar-H), 7.10 (d, *J* = 7.9 Hz, 2H, -CH=CH-), 6.58 (t, *J* = 5.7 Hz, 1H, Ar-H), 6.40 (d, *J* = 15.6 Hz, 1H, Ar-H), 4.84 (d, *J* = 5.9 Hz, 2H, -CONH$\underline{CH_2}$-), 3.46 (q, *J* = 7.4 Hz, 2H, $-SO_2CH_2$-), 2.29 (s, 3H, Ph-CH_3), 1.41 (t, *J* = 7.4 Hz, 3H, $-SO_2CH_2\underline{CH_3}$). ^{13}C NMR (100 MHz, $CDCl_3$) *δ* 166.45, 166.37, 162.01, 143.10, 140.67, 131.52, 129.66, 128.07, 117.77, 50.11, 34.87, 21.50, 6.73. HRMS (ESI): calcd for ($C_{15}H_{17}O_4N_3SNa$) ($[M+Na]^+$), 358.08320; found, 358.08246.

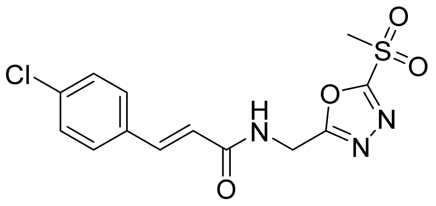

***3-(4-chlorophenyl)-N-((5-(methylsulfonyl)-1,3,4-oxadiazol-2-yl)methyl)acryl amide* (14)**. White solid, m.p. 162.4–163.8 °C, yield, 82%; ^{1}H NMR (400 MHz, DMSO-d_6) δ 9.08 (t, J = 5.4 Hz, 1H, -CONH-), 7.72 – 7.67(m, 2H, Ar-H), 7.61 – 7.52 (m, 3H, Ar-H, -CH=CH-), 6.78 (d, J = 15.8 Hz, 1H, Ar-H), 4.84 (d, J = 5.6 Hz, 2H, -CONH$\underline{CH_2}$-), 3.75 (s, 3H, -CH_3). ^{13}C NMR (100 MHz, DMSO-d_6) δ 167.14, 165.79, 162.88, 139.34, 134.72, 133.96, 129.95, 129.49, 122.01, 43.17, 34.65. HRMS (ESI): calcd for ($C_{13}H_{12}O_4N_3ClSNa$) ([M+Na]$^+$), 364.01293; found, 364.01221.

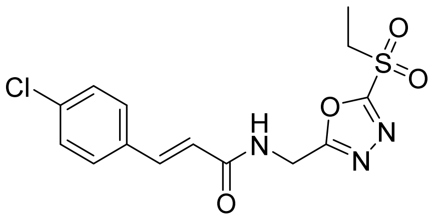

***3-(4-chlorophenyl)-N-((5-(ethylsulfonyl)-1,3,4-oxadiazol-2-yl)methyl)acryla mide* (15)**. White solid, m.p. 143.2–143.8 °C, yield, 80%; ^{1}H NMR (400 MHz, DMSO-d_6) δ 9.01 (t, J = 5.7 Hz, 1H, -CONH-), 7.64 (d, J = 8.5 Hz, 2H, Ar-H), 7.55 – 7.44 (m, 3H, Ar-H, -CH=CH-), 6.71 (d, J = 15.9 Hz, 1H, Ar-H), 4.78 (d, J = 5.7 Hz, 2H, -CONH$\underline{CH_2}$-), 3.76 (q, J = 7.3 Hz, 2H, -SO_2CH_2-), 1.29 (t, J = 7.3 Hz, 3H, -CH_3). ^{13}C NMR (100 MHz, DMSO-d_6) δ 167.56, 165.82, 161.75, 139.33, 134.72, 133.96, 129.95, 129.49, 121.99, 49.82, 34.76, 7.12. HRMS (ESI): calcd for ($C_{14}H_{14}O_4N_3ClSNa$) ([M+Na]$^+$), 378.02858; found, 378.02805.

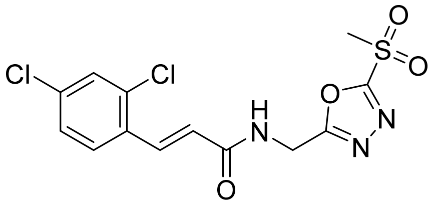

***3-(2,4-dichlorophenyl)-N-((5-(methylsulfonyl)-1,3,4-oxadiazol-2-yl)methyl)acrylamide* (16)**. White solid, m.p. 145.3–146.5 °C, yield, 78%; ^{1}H NMR (400 MHz, DMSO-*d*$_6$) *δ* 9.14 (t, *J* = 5.7 Hz, 1H, -CONH-), 7.84 – 7.69 (m, 3H, Ar-H, -CH=CH-), 7.52 (d, *J* = 8.5 Hz, 1H, Ar-H), 6.78 (d, *J* = 15.8 Hz, 1H, Ar-H), 4.79 (d, *J* = 5.7 Hz, 2H, -CONH<u>CH$_2$</u>-), 3.69 (s, 3H, -CH$_3$). ^{13}C NMR (100 MHz, DMSO-*d*$_6$) *δ* 167.02, 165.34, 162.90, 135.30, 134.72, 131.87, 129.95, 129.49, 128.58, 124.95, 43.18, 34.72. HRMS (ESI): calcd for ($C_{13}H_{11}O_4N_3Cl_2SNa$) ([M+Na]$^+$), 397.97395; found, 397.97336.

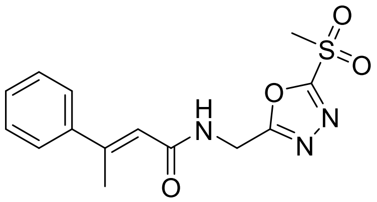

***N-((5-(methylsulfonyl)-1,3,4-oxadiazol-2-yl)methyl)-3-phenylbut-2-enamide* (17)**. White solid, m.p. 115.0–116.1 °C, yield, 89%; ^{1}H NMR (400 MHz, DMSO-*d*$_6$) *δ* 8.93 (t, *J* = 5.4 Hz, 1H, -CONH-), 7.46 – 7.42 (m, 4H, Ar-H, =<u>CH</u>-), 7.38 – 7.31 (m, 2H, Ar-H), 4.73 (d, *J* = 5.5 Hz, 2H, -CONH<u>CH$_2$</u>-), 3.69 (s, 3H, -SO$_2$CH$_3$), 2.05 (s, 3H, -CH$_3$). ^{13}C NMR (100 MHz, DMSO-*d*$_6$) *δ* 169.54, 167.34, 162.84, 136.17, 134.21, 131.67, 129.81, 128.96, 128.48, 43.18, 35.20, 14.64. HRMS (ESI): calcd for ($C_{14}H_{15}O_4N_3SNa$) ([M+Na]$^+$), 344.06755; found, 344.06714.

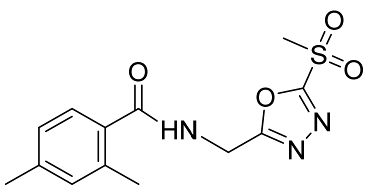

2,4-dimethyl-N-((5-(methylsulfonyl)-1,3,4-oxadiazol-2-yl)methyl)benzamide

(18). White solid, m.p. 113.5–114.8 °C, yield, 85%; ^{1}H NMR (400 MHz, DMSO-d_6) δ 9.04 (t, J = 5.5 Hz, 1H, -CONH-), 7.31 (d, J = 7.5 Hz, 1H, Ar-H), 7.09 (s, 1H, Ar-H), 7.07 (s, 1H, Ar-H), 4.77 (d, J = 5.6 Hz, 2H, -CONH$\underline{CH_2}$-), 3.68 (s, 3H, -SO_2CH_3), 2.32 (s, 3H, Ph-CH_3), 2.30 (s, 3H, Ph-CH_3). ^{13}C NMR (100 MHz, DMSO-d_6) δ 169.96, 167.31, 162.83, 139.99, 136.27, 133.11, 131.81, 127.81, 126.57, 43.15, 34.95, 21.27, 19.93. HRMS (ESI): calcd for ($C_{13}H_{15}O_4N_3SNa$) ($[M+Na]^+$), 332.06755; found, 332.06696.

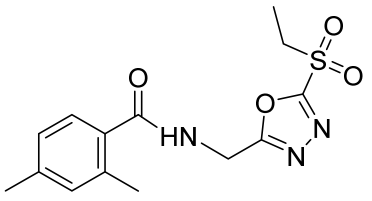

N-((5-(ethylsulfonyl)-1,3,4-oxadiazol-2-yl)methyl)-2,4-dimethylbenzamide

(19). White solid, m.p. 77.8–78.5 °C, yield, 82%; ^{1}H NMR (400 MHz, DMSO-d_6) δ 9.03 (t, J = 5.5 Hz, 1H, -CONH-), 7.31 (d, J = 7.5 Hz, 1H, Ar-H), 7.09 (s, 1H, Ar-H), 7.07 (s, 1H, Ar-H), 4.78 (d, J = 5.6 Hz, 2H, -CONH$\underline{CH_2}$-), 3.76 (q, J = 7.3 Hz, 2H, -SO_2CH_2-), 2.32 (s, 3H, Ph-CH_3), 2.30 (s, 3H, Ph-CH_3), 1.29 (t, J = 7.3 Hz, 3H, -$SO_2CH_2\underline{CH_3}$). ^{13}C NMR (100 MHz, DMSO-d_6) δ 169.98, 167.72, 161.71, 140.00, 136.24, 133.09, 131.81, 127.79, 126.58, 49.83, 35.04, 21.26, 19.91, 7.17. HRMS (ESI): calcd for ($C_{14}H_{17}O_4N_3SNa$) ($[M+Na]^+$), 346.08320; found, 346.08264.

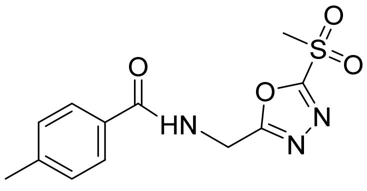

***4-methyl-N-((5-(methylsulfonyl)-1,3,4-oxadiazol-2-yl)methyl)benzamide* (20)**. White solid, m.p. 162.1–162.9 °C, yield, 52%; ^{1}H NMR (400 MHz, DMSO-d_6) δ 9.28 (t, J = 5.3 Hz, 1H, -CONH-), 7.80 (d, J = 8.1 Hz, 2H, Ar-H), 7.31 (d, J = 8.0 Hz, 2H, Ar-H), 4.81 (d, J = 5.5 Hz, 2H, -CONH$\underline{CH_2}$-), 3.68 (s, 3H, -SO_2CH_3), 2.37 (s, 3H, Ph-CH_3). ^{13}C NMR (100 MHz, DMSO-d_6) δ 167.32, 166.97, 162.86, 142.34, 130.79, 129.50, 127.88, 43.16, 35.12, 21.47. HRMS (ESI): calcd for ($C_{12}H_{13}O_4N_3SNa$) ([M+Na]$^+$), 318.05190; found, 318.05127.

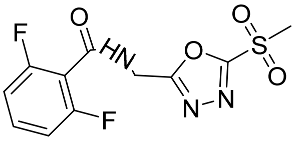

***2,6-difluoro-N-((5-(methylsulfonyl)-1,3,4-oxadiazol-2-yl)methyl)benzamide* (21)**. White solid, m.p. 136.8–137.6 °C, yield, 64%; ^{1}H NMR (400 MHz, DMSO-d_6) δ 9.65 (t, J = 5.5 Hz, 1H, -CONH-), 7.65 – 7.49 (m, 1H, Ar-H), 7.22 (t, J = 8.1 Hz, 2H, Ar-H), 4.86 (d, J = 5.7 Hz, 2H, -CONH$\underline{CH_2}$-), 3.69 (s, 3H, -SO_2CH_3). ^{13}C NMR (100 MHz, DMSO-d_6) δ 166.57, 162.95, 160.89, 158.09, 132.77, 112.66, 112.41, 43.12, 34.91. HRMS (ESI): calcd for ($C_{11}H_9O_4N_3F_2SNa$) ([M+Na]$^+$), 340.01740; found, 340.01675.

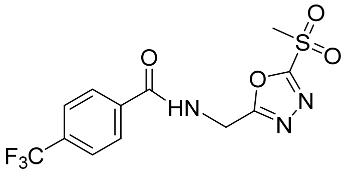

N-((5-(methylsulfonyl)-1,3,4-oxadiazol-2-yl)methyl)-4-(trifluoromethyl)benz

amide **(22)**. White solid, m.p. 136.2–136.8 °C, yield, 83%; ^{1}H NMR (400 MHz, DMSO-d_6) δ 9.62 (t, J = 5.5 Hz, 1H, -CONH-), 8.10 (s, 1H, Ar-H), 8.08 (s, 1H, Ar-H), 7.92 (s, 1H, Ar-H), 7.90 (s, 1H, Ar-H), 4.86 (d, J = 5.5 Hz, 2H, -CONHCH$_2$-), 3.68 (s, 3H, -CH$_3$). ^{13}C NMR (100 MHz, DMSO-d_6) δ 166.95, 165.99, 162.94, 137.31, 132.28, 131.96, 128.80, 126.07, 43.17, 35.21. HRMS (ESI): calcd for ($C_{12}H_{10}O_4N_3F_3SNa$) ([M+Na]$^+$), 372.02363; found, 372.02301.

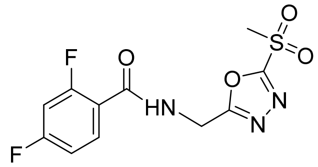

2,4-difluoro-N-((5-(methylsulfonyl)-1,3,4-oxadiazol-2-yl)methyl)benzamide **(23)**. White solid, m.p. 117.2–118.8 °C, yield, 72%; ^{1}H NMR (400 MHz, DMSO-d_6) δ 9.18 (s, 1H, -CONH-), 7.78 (dd, J = 15.3, 8.5 Hz, 1H, Ar-H), 7.43 (t, J = 9.0 Hz, 1H, Ar-H), 7.23 (t, J = 7.4 Hz, 1H, Ar-H), 4.83 (d, J = 5.6 Hz, 2H, -CONHCH$_2$-), 3.69 (s, 3H, -CH$_3$). ^{13}C NMR (100 MHz, DMSO-d_6) δ 166.91, 163.73, 162.88, 132.69, 112.69, 112.47, 105.58, 105.31, 105.05, 43.16, 35.22. HRMS (ESI): calcd for ($C_{11}H_9O_4N_3F_2SNa$) ([M+Na]$^+$), 340.01740; found, 340.01691.

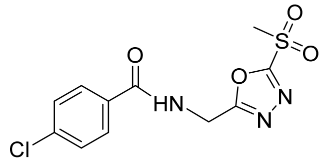

4-chloro-N-((5-(methylsulfonyl)-1,3,4-oxadiazol-2-yl)methyl)benzamide **(24)**. White solid, m.p. 166.2–167.0 °C, yield, 69%; ^{1}H NMR (400 MHz, DMSO-d_6) δ 9.46 (t, J = 5.4 Hz, 1H, -CONH-), 7.93 (s, 1H, Ar-H), 7.90 (s, 1H, Ar-H), 7.61 (s, 1H, Ar-H), 7.59 (s, 1H, Ar-H), 4.83 (d, J = 5.5 Hz, 2H, -CONHCH$_2$-), 3.68 (s, 3H, -CH$_3$). ^{13}C NMR (100 MHz, DMSO-d_6) δ 167.08, 166.10, 162.91, 137.19, 132.32,

129.80, 129.12, 43.17, 35.17. HRMS (ESI): calcd for ($C_{11}H_{10}O_4N_3ClSNa$) ([M+Na]$^+$), 337.99728; found, 337.99661.

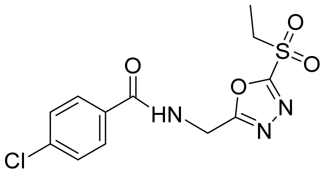

4-chloro-N-((5-(ethylsulfonyl)-1,3,4-oxadiazol-2-yl)methyl)benzamide **(25)**. White solid, m.p. 113.2–113.9 °C, yield, 66%; ^{1}H NMR (400 MHz, DMSO-d_6) δ 9.45 (t, J = 5.0 Hz, 1H, -CONH-), 7.92 (s, 1H, Ar-H), 7.90 (s, 1H, Ar-H), 7.61 (s, 1H, Ar-H), 7.59 (s, 1H, Ar-H), 4.83 (d, J = 5.4 Hz, 2H, -CONH$\underline{CH_2}$-), 3.76 (q, J = 7.3 Hz, 2H, -$\underline{CH_2}CH_3$), 1.28 (t, J = 7.3 Hz, 3H, -$CH_2\underline{CH_3}$). ^{13}C NMR (100 MHz, DMSO-d_6) δ 167.50, 166.13, 161.78, 137.19, 132.32, 129.78, 129.13, 49.83, 35.26, 7.10. HRMS (ESI): calcd for ($C_{12}H_{12}O_4N_3ClSNa$) ([M+Na]$^+$), 352.01293; found, 352.01254.

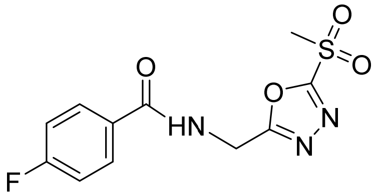

4-fluoro-N-((5-(methylsulfonyl)-1,3,4-oxadiazol-2-yl)methyl)benzamide **(26).** White solid, m.p. 135.0–135.8 °C, yield, 83%; ^{1}H NMR (400 MHz, DMSO-d_6) δ 9.40 (t, J = 5.4 Hz, 1H, -CONH-), 7.97 (dd, J = 8.8, 5.5 Hz, 2H, Ar-H), 7.35 (t, J = 8.8 Hz, 2H, Ar-H), 4.83 (d, J = 5.5 Hz, 2H, -CH_2-), 3.68 (s, 3H, -CH_3). ^{13}C NMR (100 MHz, DMSO-d_6) δ 167.17, 166.07, 162.90, 130.64, 130.55, 116.10, 115.88, 43.17, 35.17. HRMS (ESI): calcd for ($C_{11}H_{10}O_4N_3FSNa$) ([M+Na]$^+$), 322.02683; found, 322.02631.

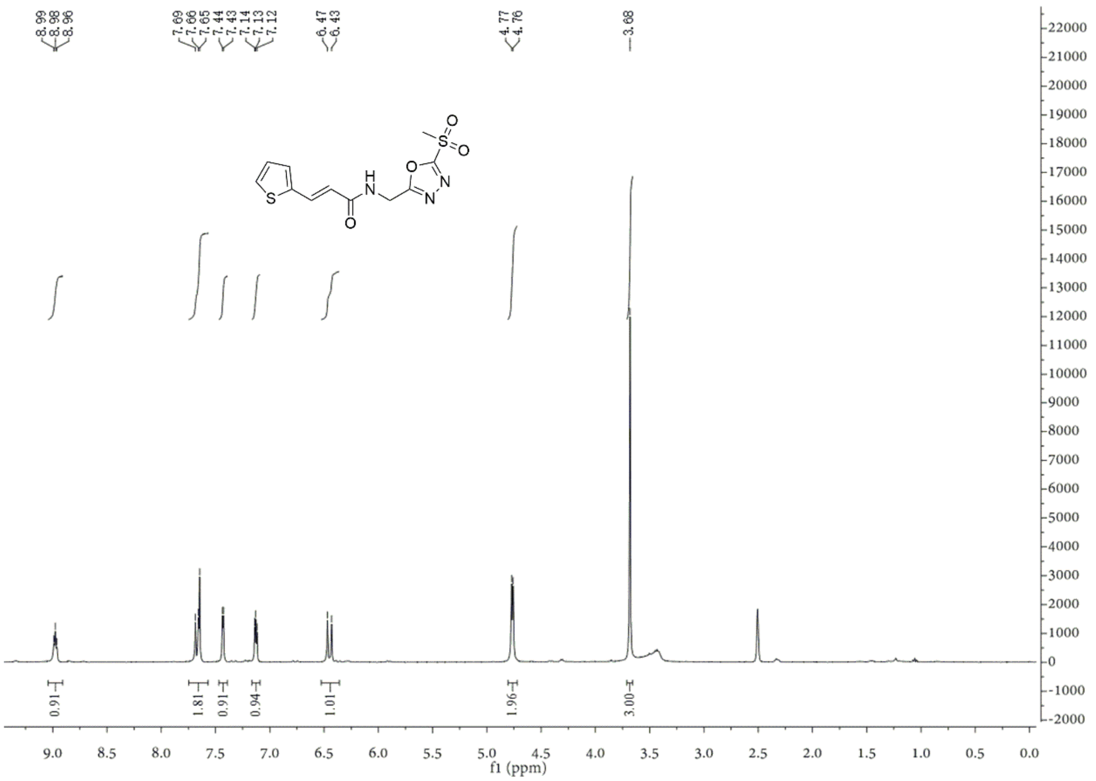

^{1}H NMR for compound **1**

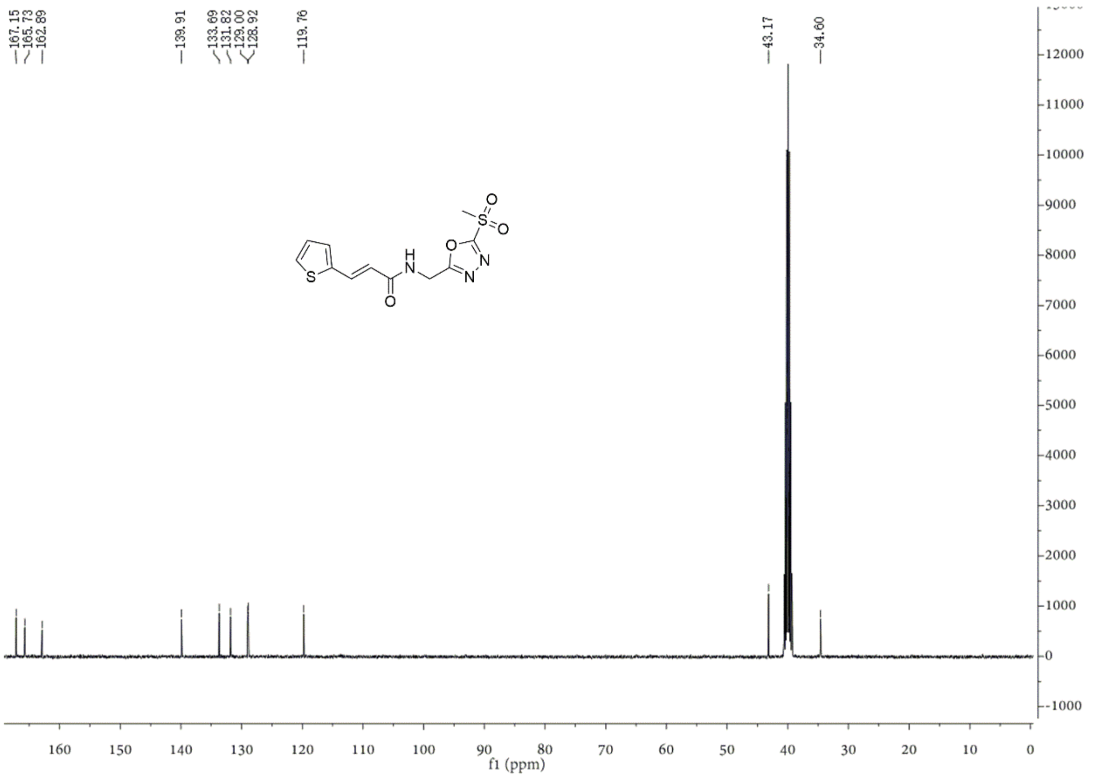

^{13}C NMR for compound **1**

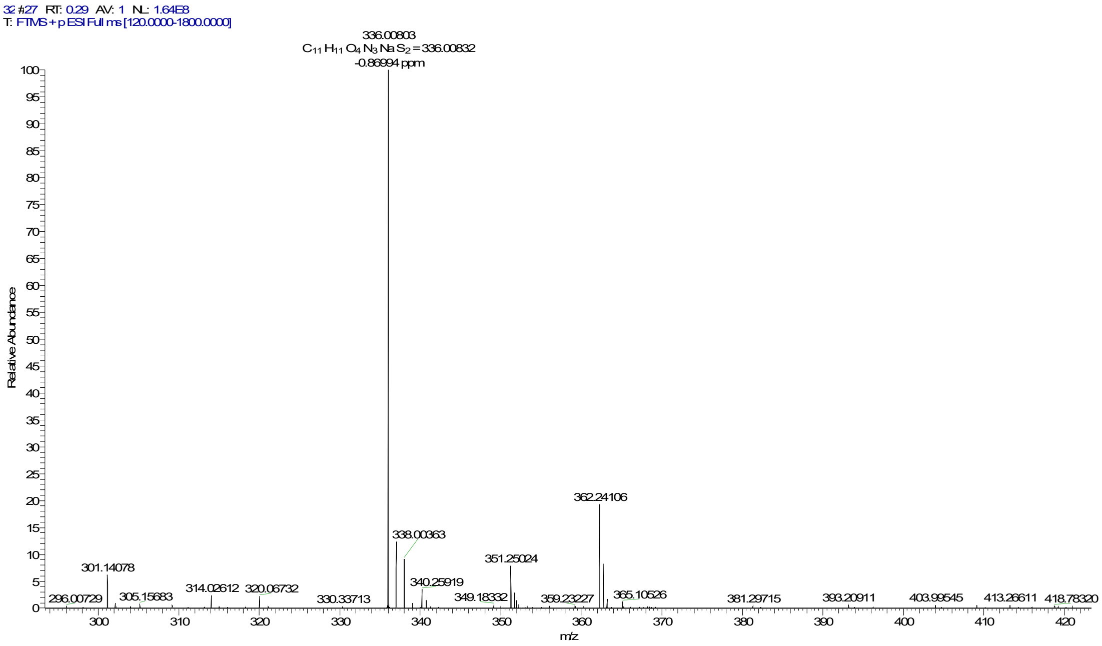

HRMS for compound **1**

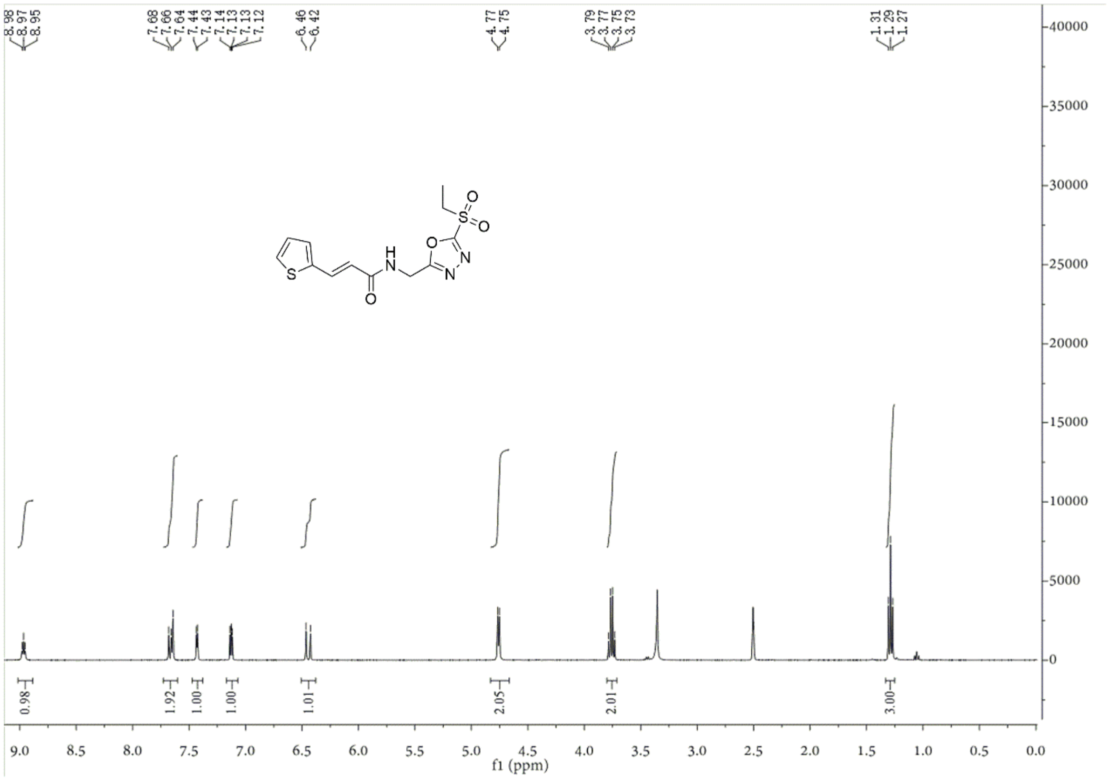

^{1}H NMR for compound **2**

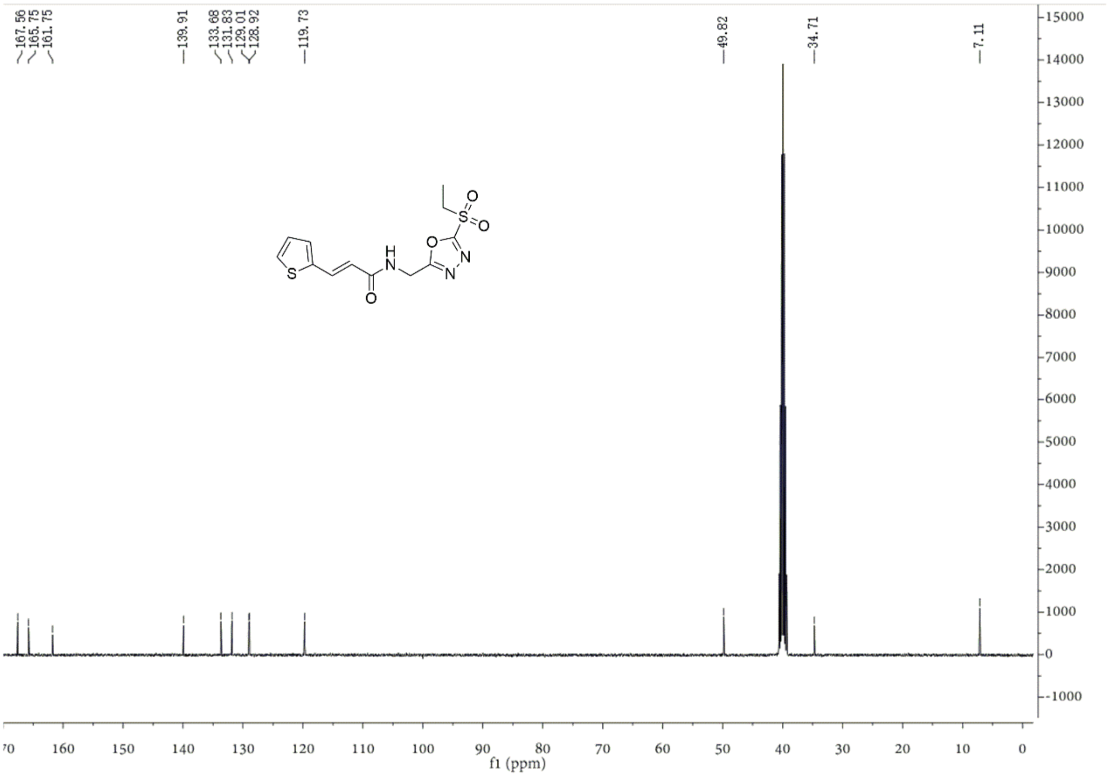

^{13}C NMR for compound **2**

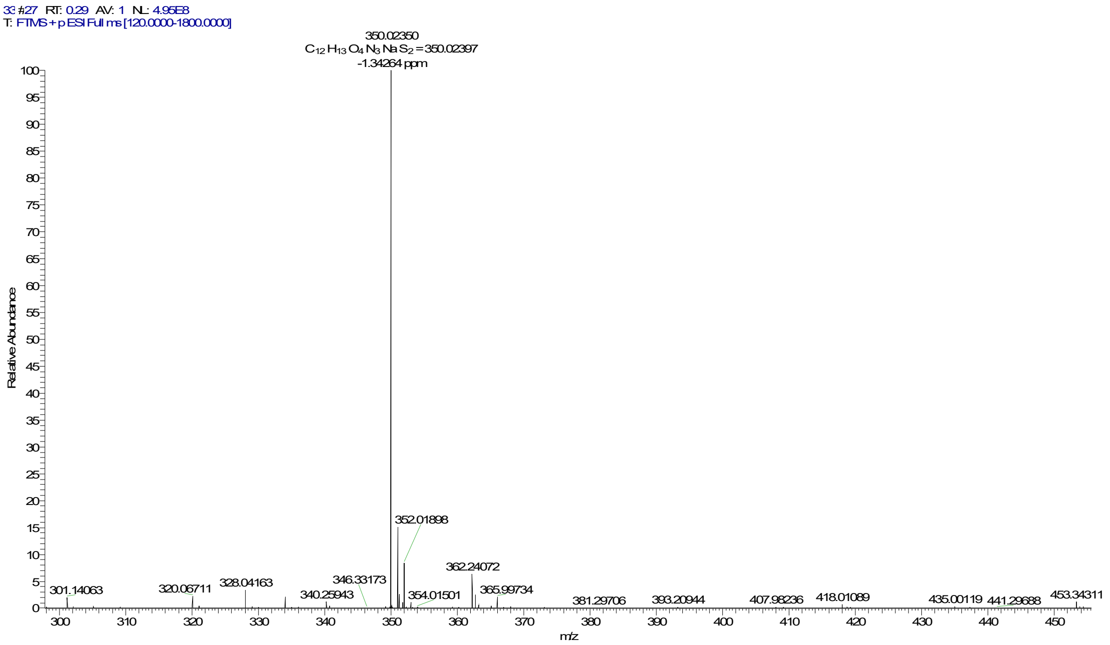

HRMS for compound **2**

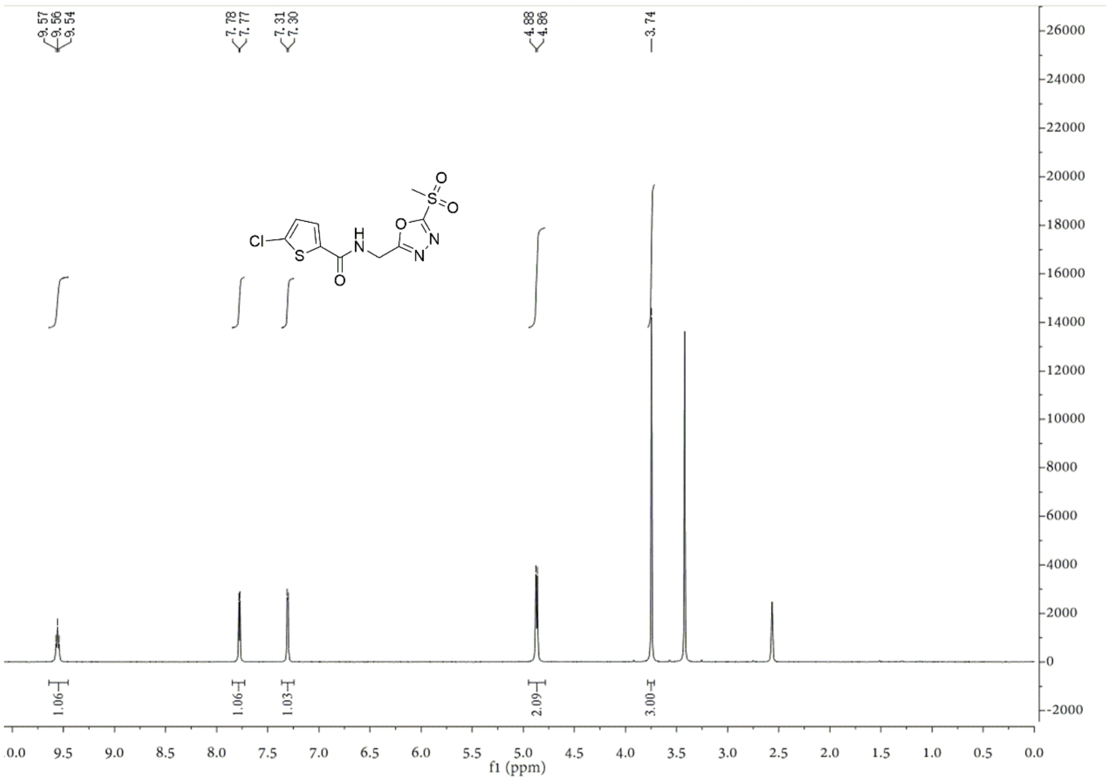

^{1}H NMR for compound **3**

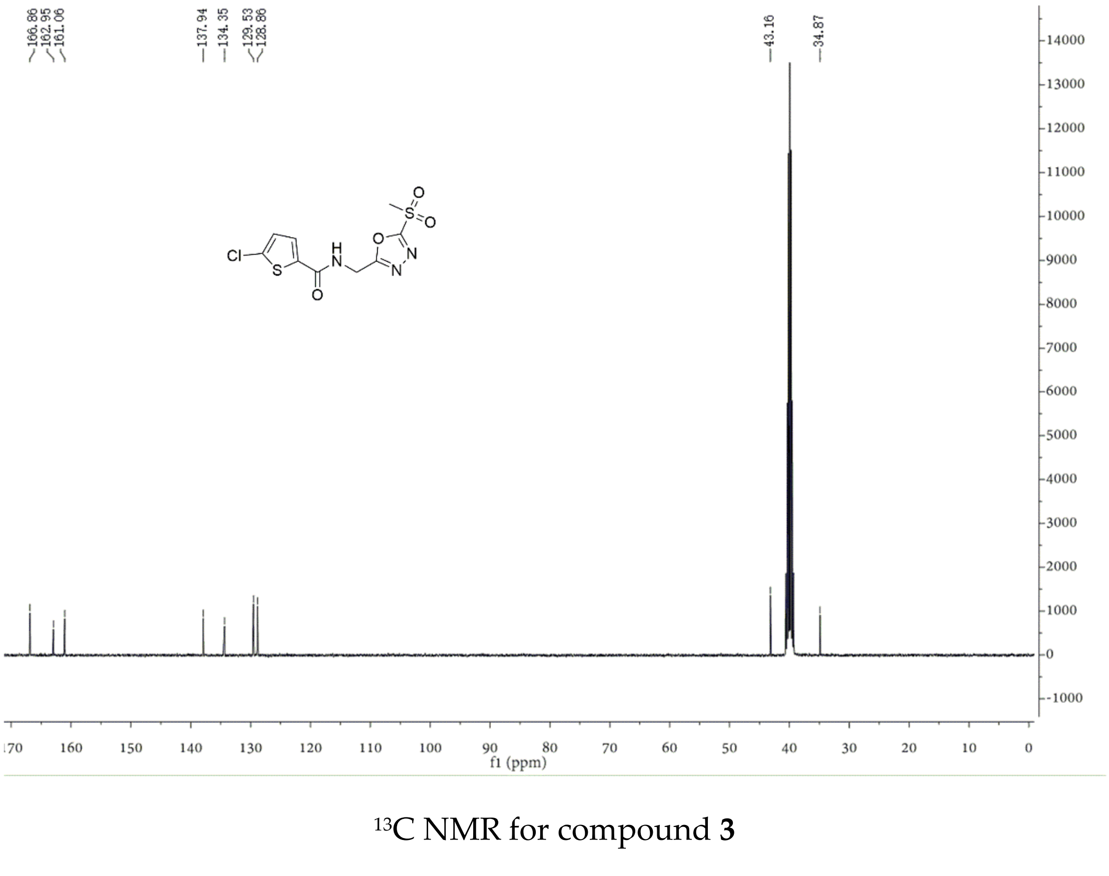

^{13}C NMR for compound **3**

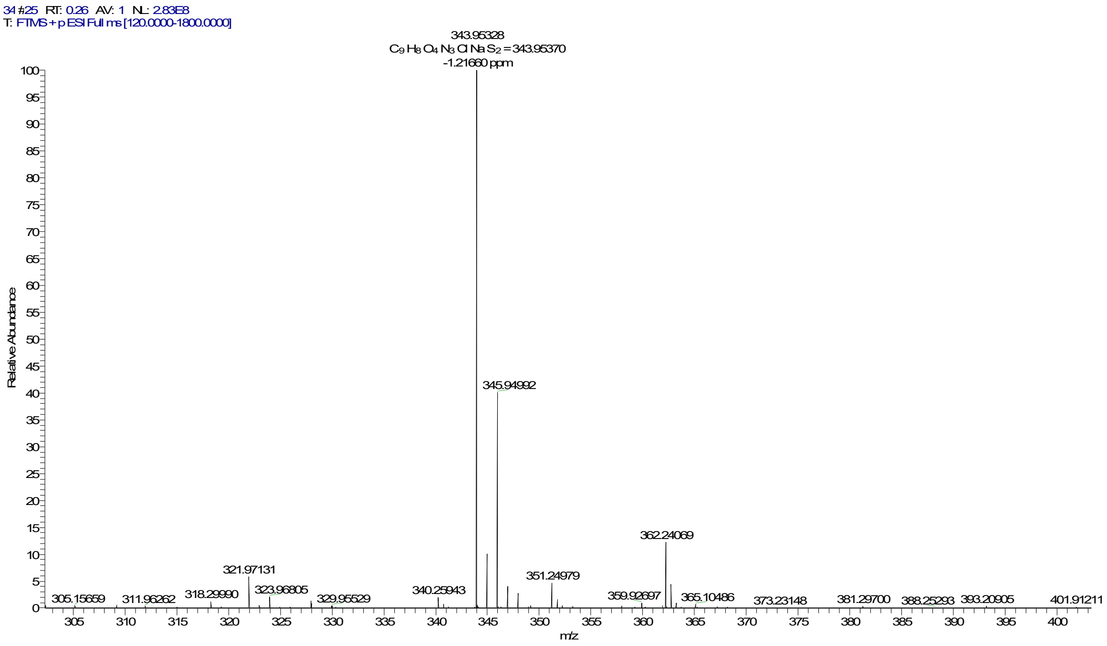

HRMS for compound **3**

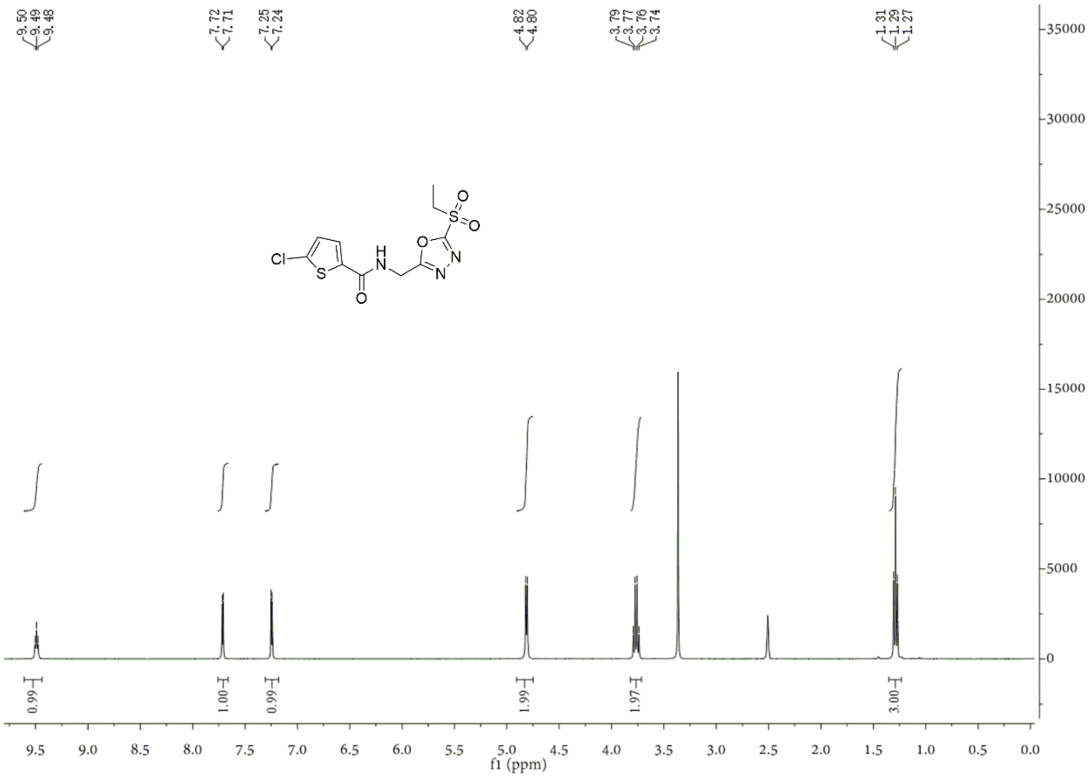

^{1}H NMR for compound **4**

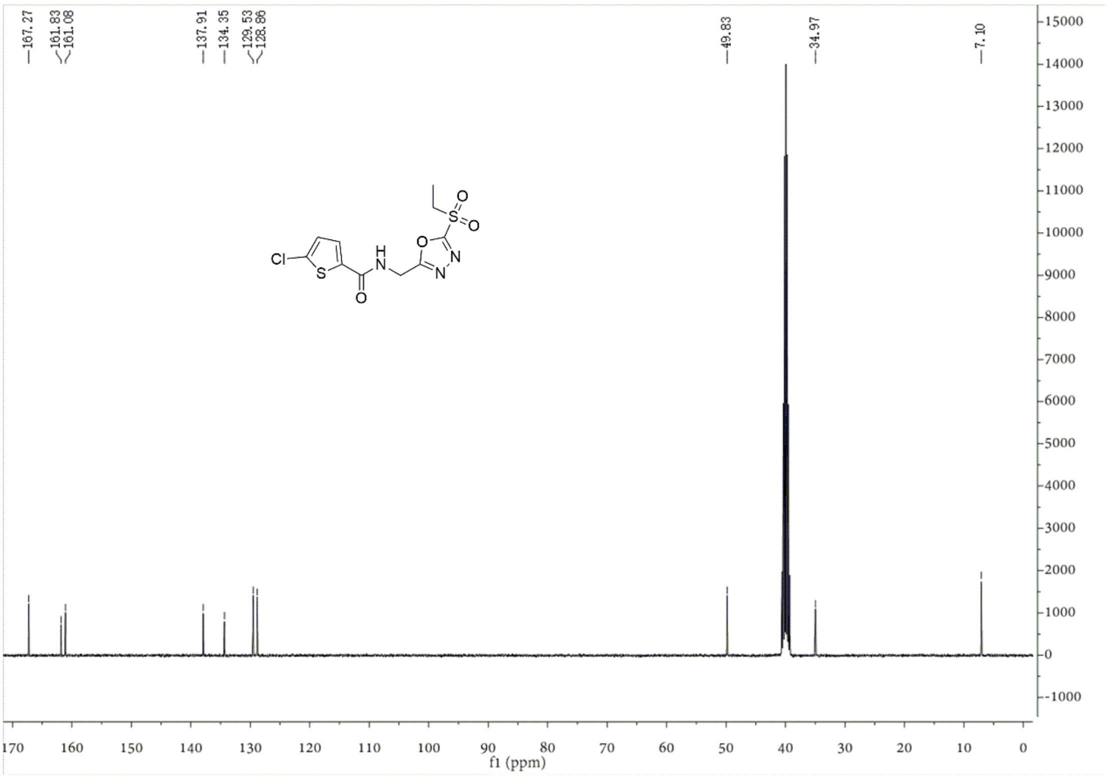

^{13}C NMR for compound **4**

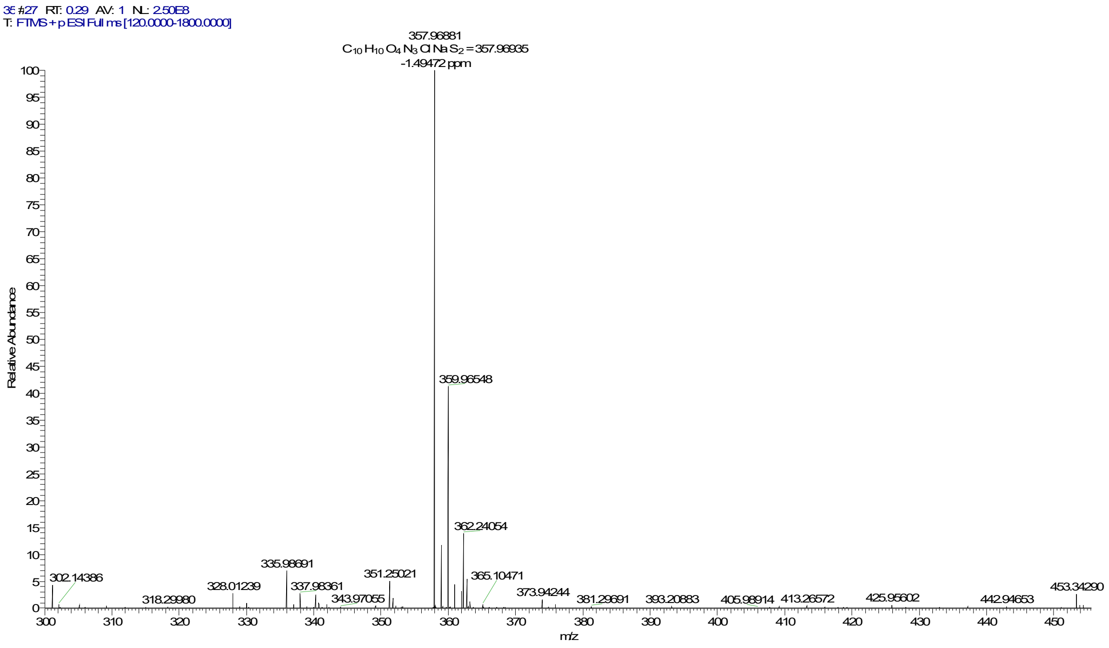

HRMS for compound **4**

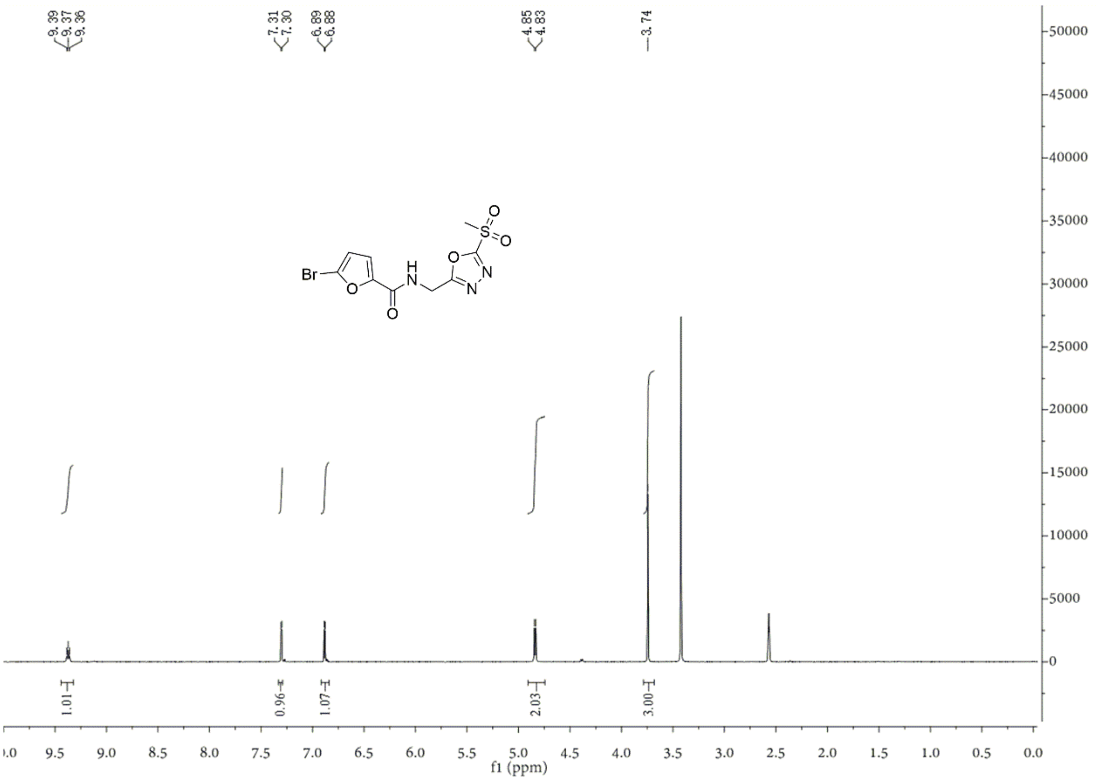

^{1}H NMR for compound **5**

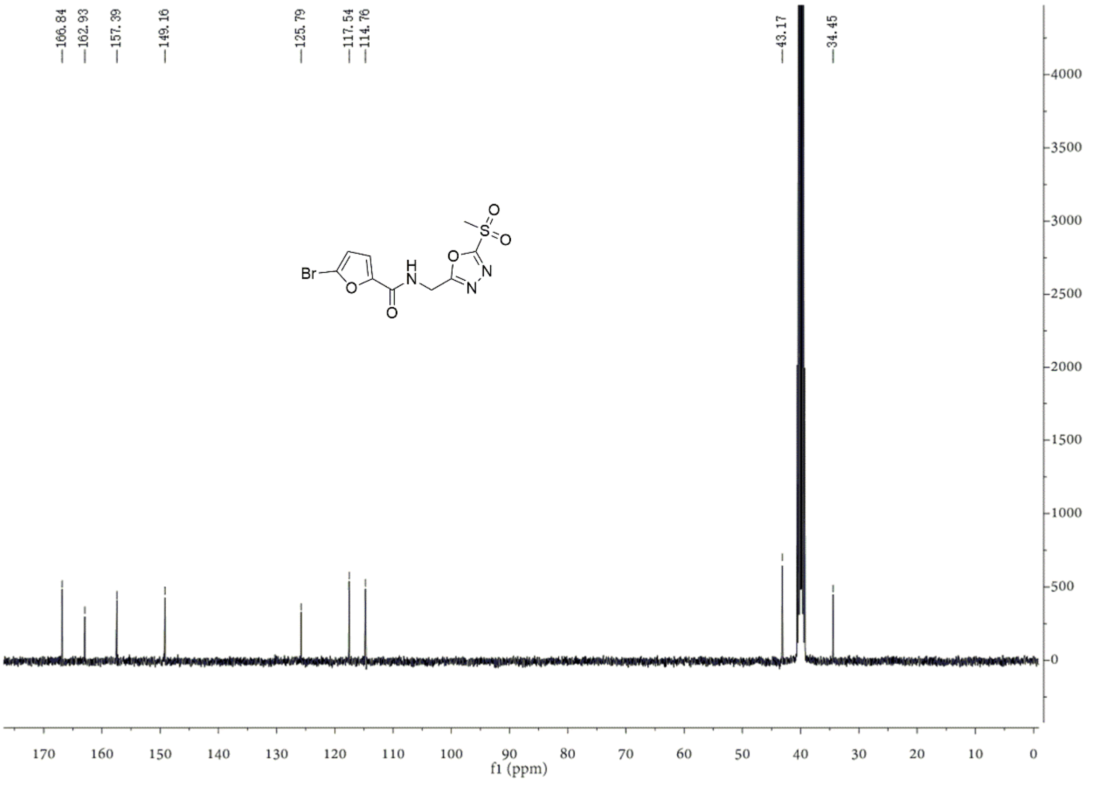

^{13}C NMR for compound **5**

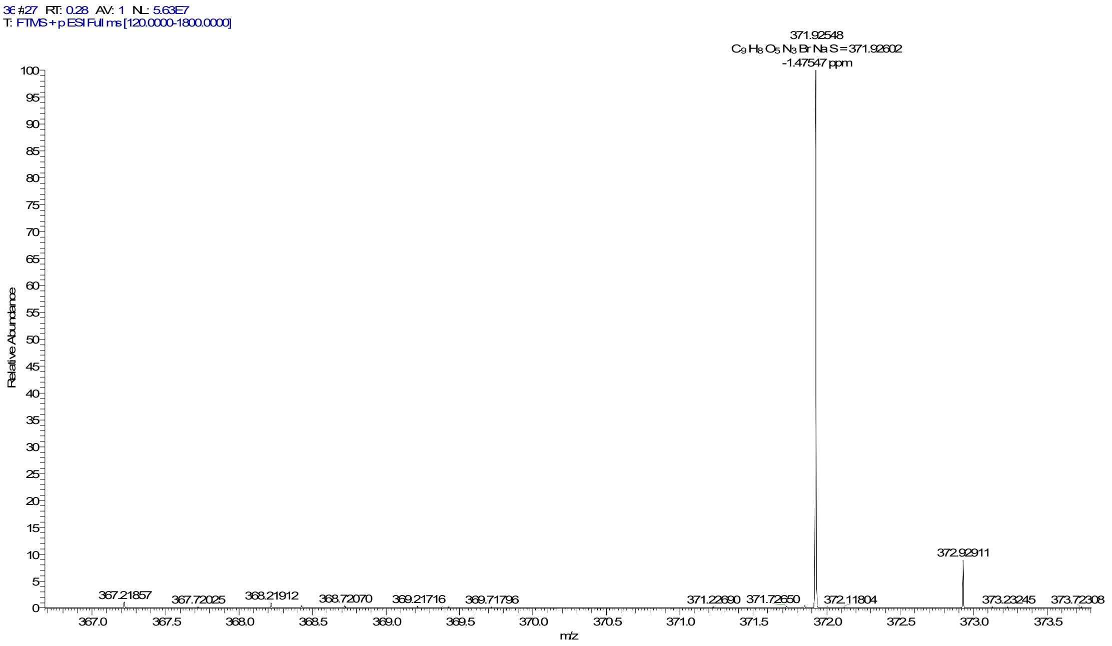

HRMS for compound **5**

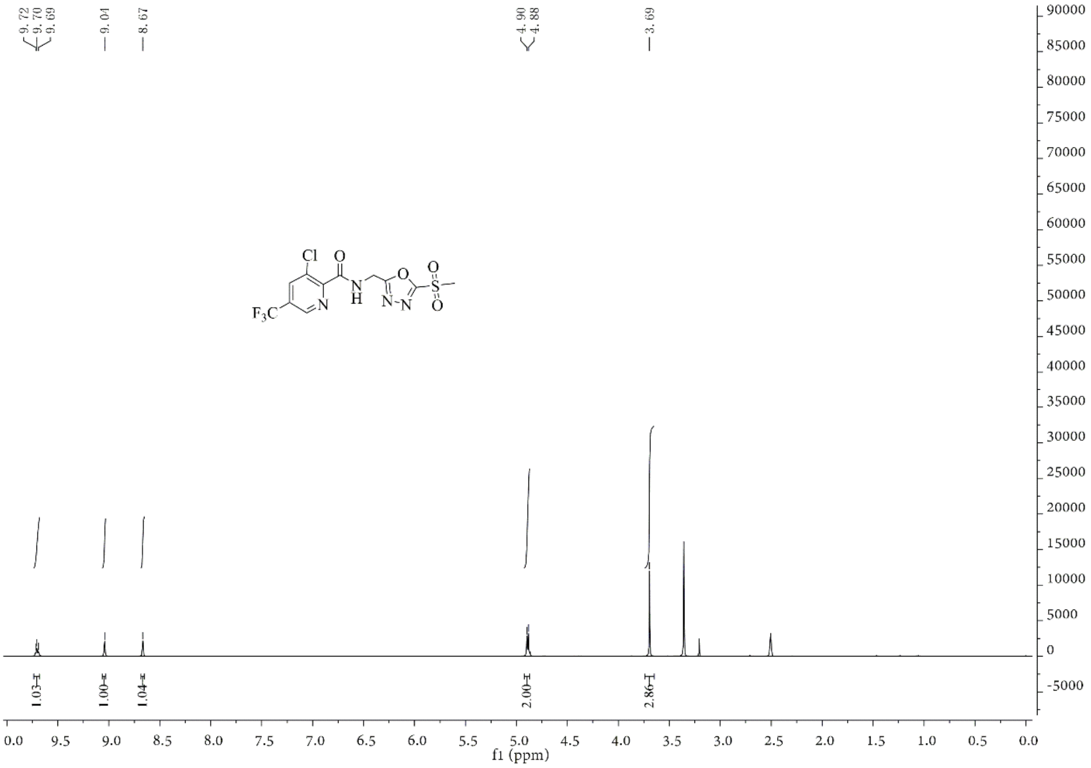

^{1}H NMR for compound **6**

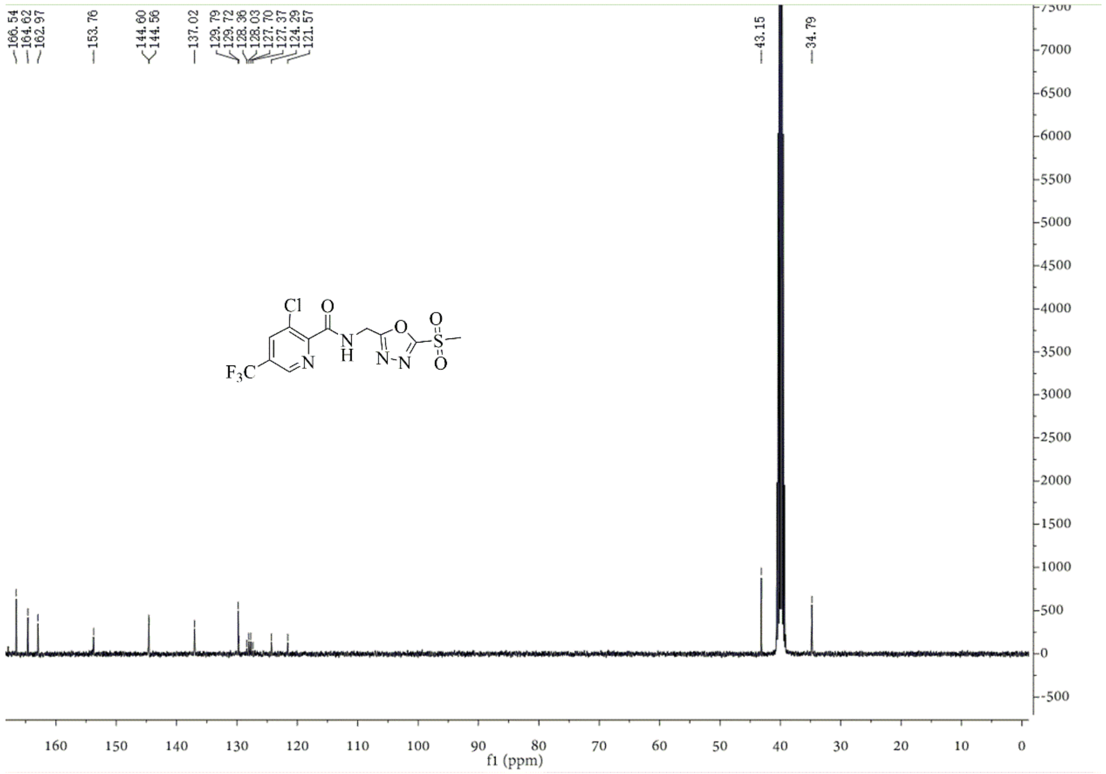

^{13}C NMR for compound **6**

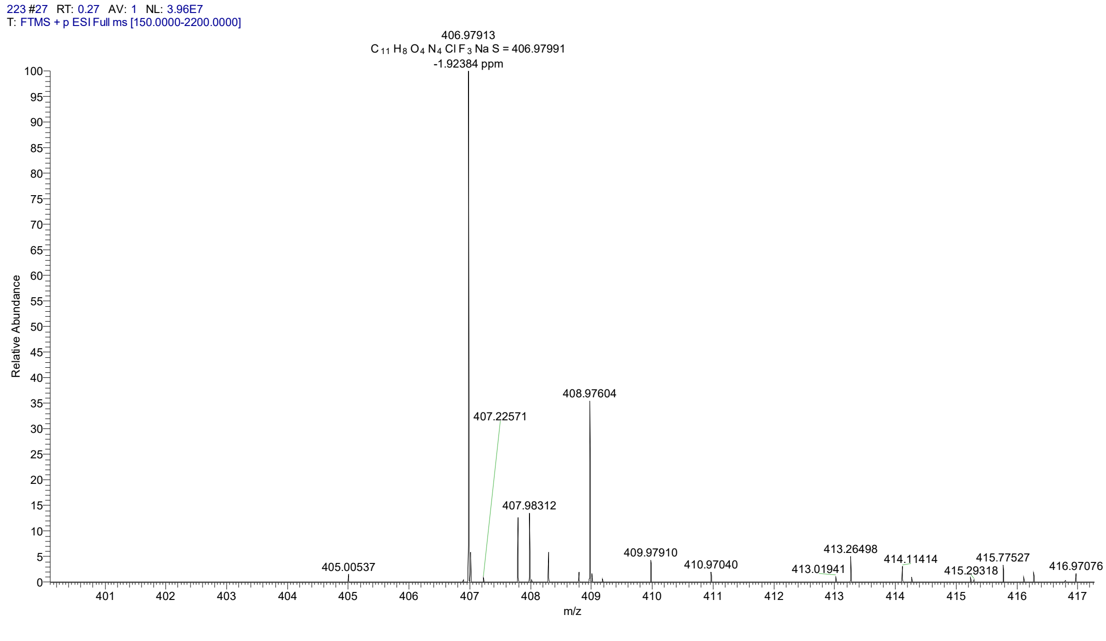

HRMS for compound **6**

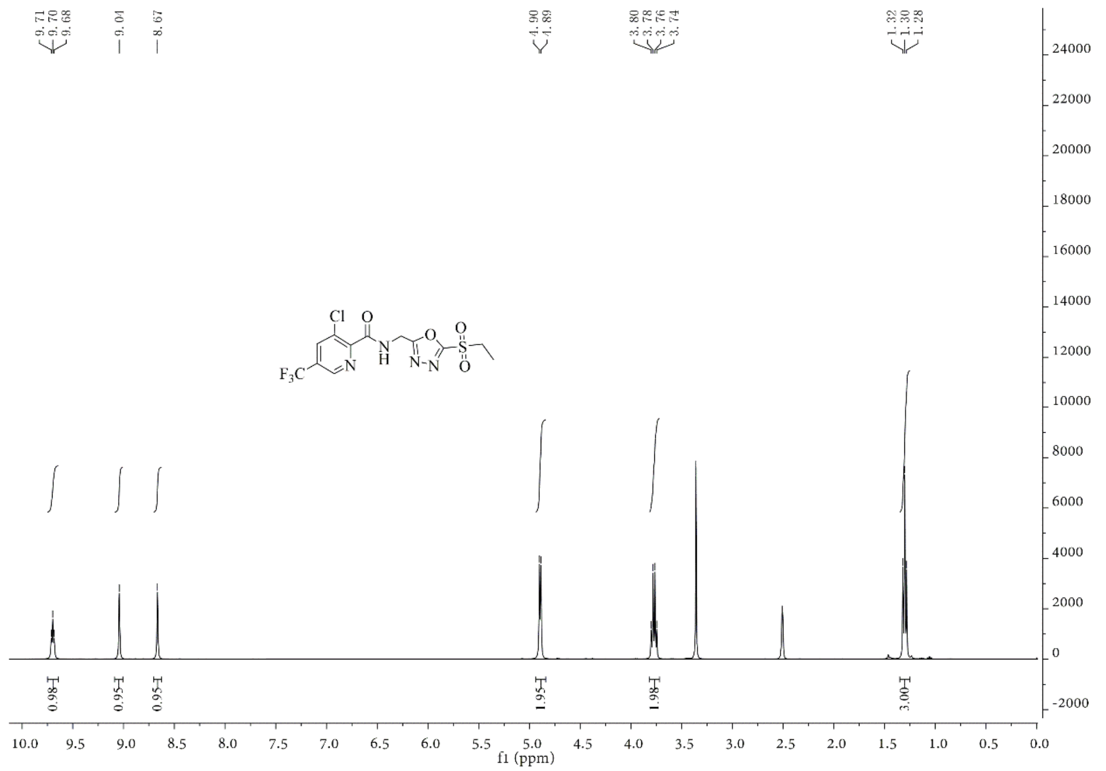

^{1}H NMR for compound **7**

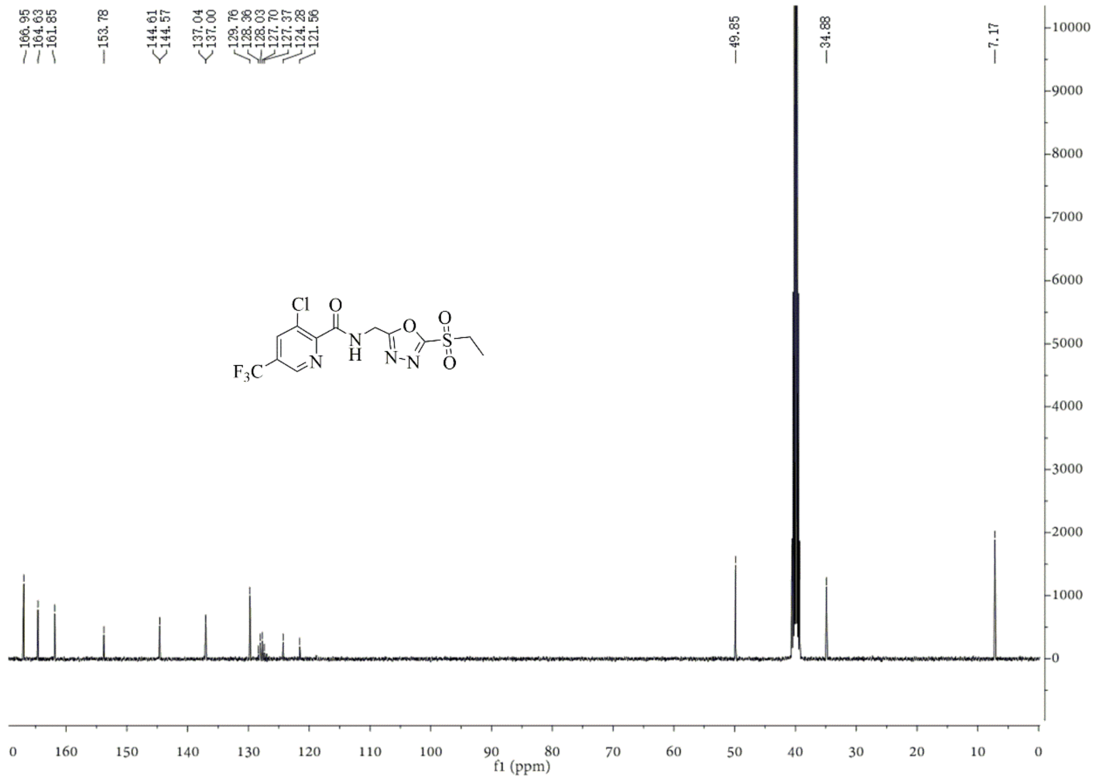

^{13}C NMR for compound **7**

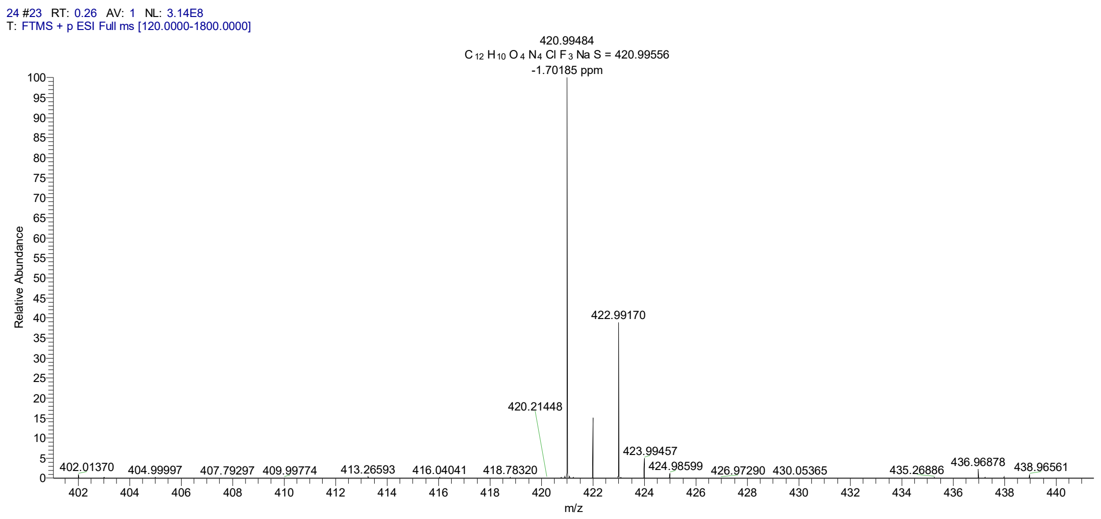

HRMS for compound **7**

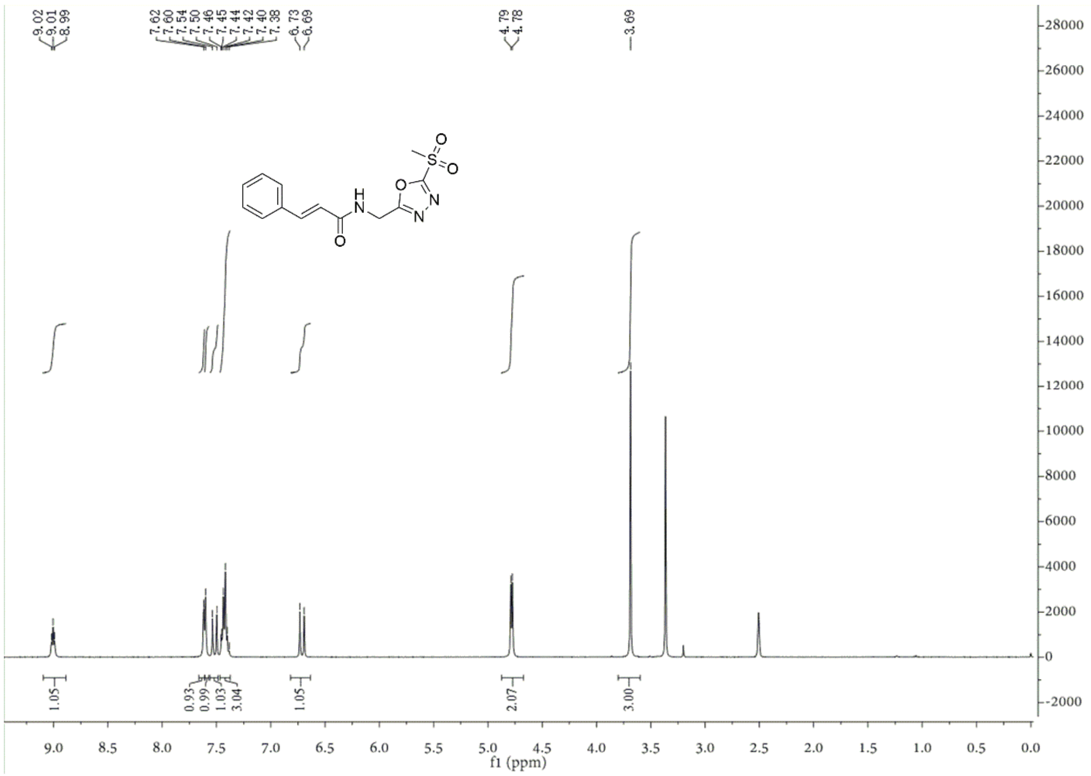

^{1}H NMR for compound **8**

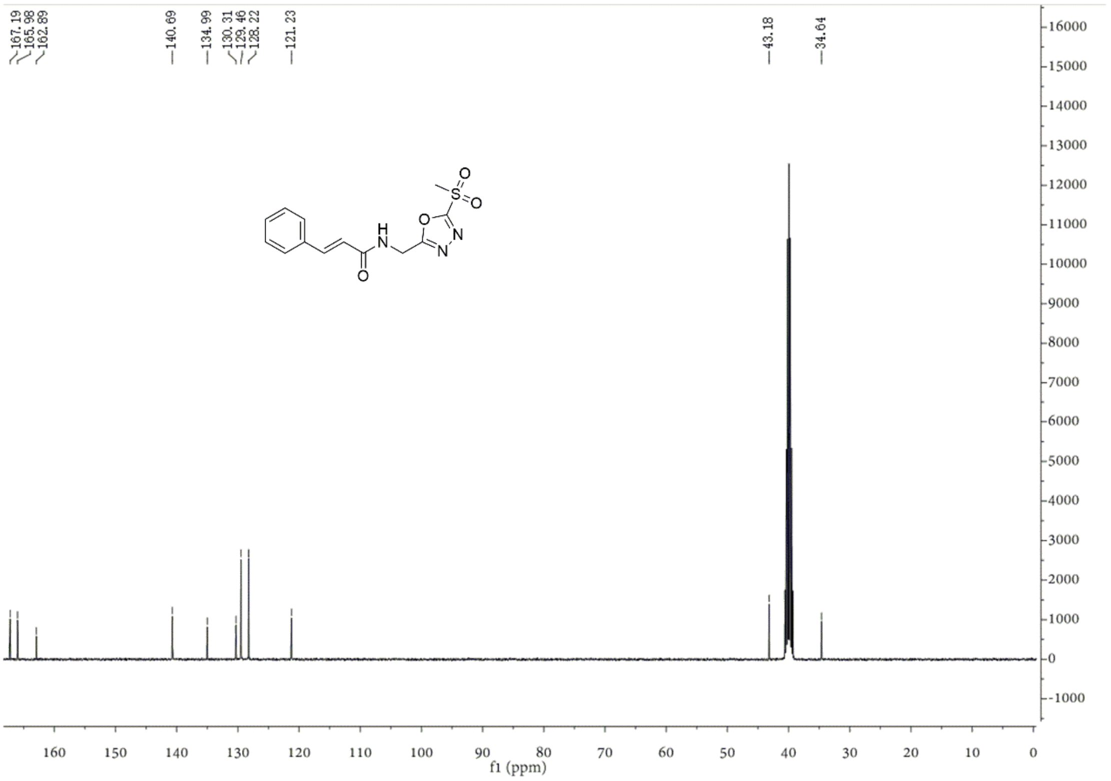

^{13}C NMR for compound **8**

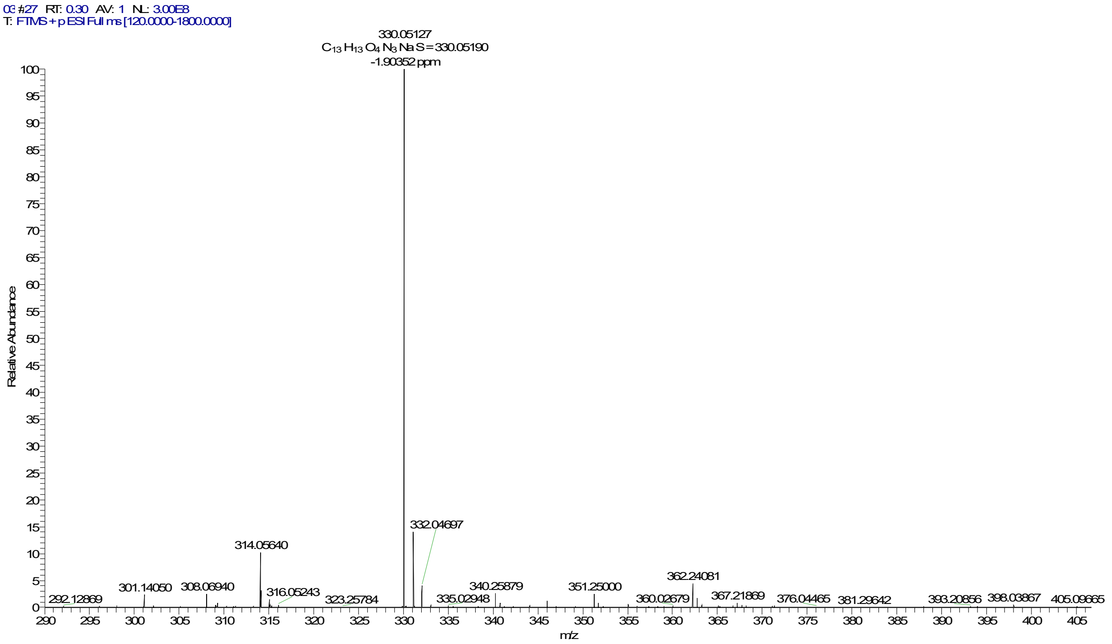

HRMS for compound **8**

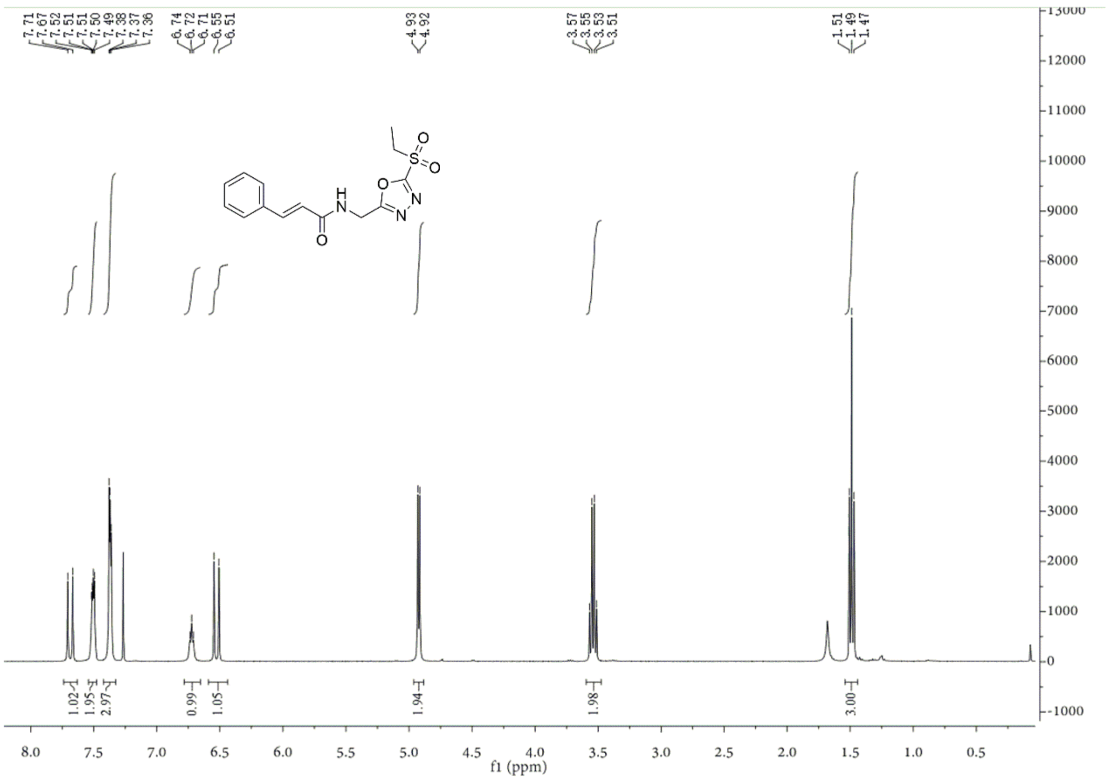

^{1}H NMR for compound **9**

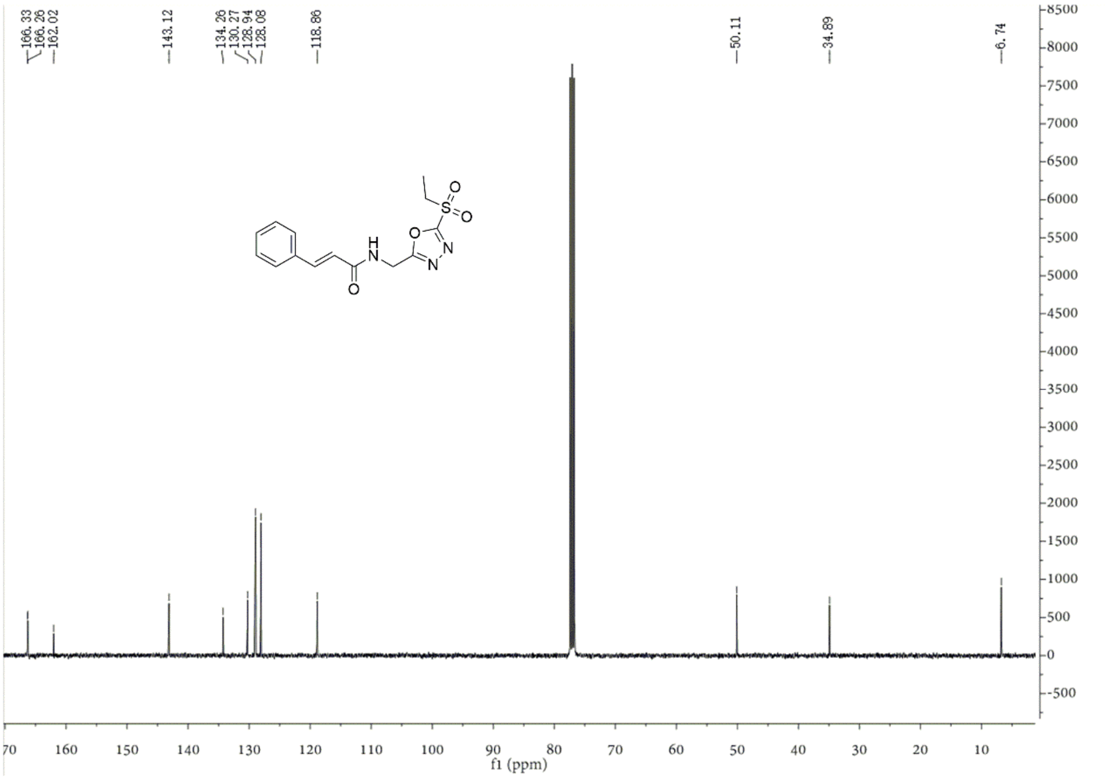

^{13}C NMR for compound **9**

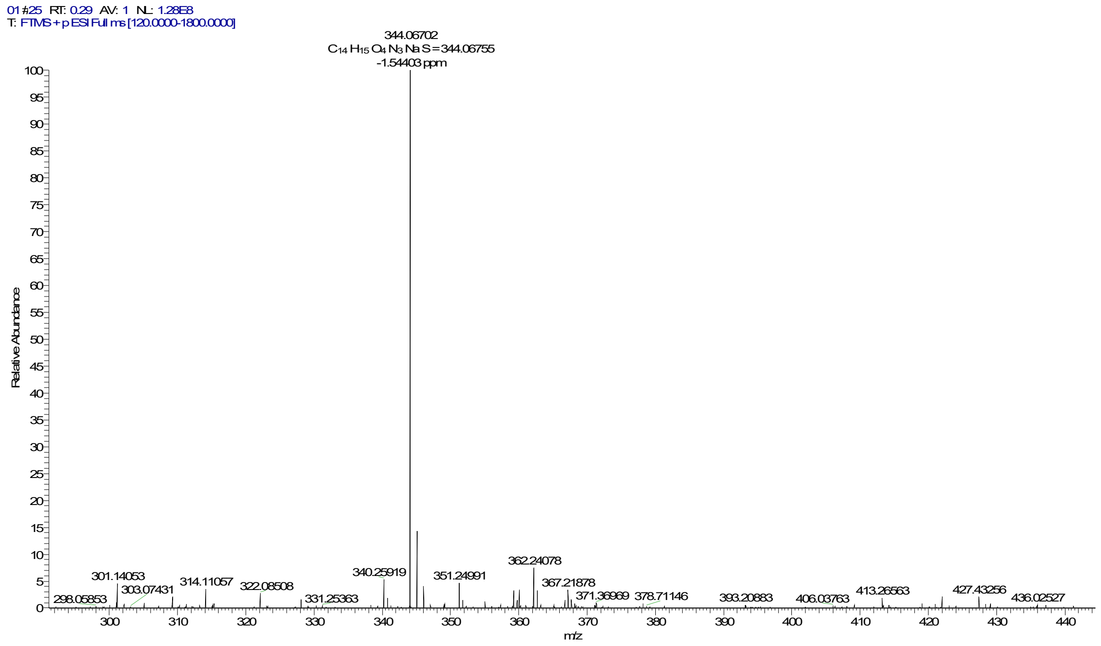

HRMS for compound **9**

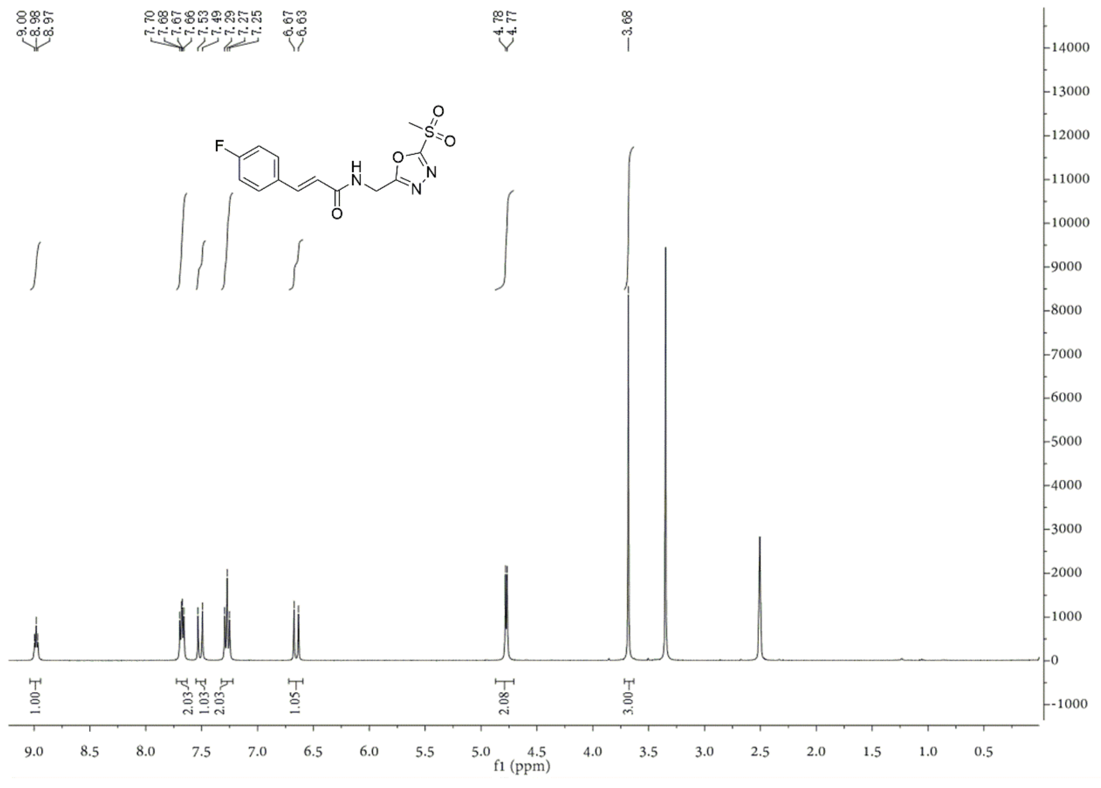

^{1}H NMR for compound **10**

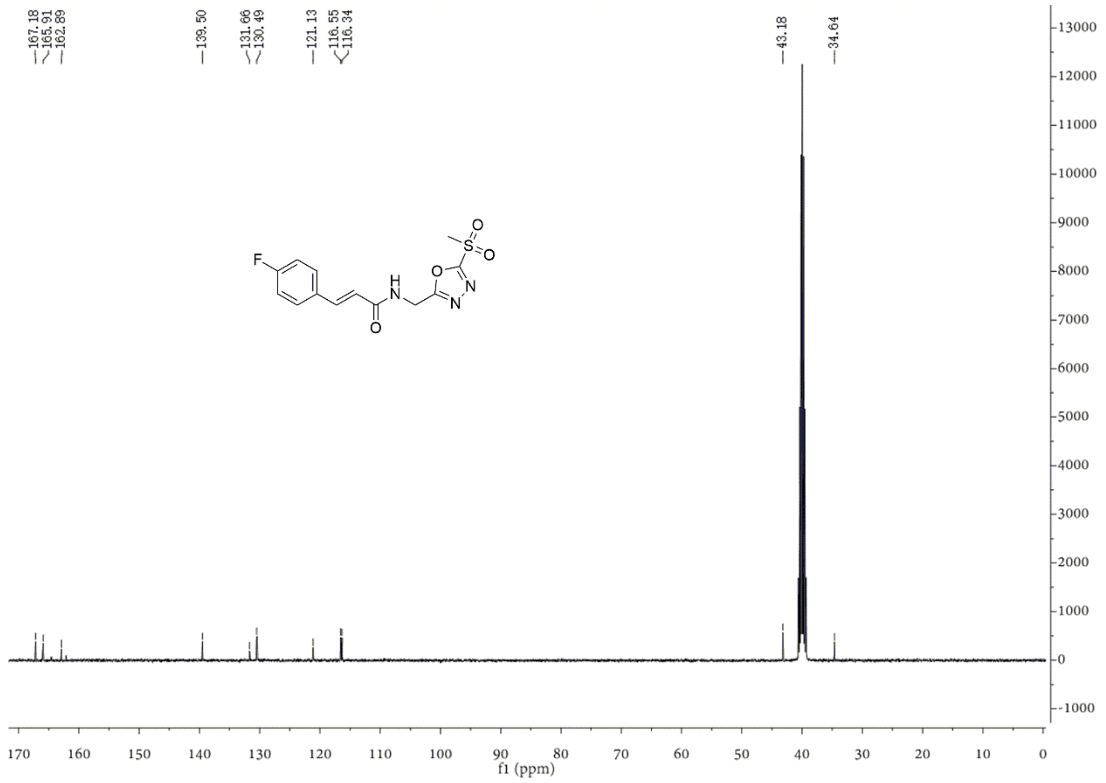

^{13}C NMR for compound **10**

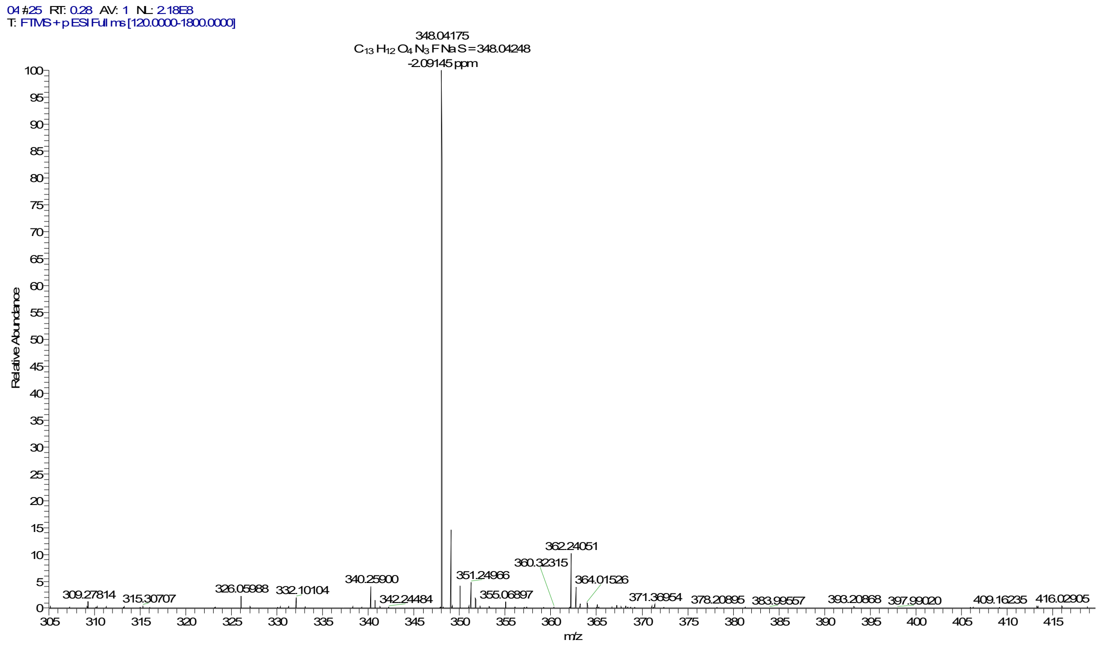

HRMS for compound **10**

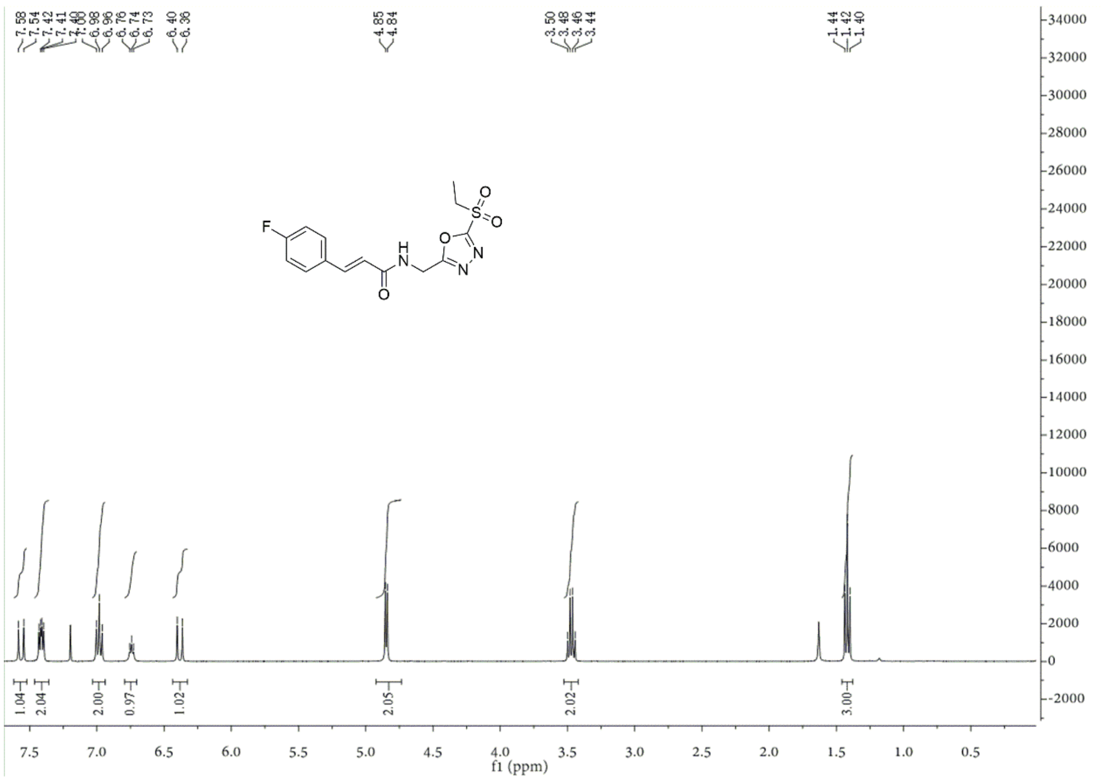

^{1}H NMR for compound **11**

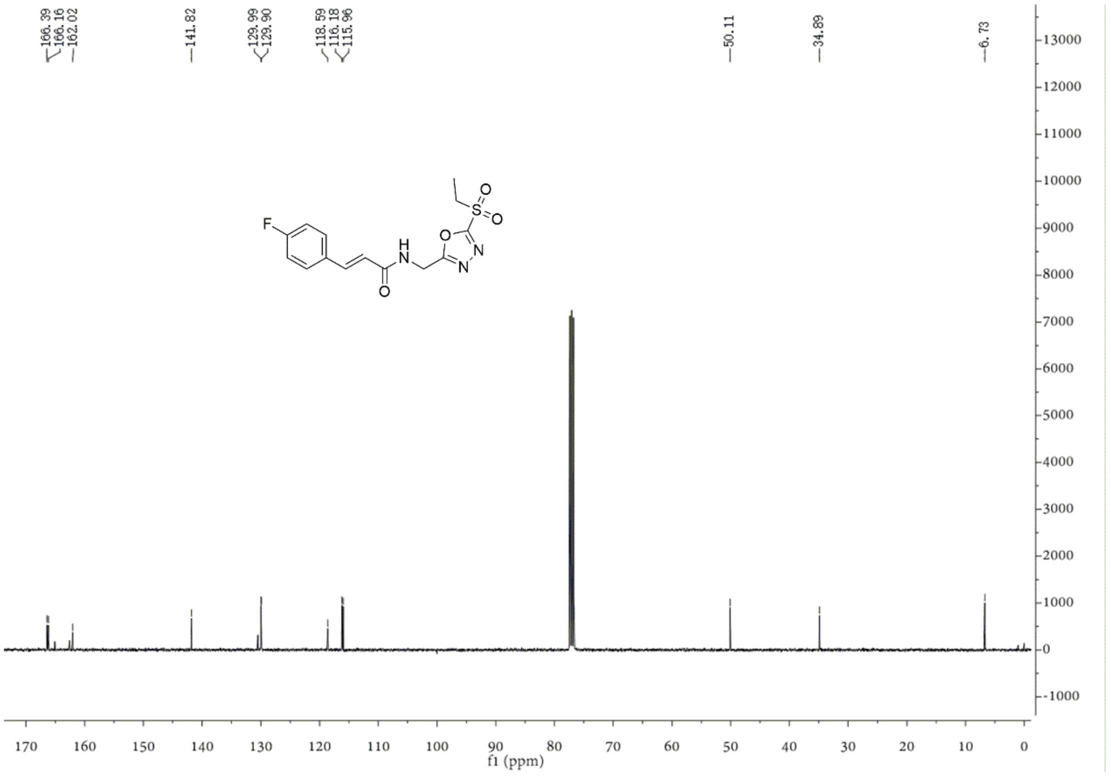

^{13}C NMR for compound **11**

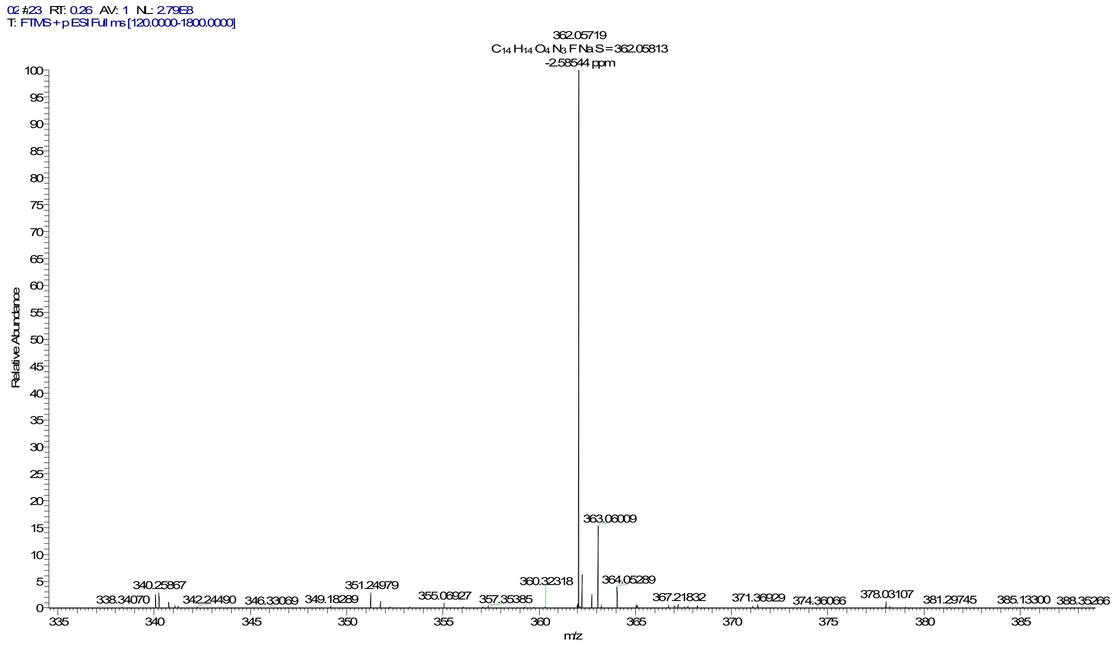

HRMS for compound **11**

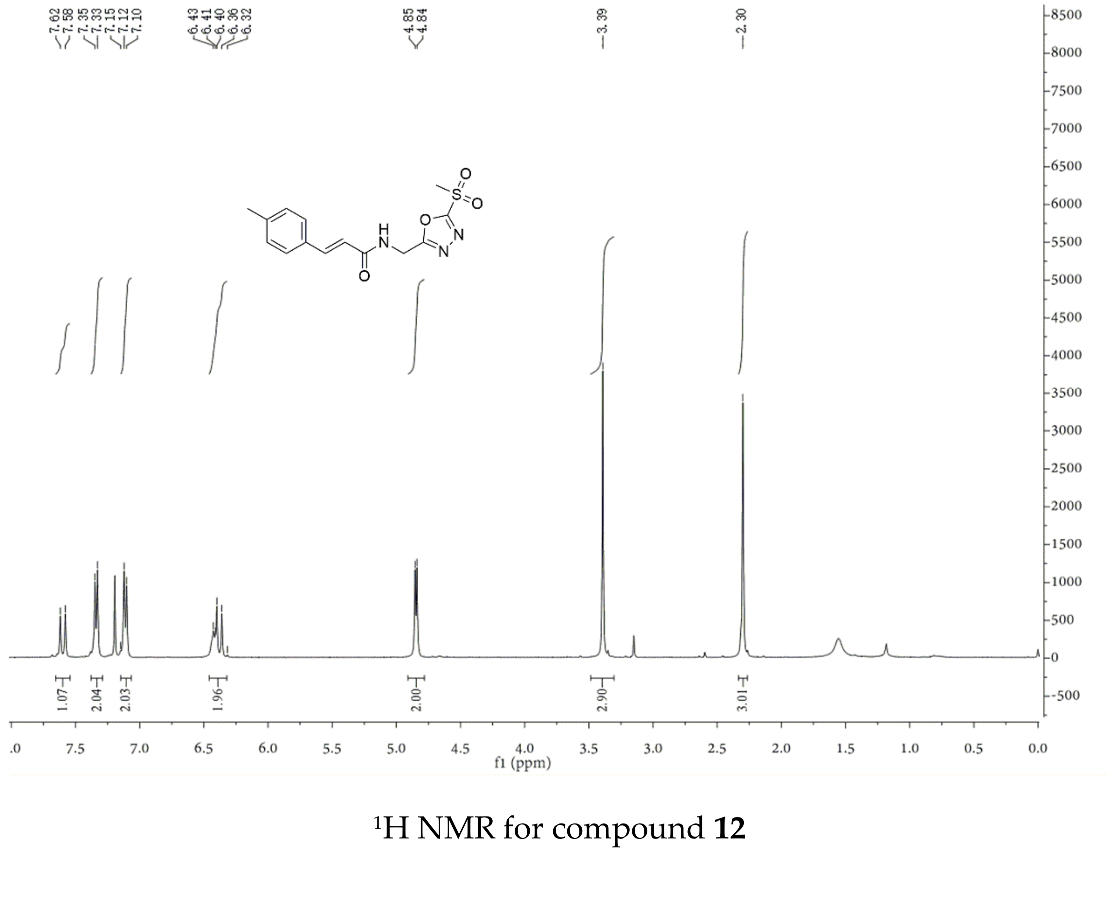

^{1}H NMR for compound **12**

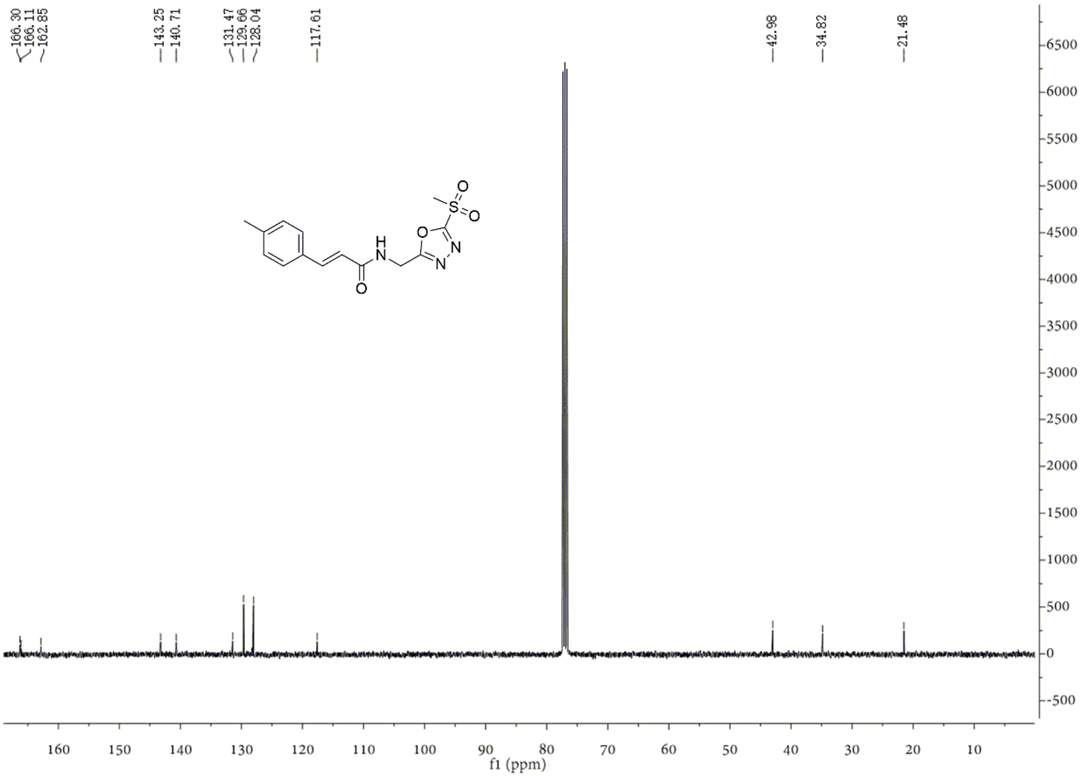

^{13}C NMR for compound **12**

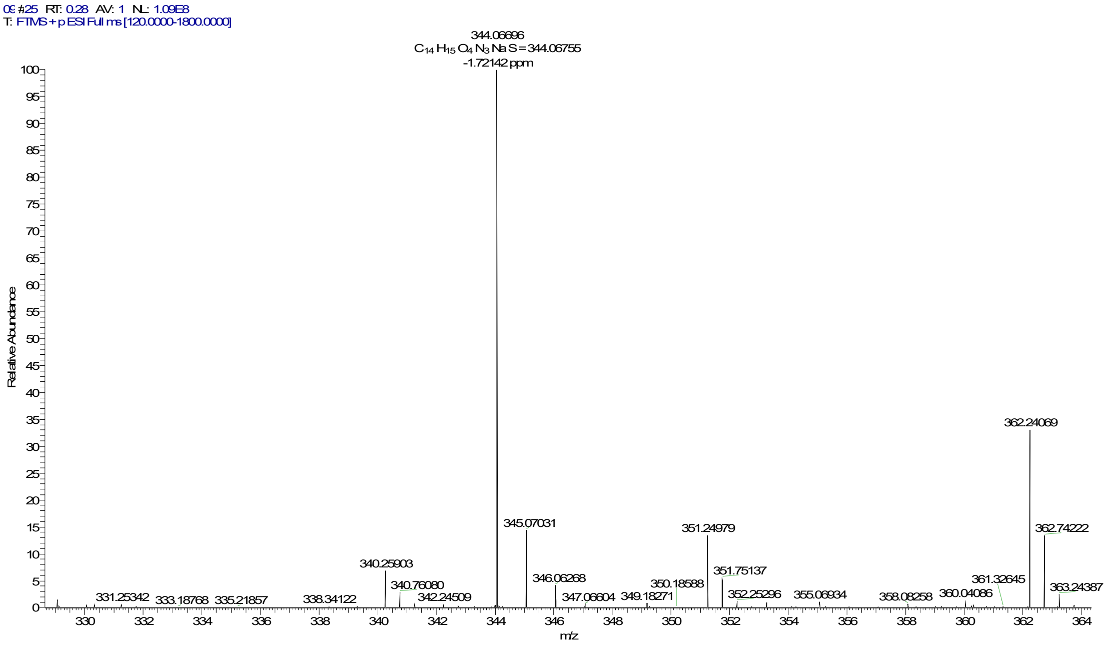

HRMS for compound **12**

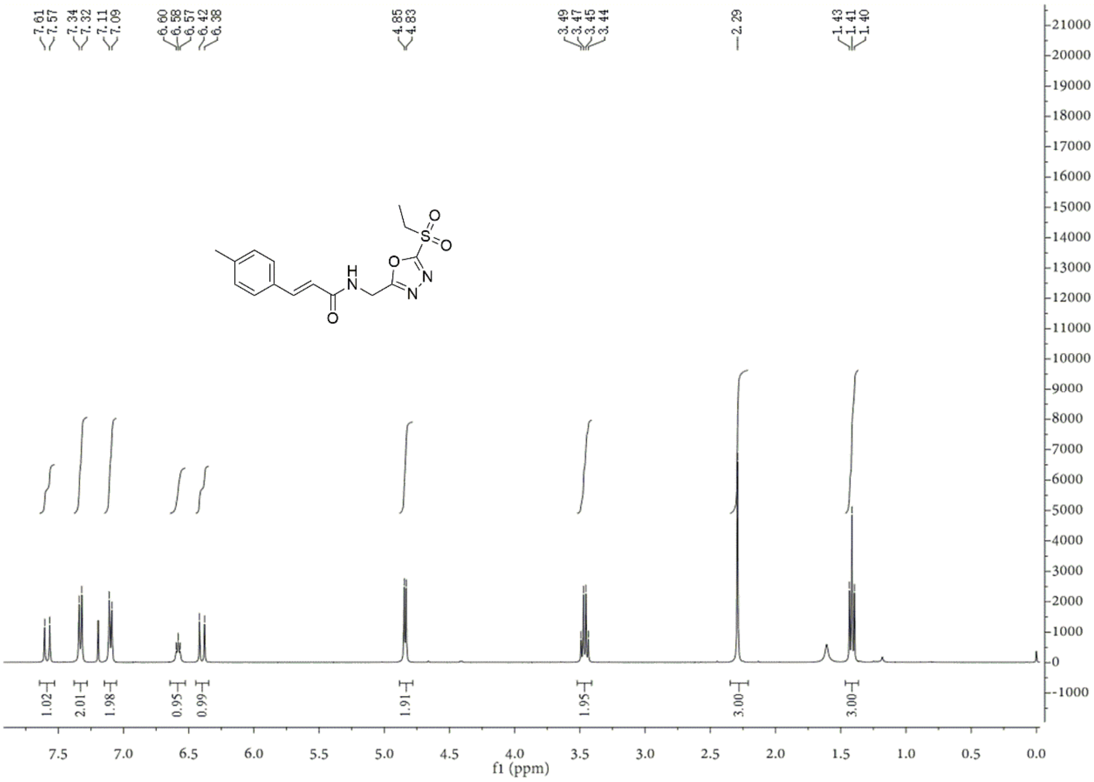

^{1}H NMR for compound **13**

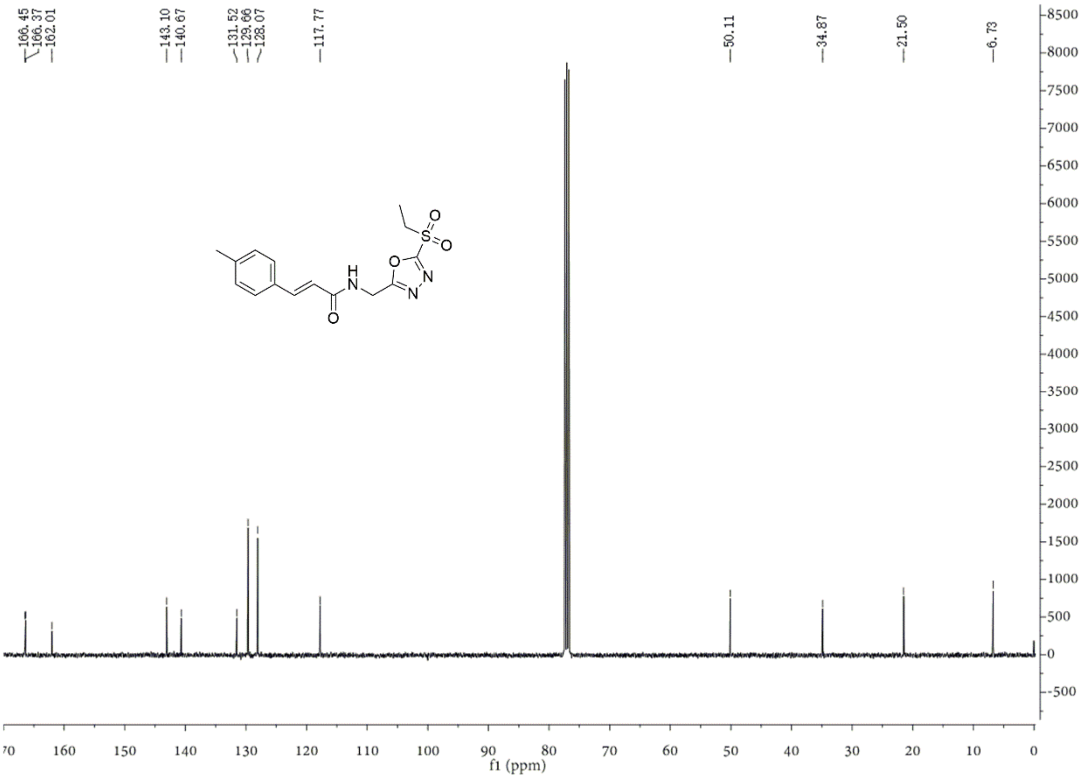

^{13}C NMR for compound **13**

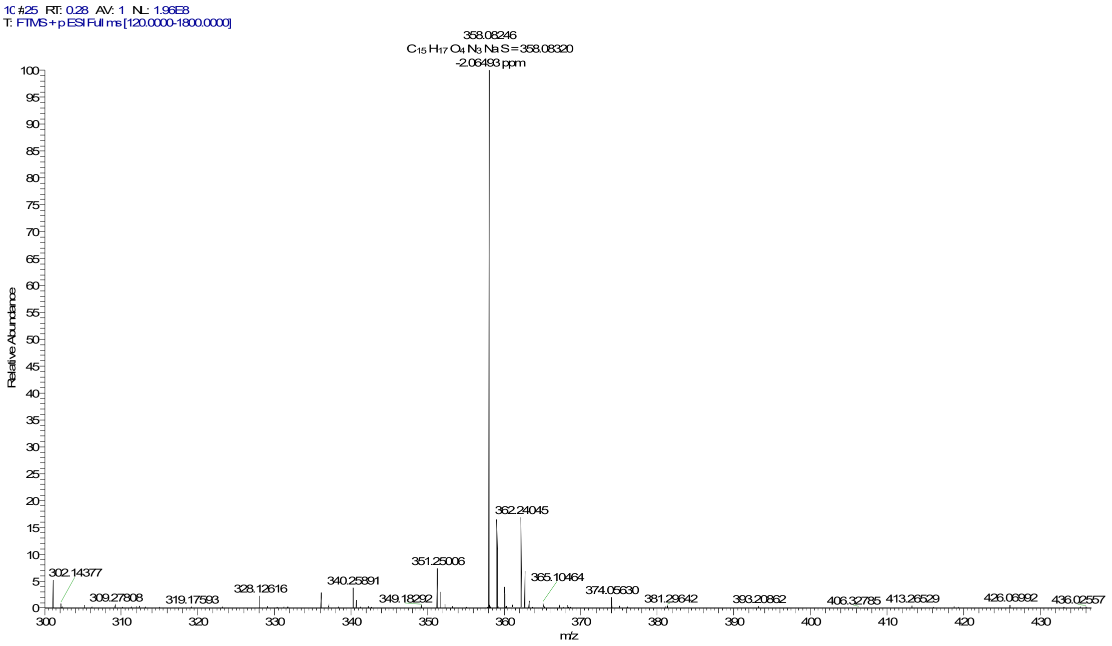

HRMS for compound **13**

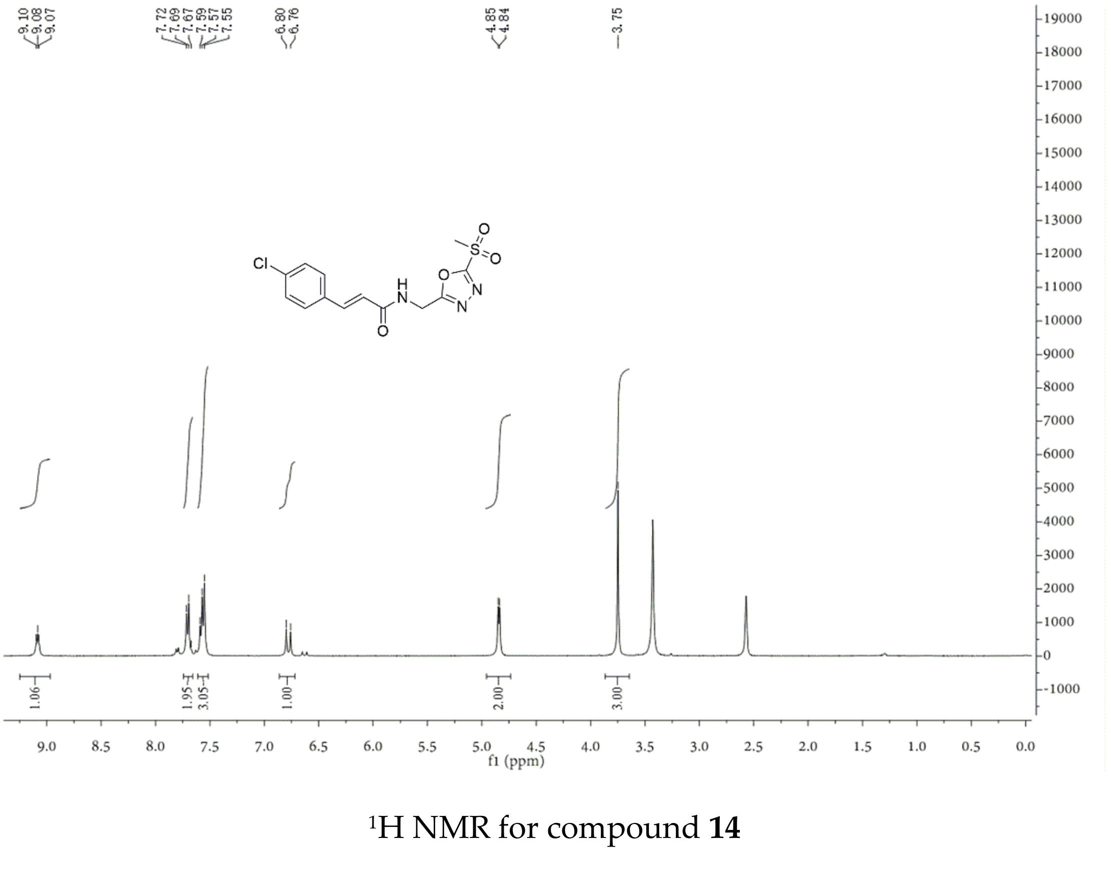

^{1}H NMR for compound **14**

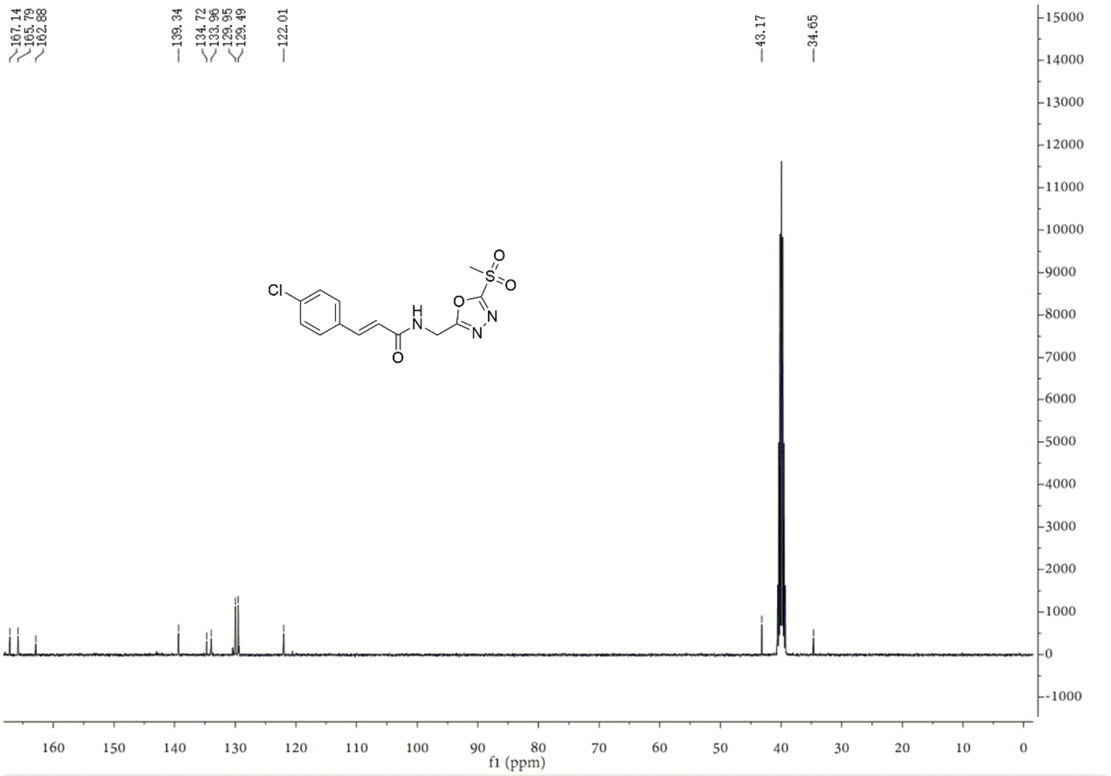

^{13}C NMR for compound **14**

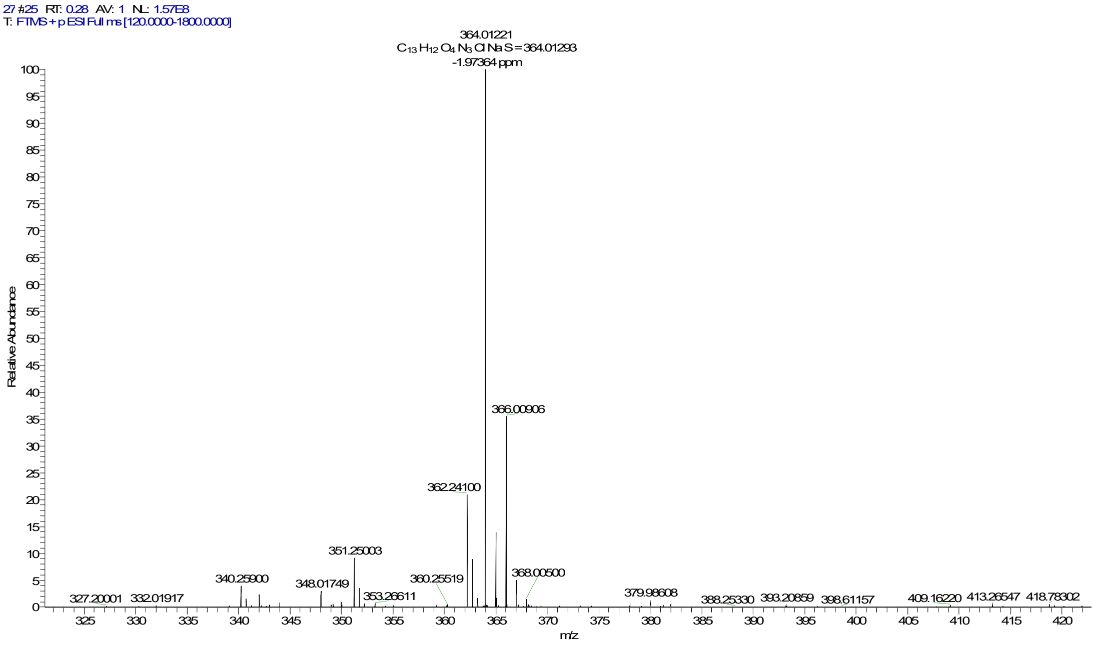

HRMS for compound **14**

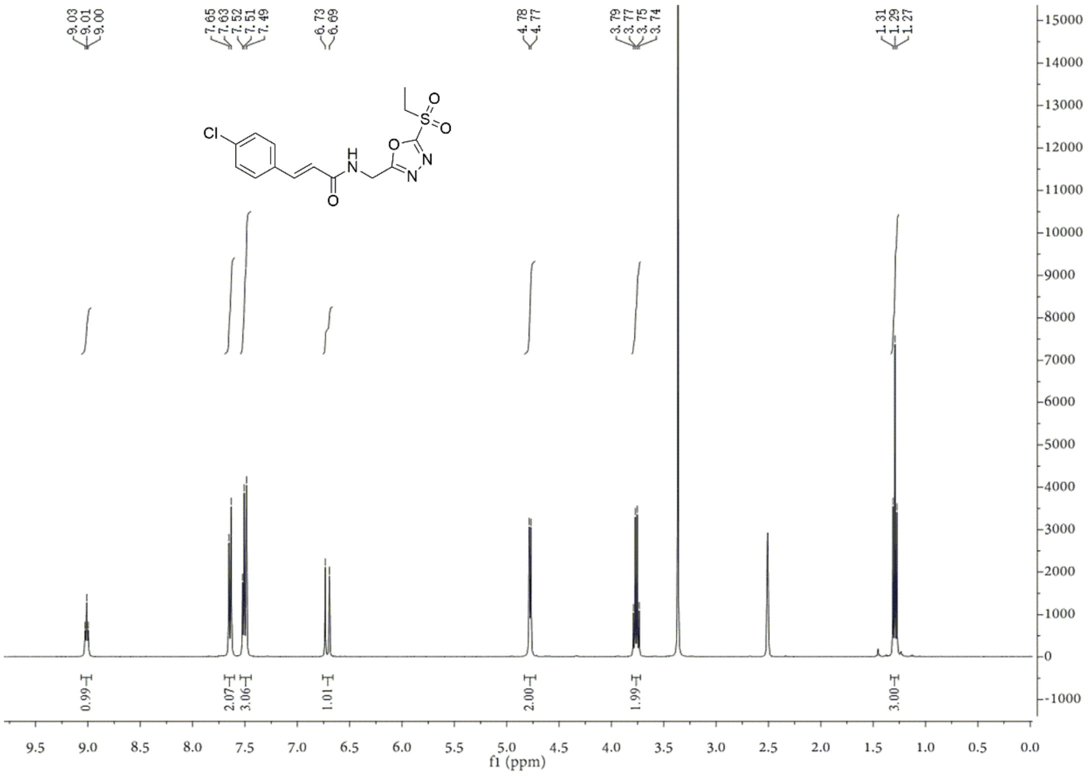

^{1}H NMR for compound **15**

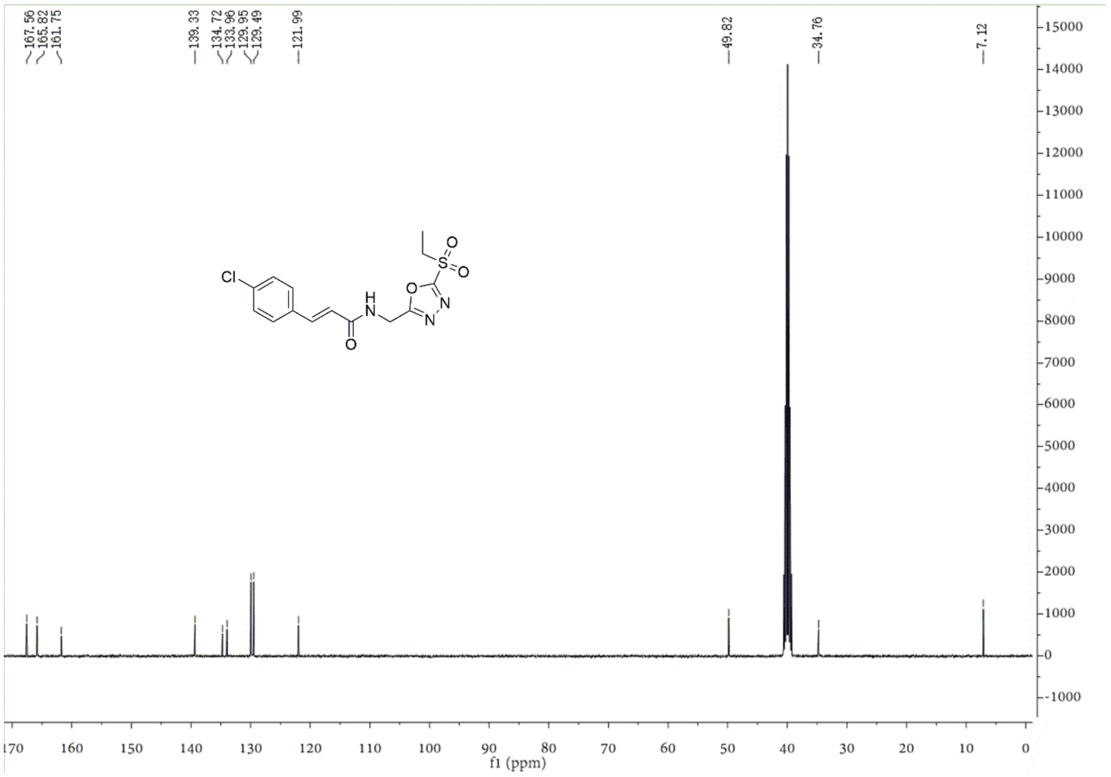

^{13}C NMR for compound **15**

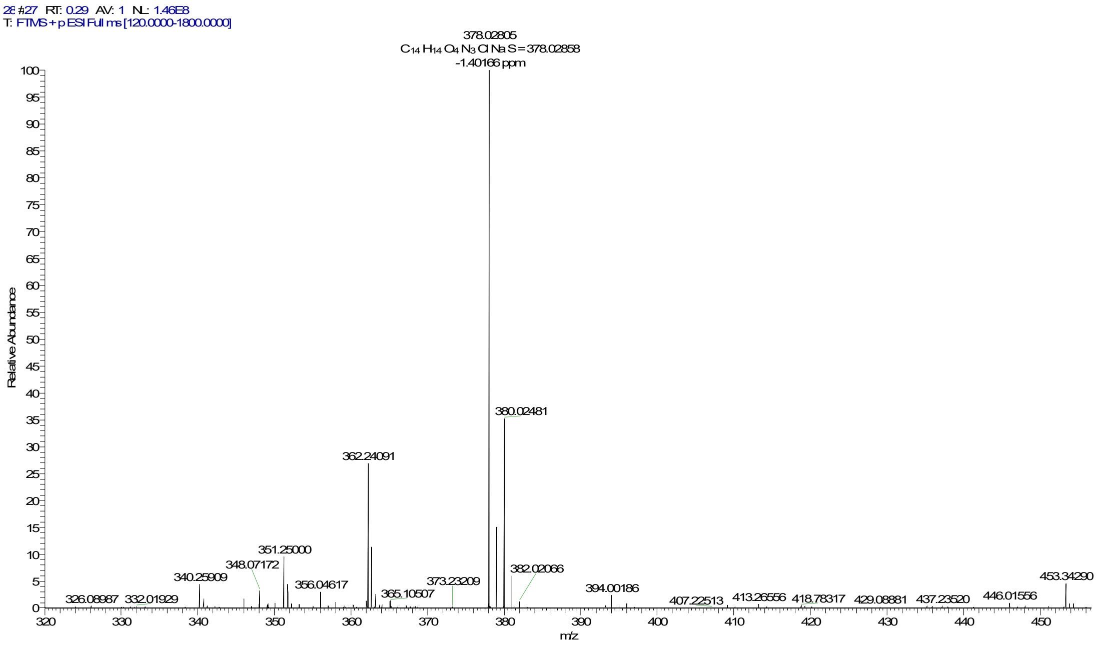

HRMS for compound **15**

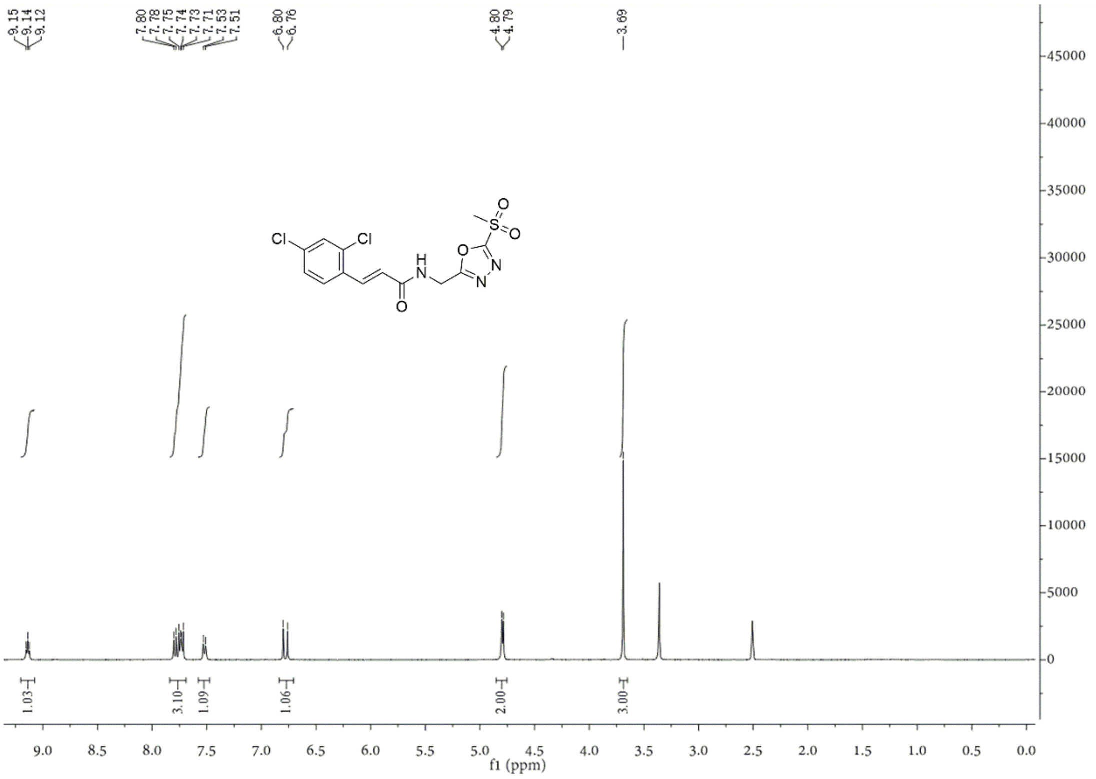

^{1}H NMR for compound **16**

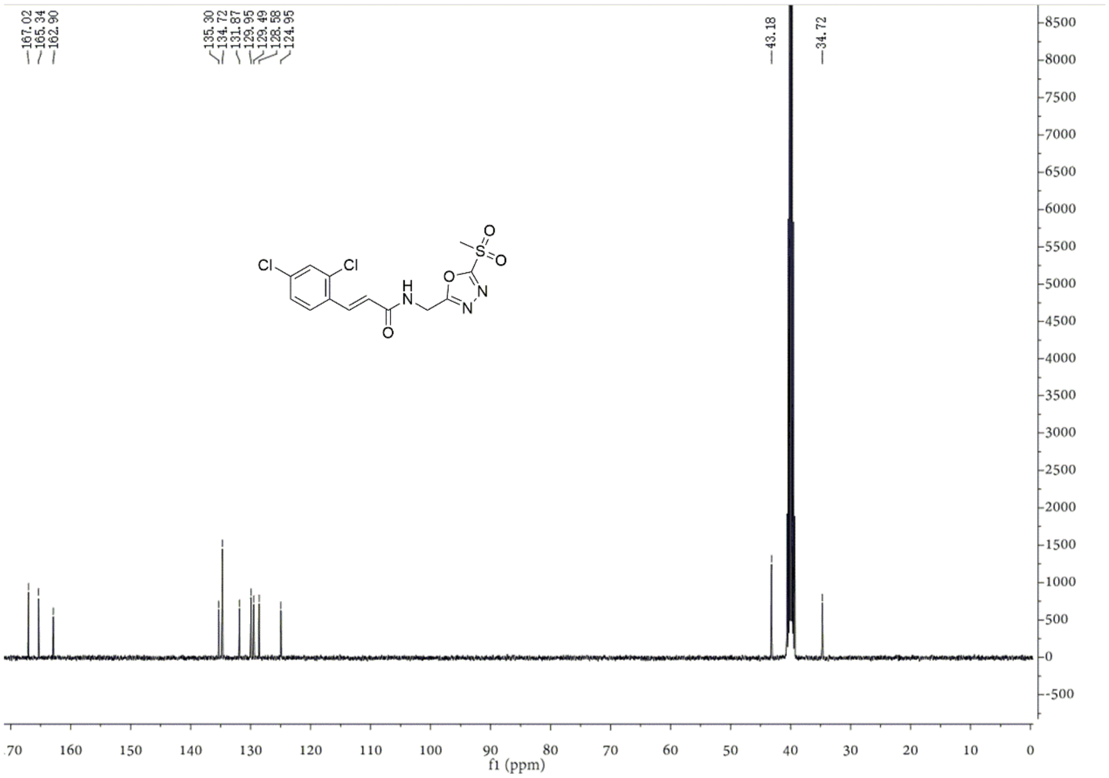

^{13}C NMR for compound **16**

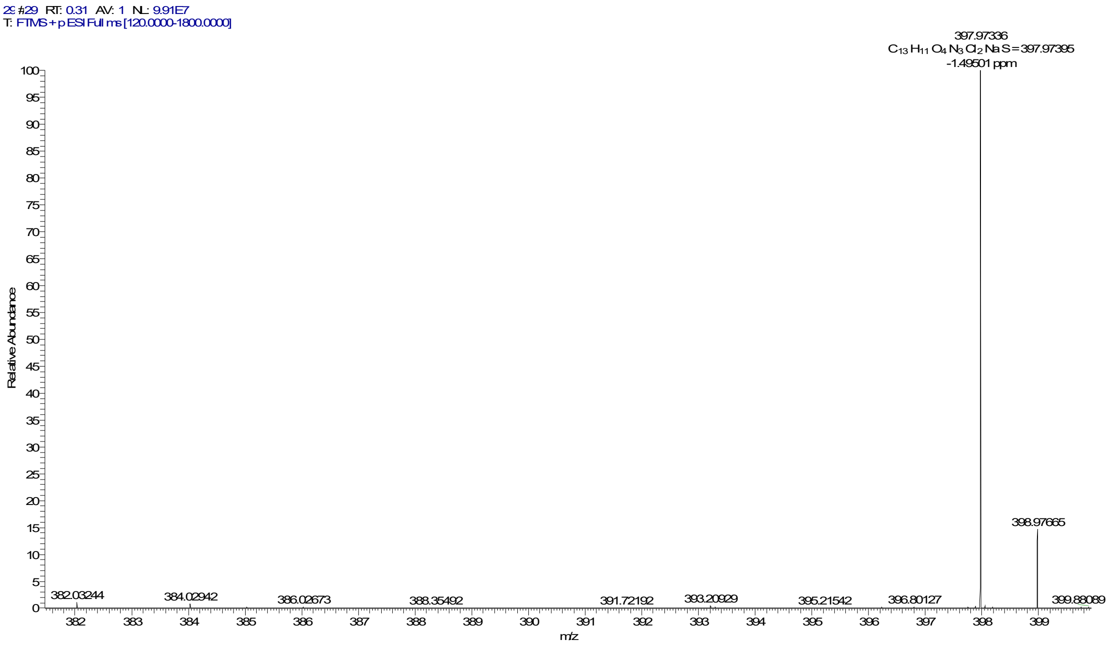

HRMS for compound **16**

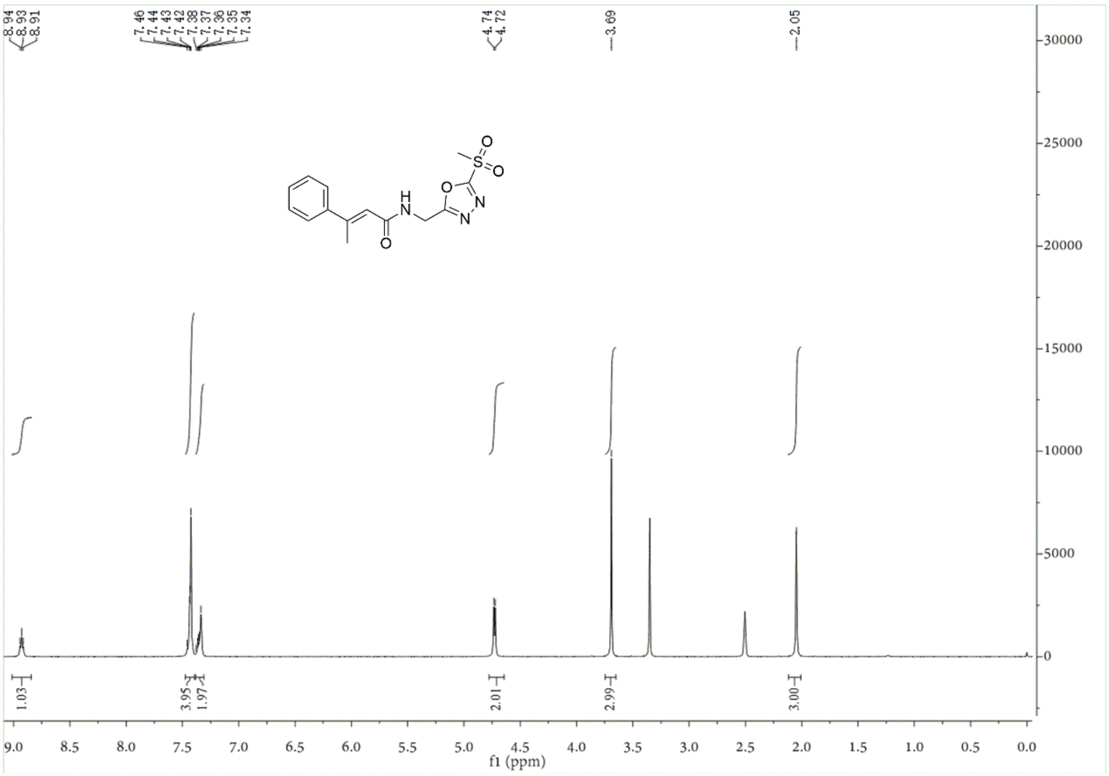

^{1}H NMR for compound **17**

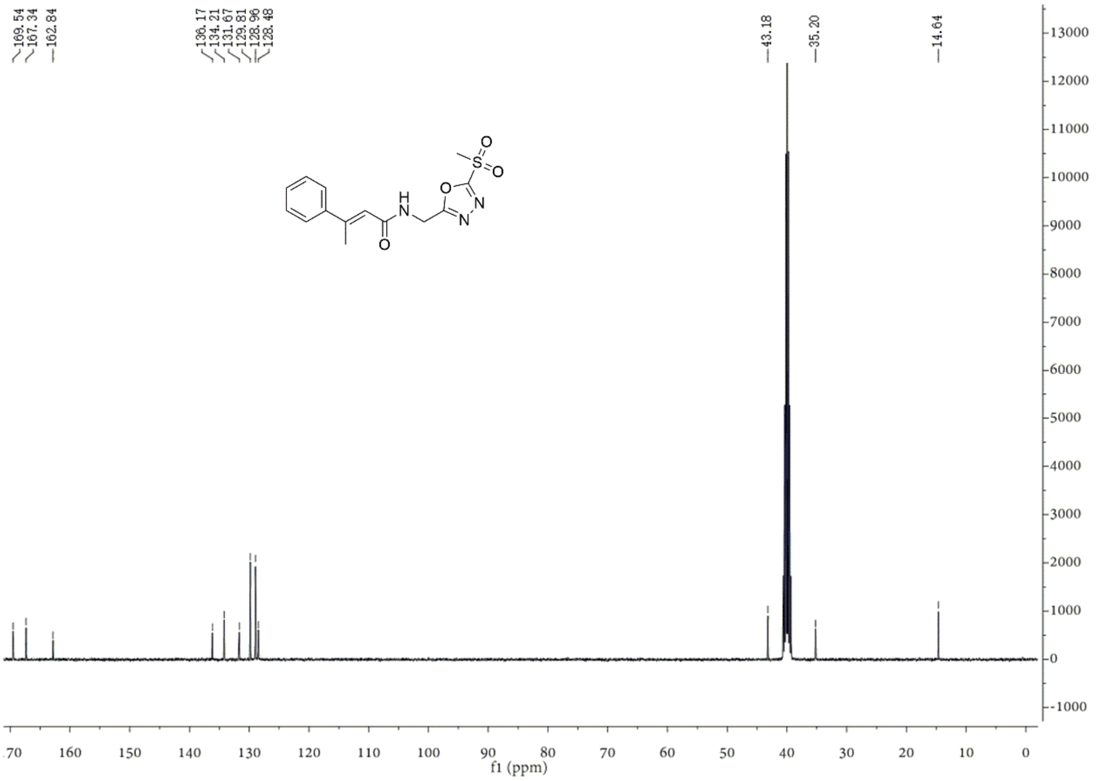

^{13}C NMR for compound **17**

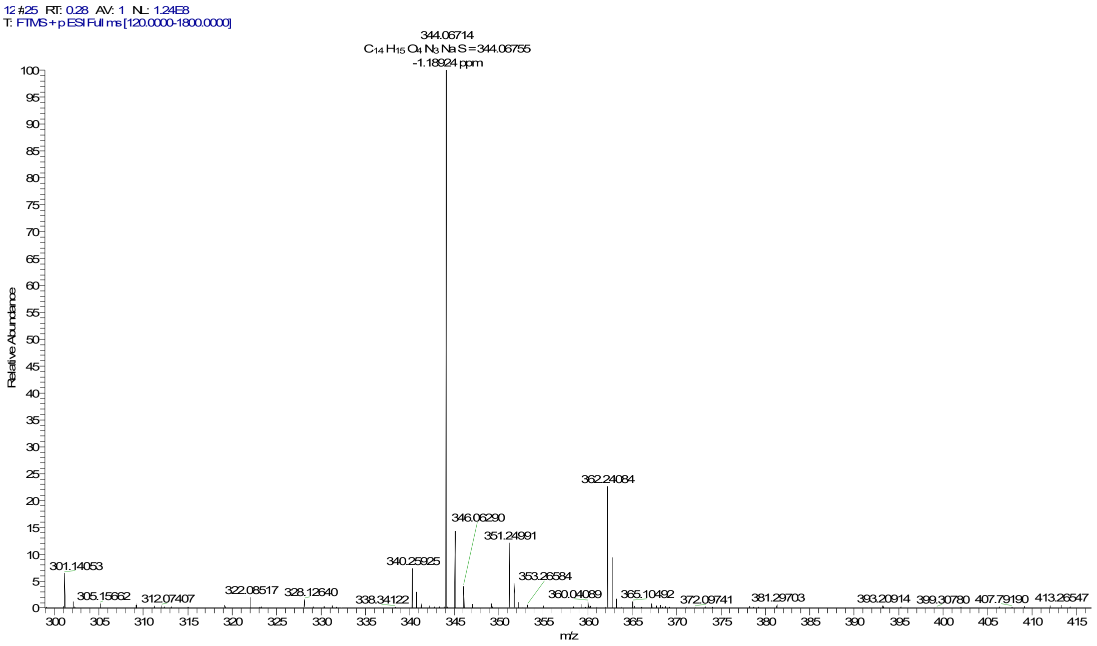

HRMS for compound **17**

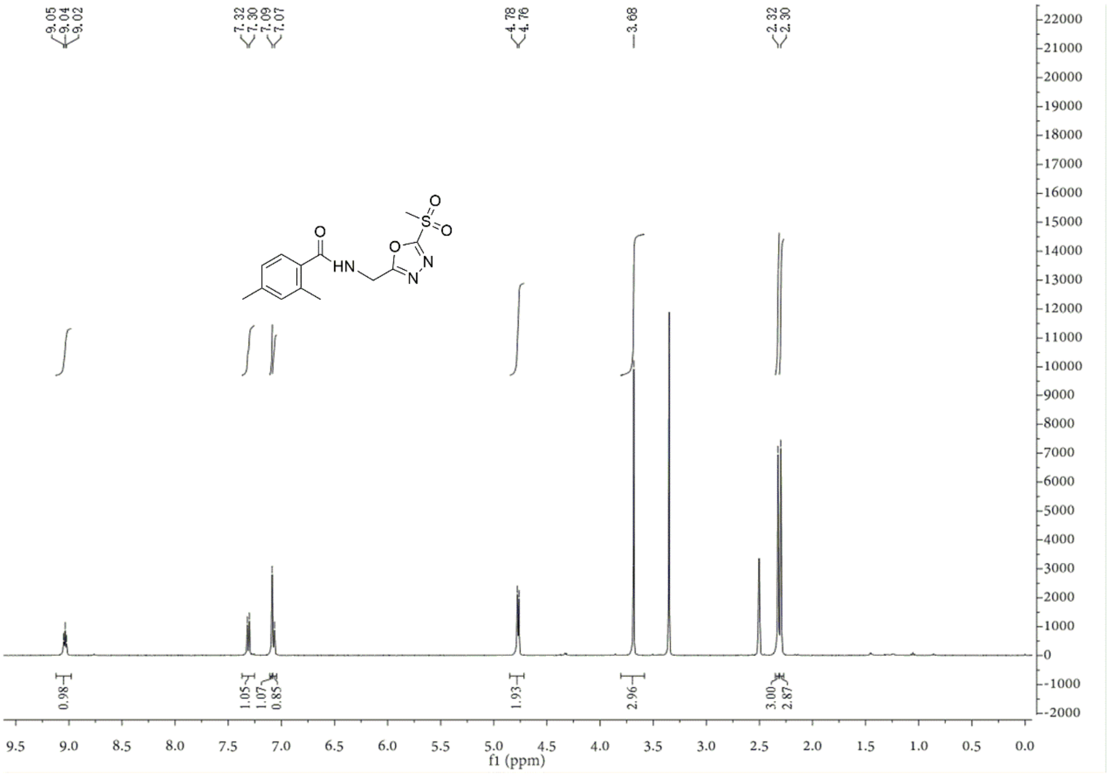

^{1}H NMR for compound **18**

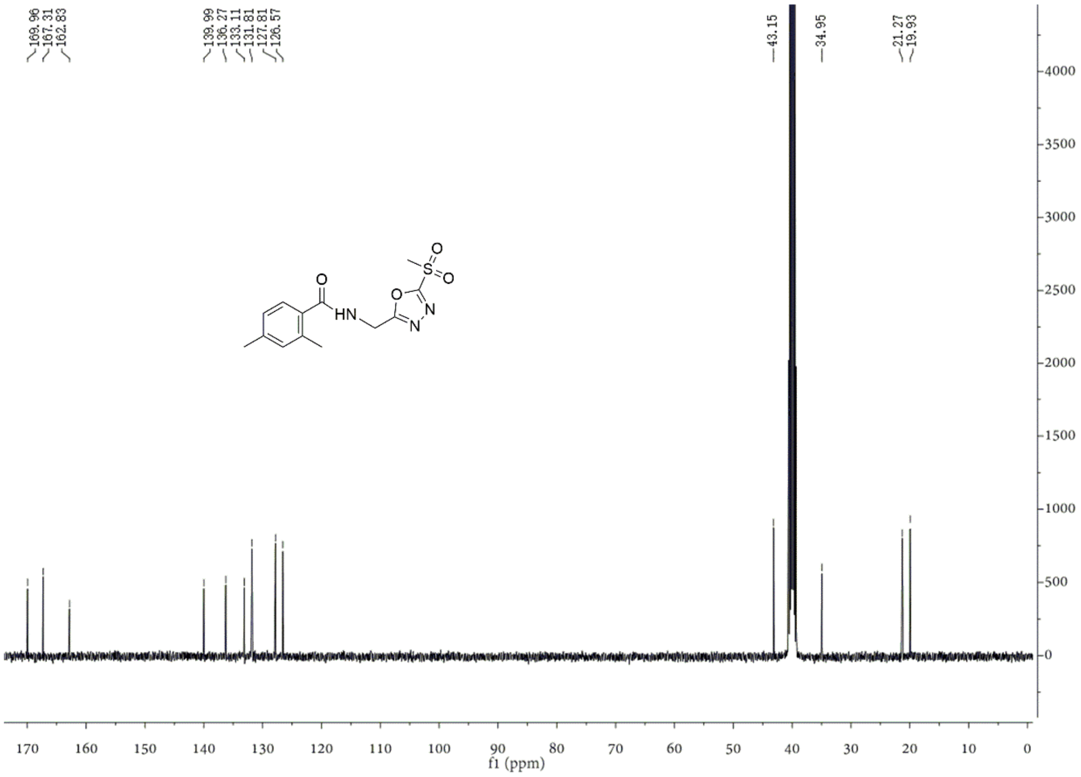

^{13}C NMR for compound **18**

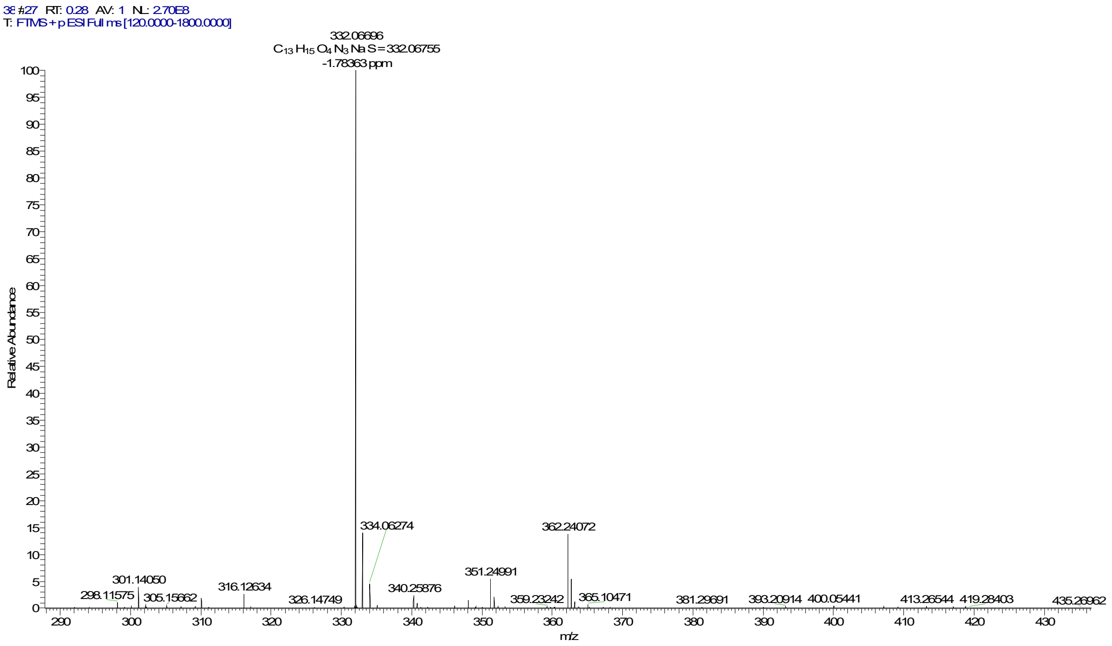

HRMS for compound **18**

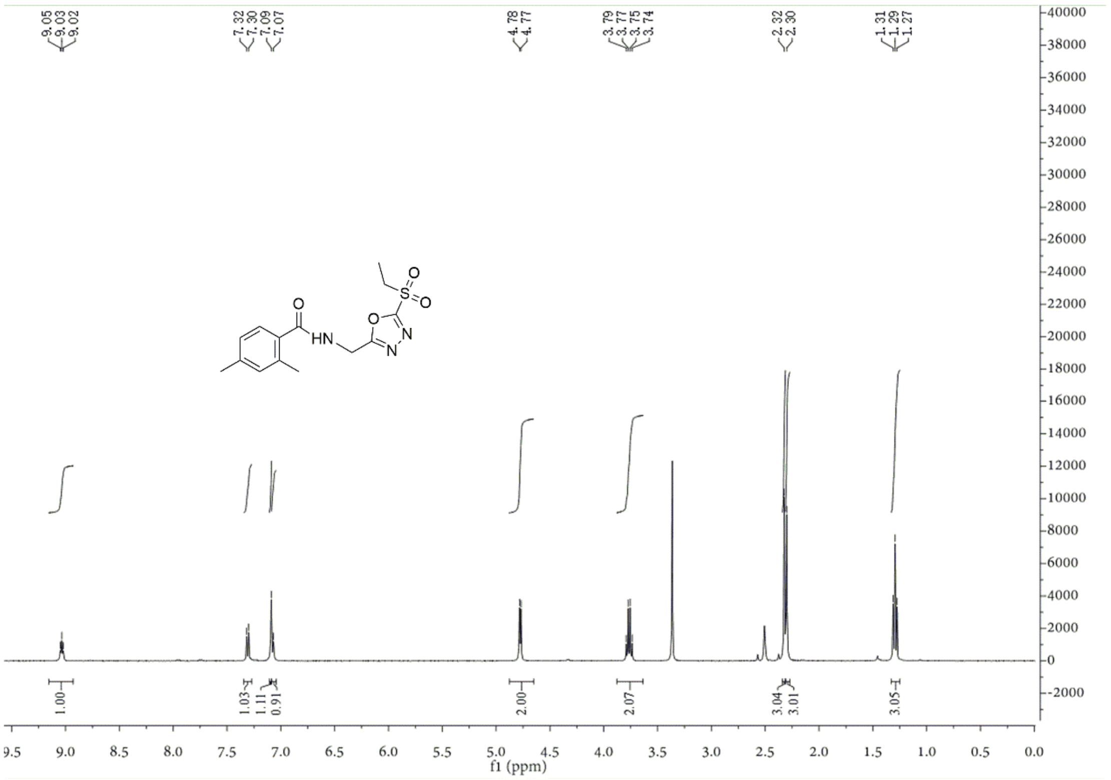

^{1}H NMR for compound **19**

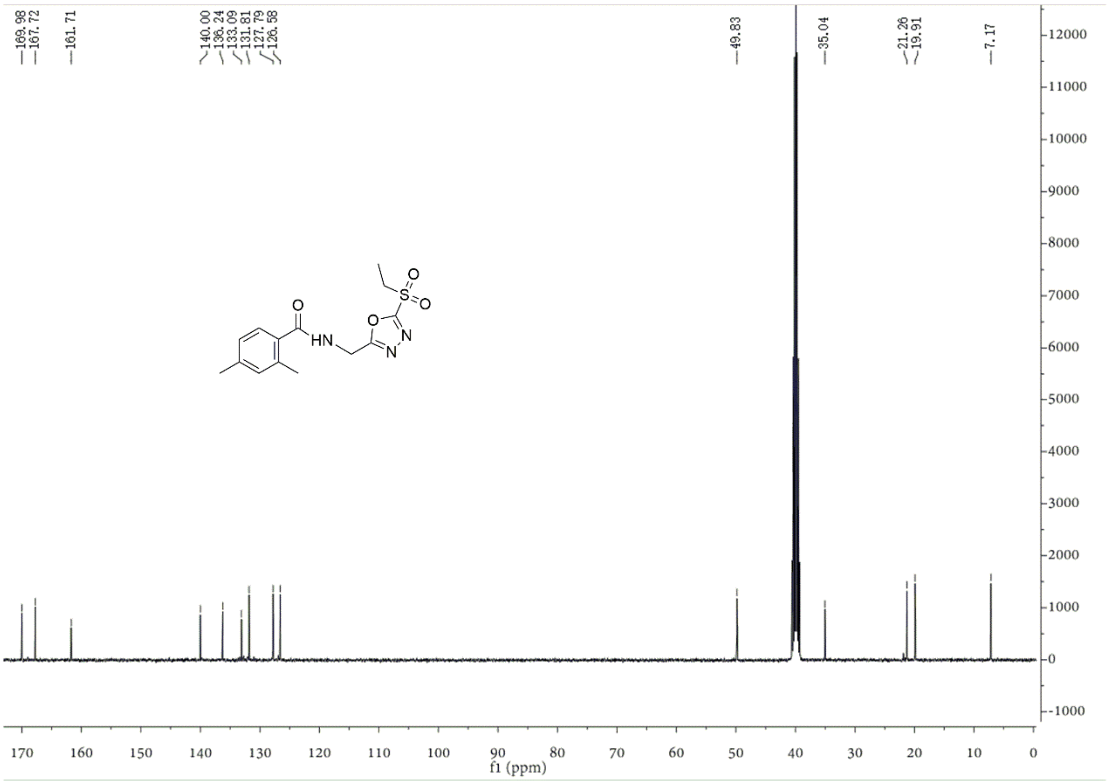

^{13}C NMR for compound **19**

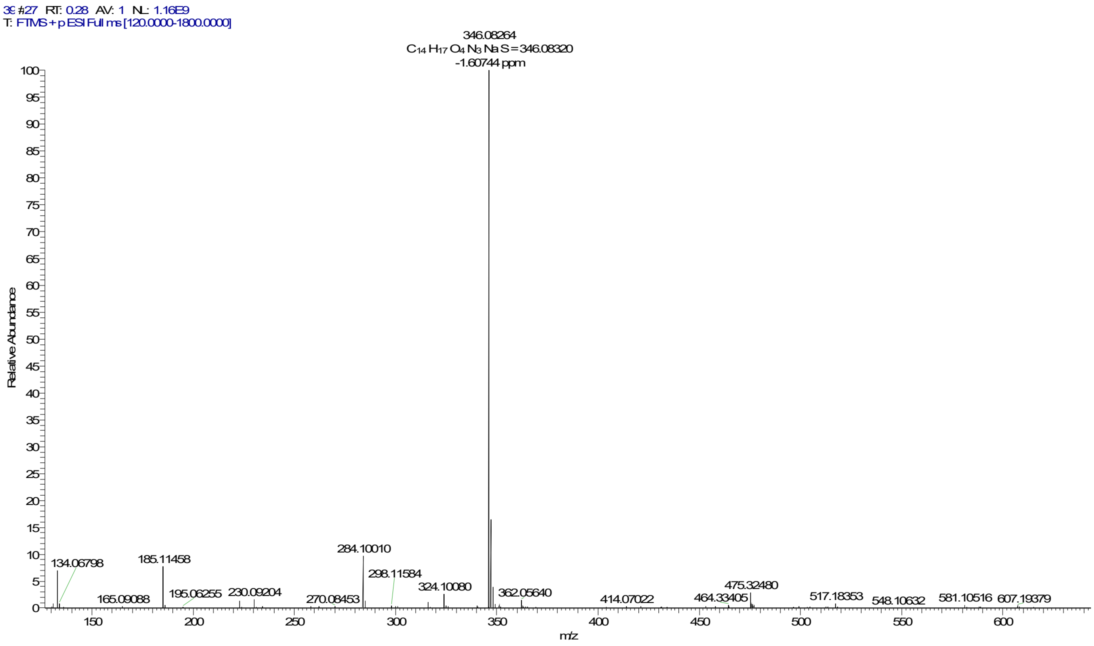

HRMS for compound **19**

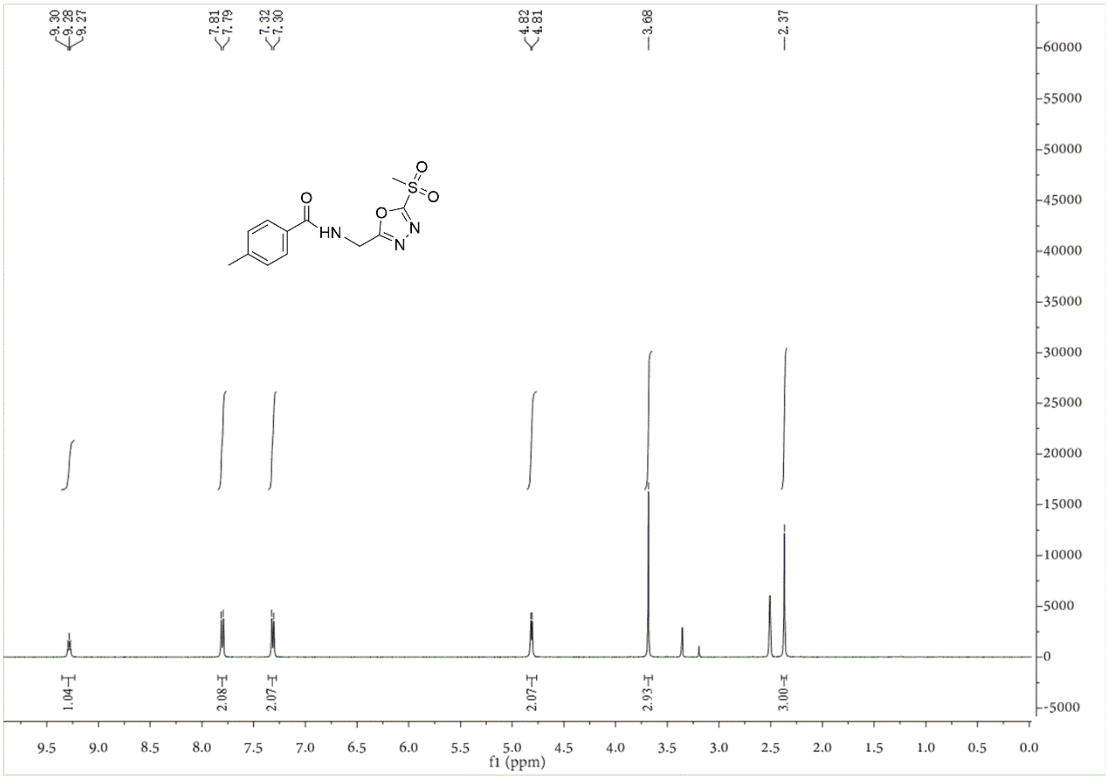

^{1}H NMR for compound **20**

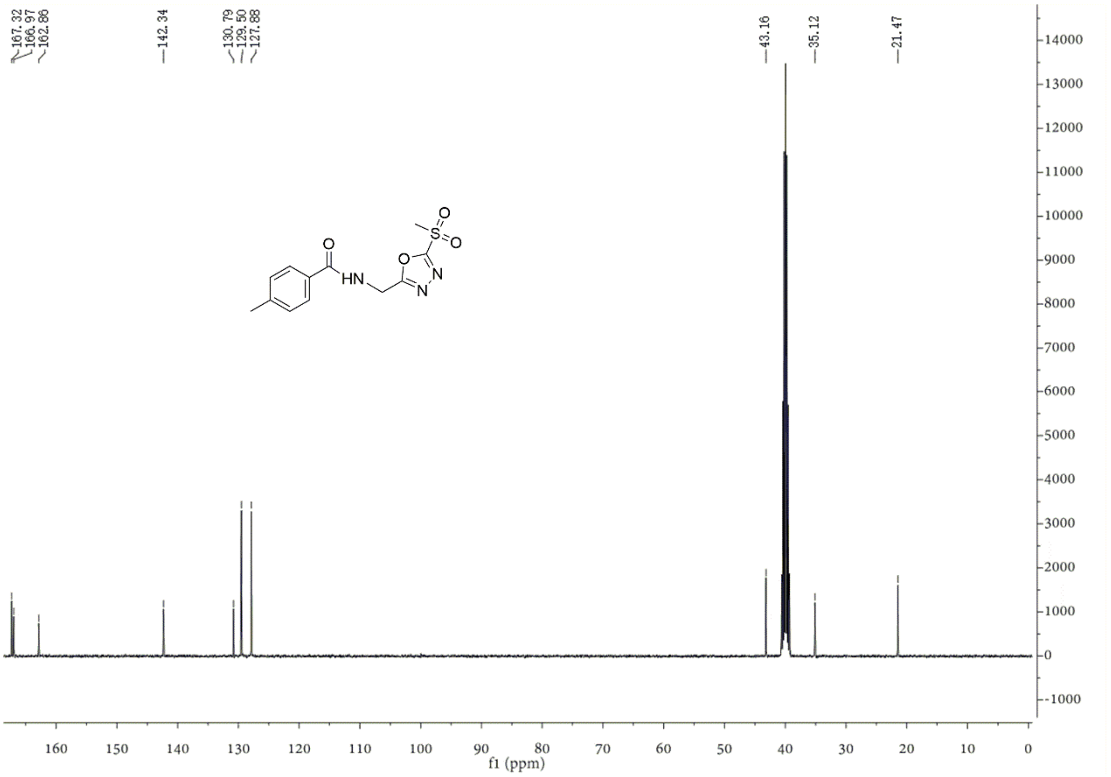

^{13}C NMR for compound **20**

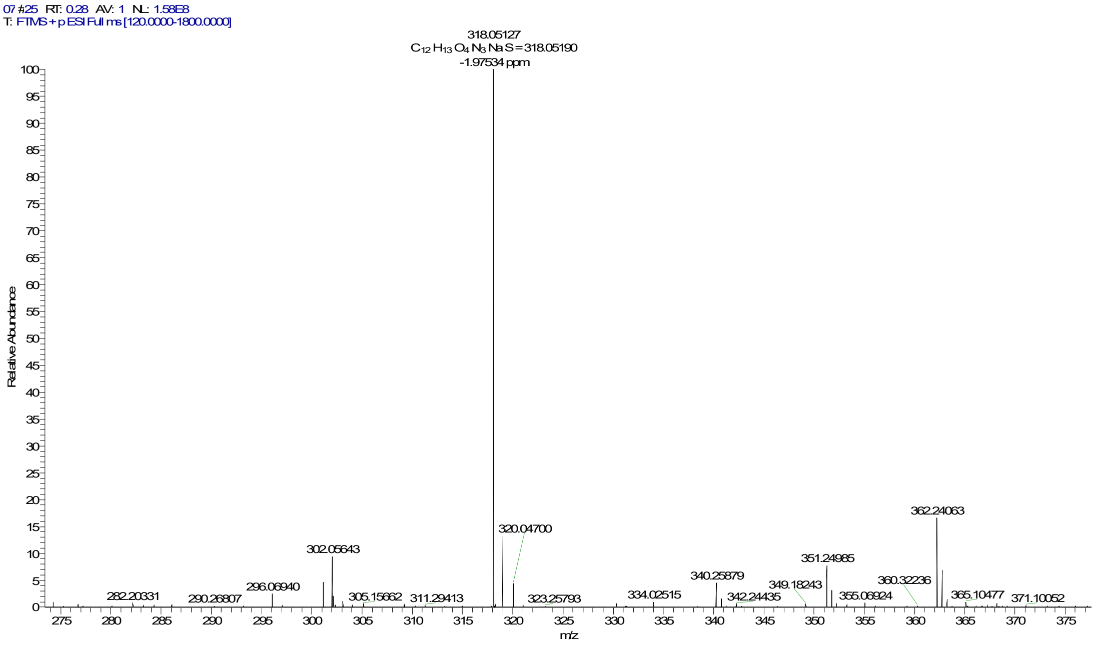

HRMS for compound **20**

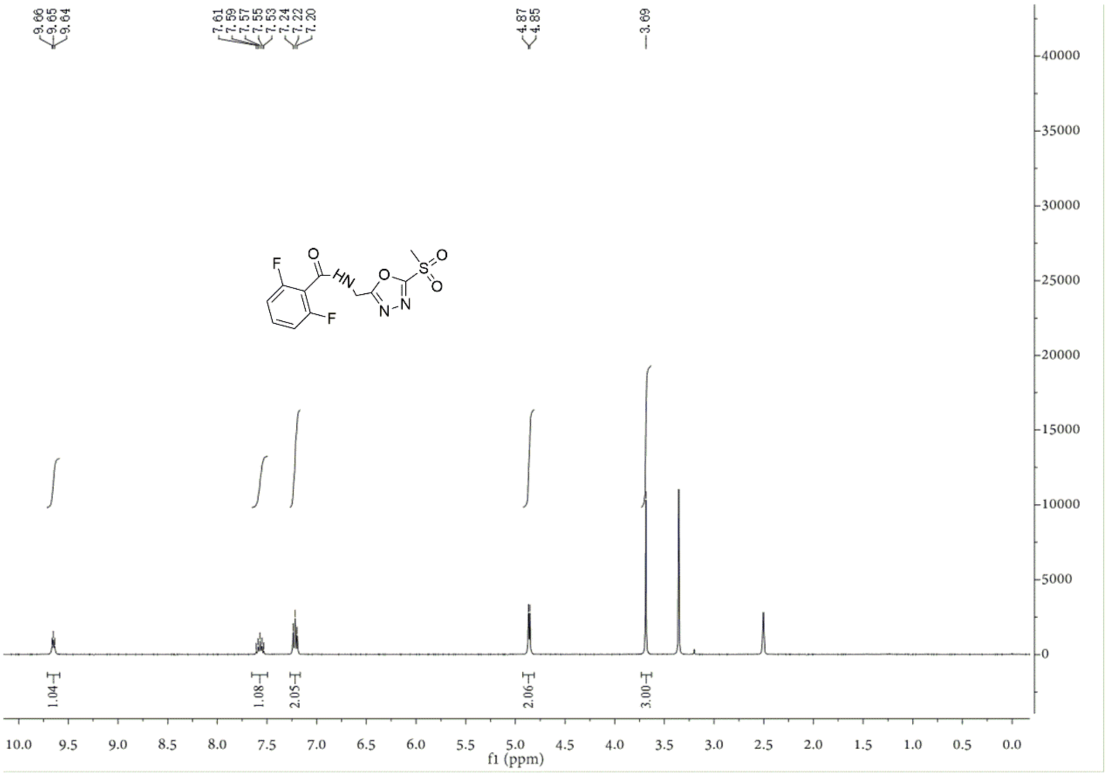

^{1}H NMR for compound **21**

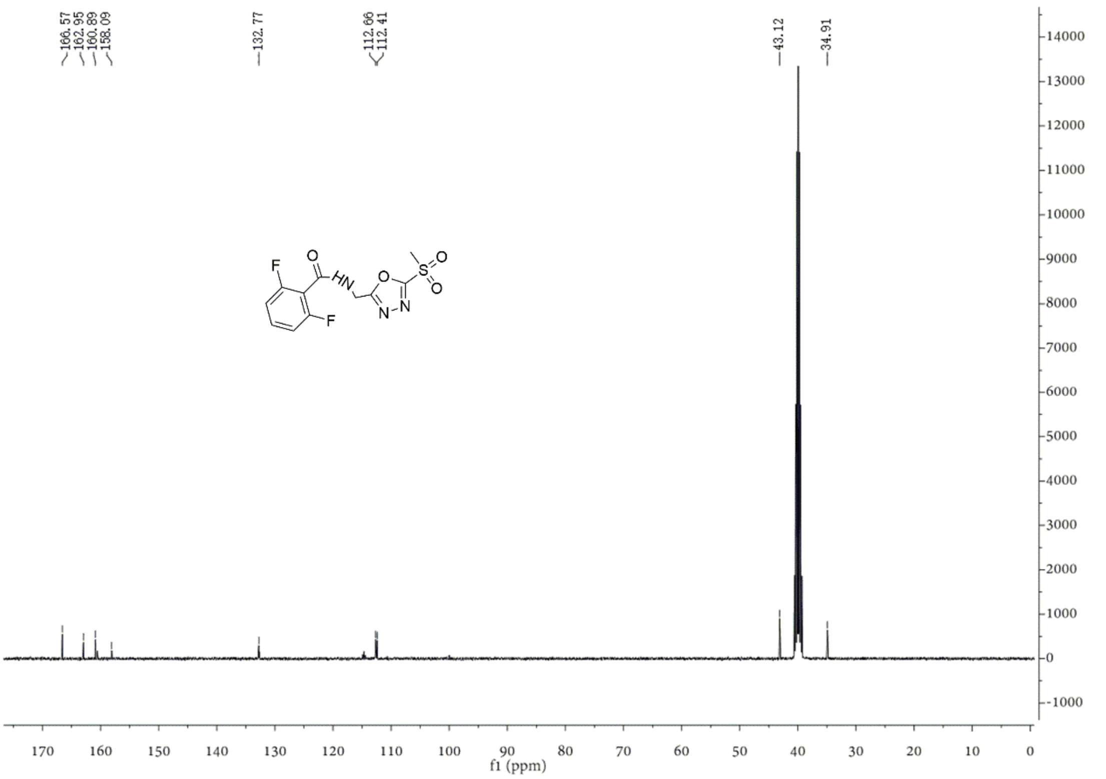

^{13}C NMR for compound **21**

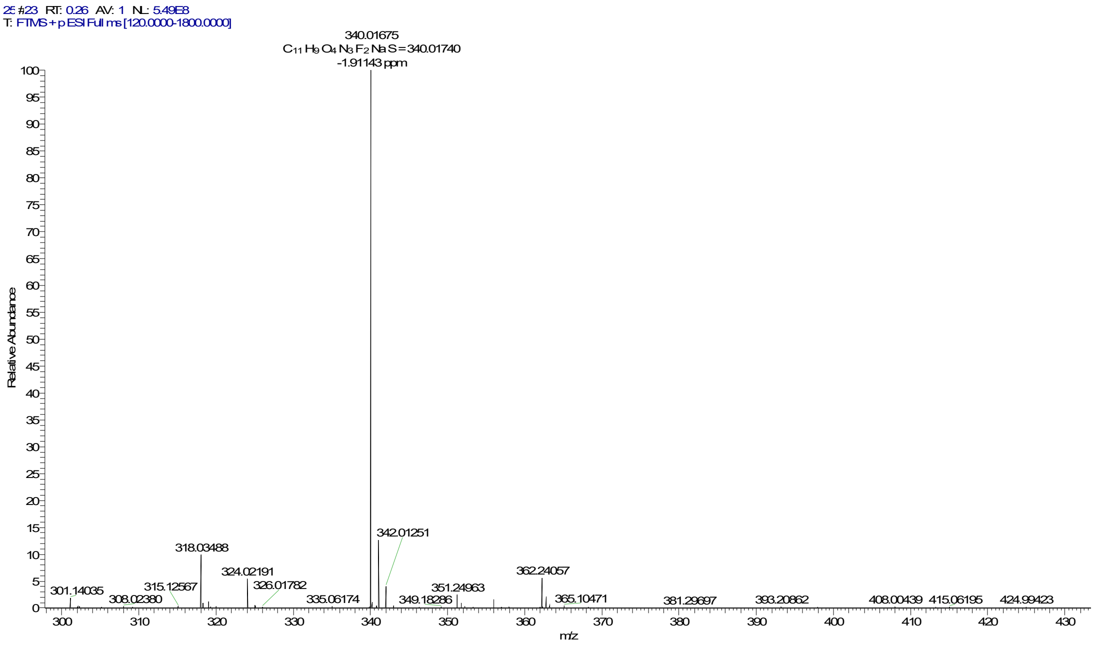

HRMS for compound **21**

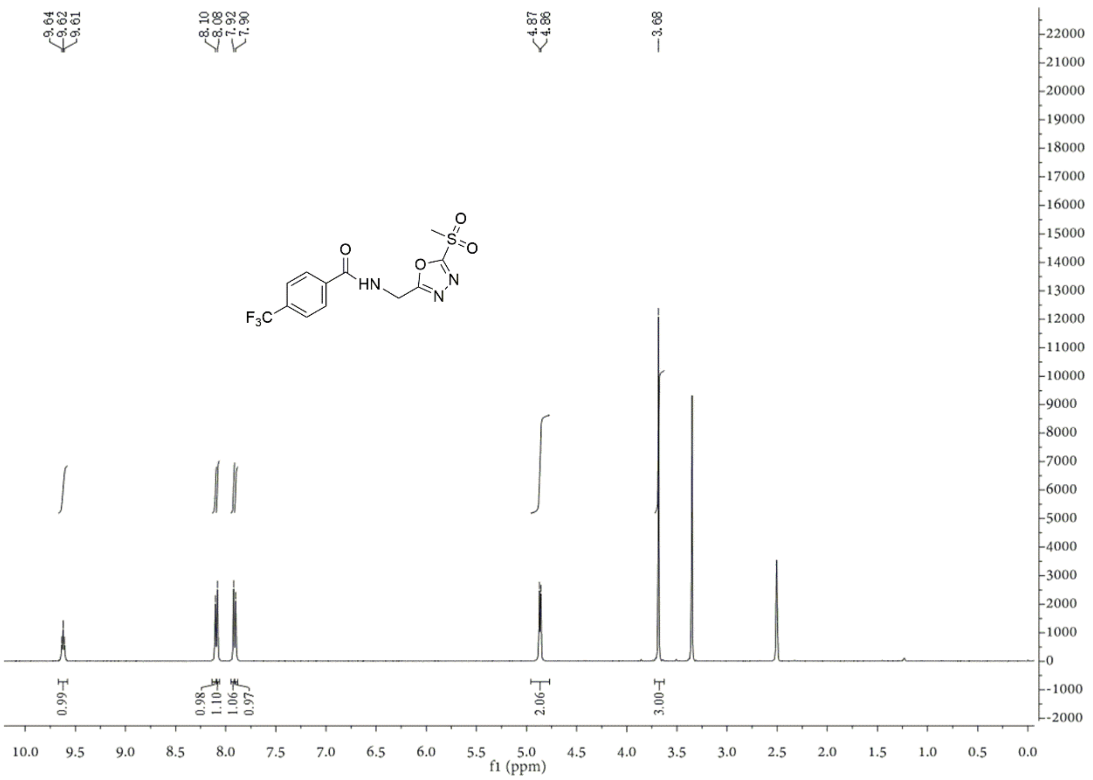

^{1}H NMR for compound **22**

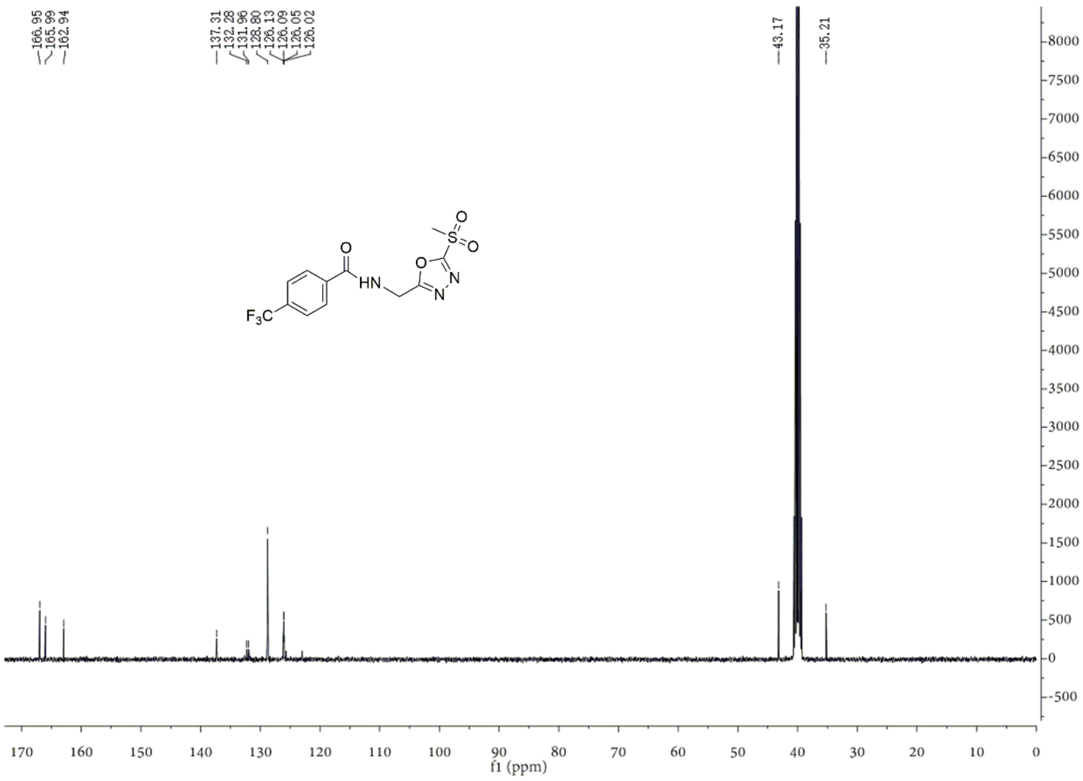

^{13}C NMR for compound **22**

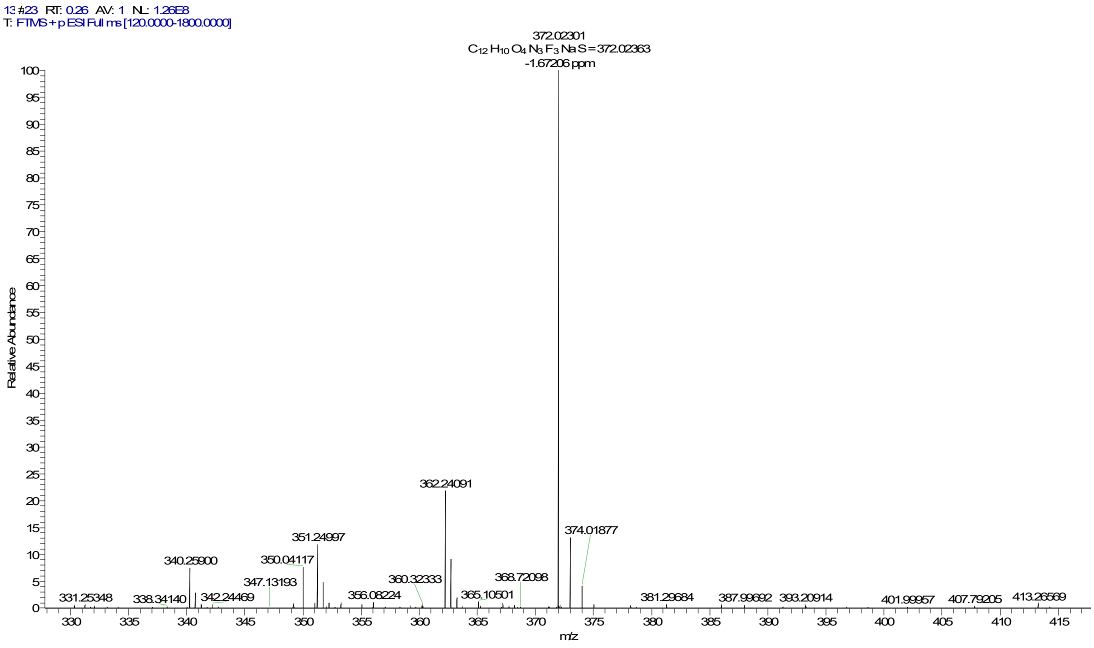

HRMS for compound **22**

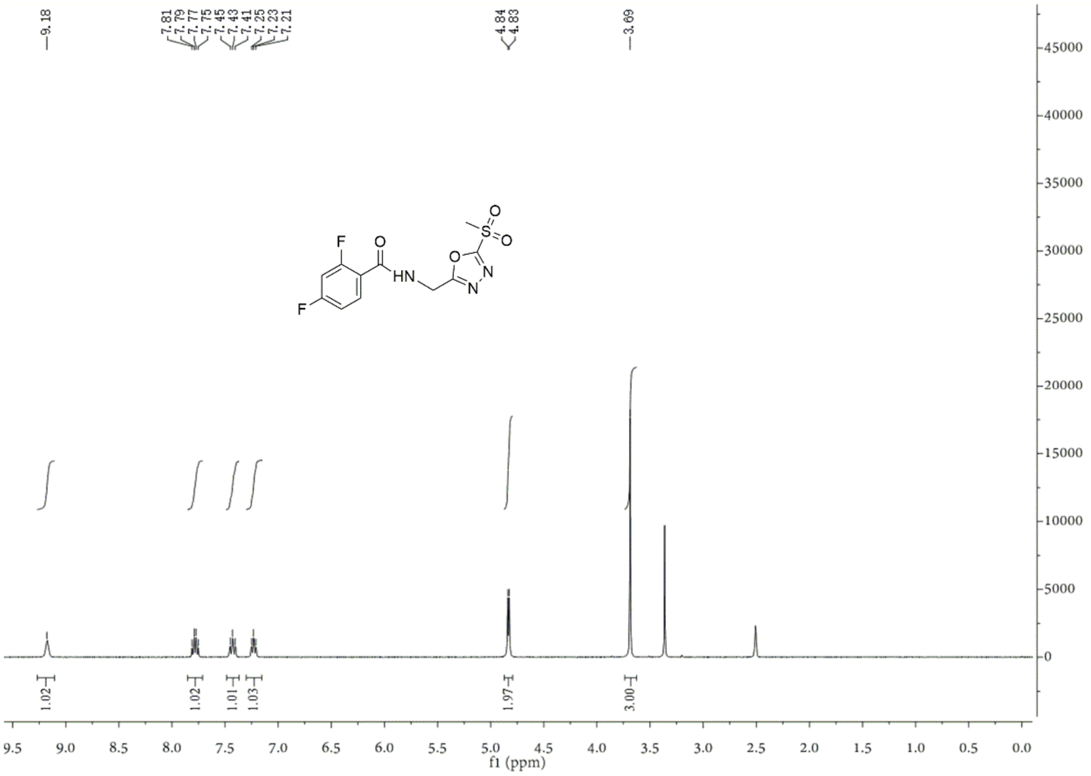

^{1}H NMR for compound **23**

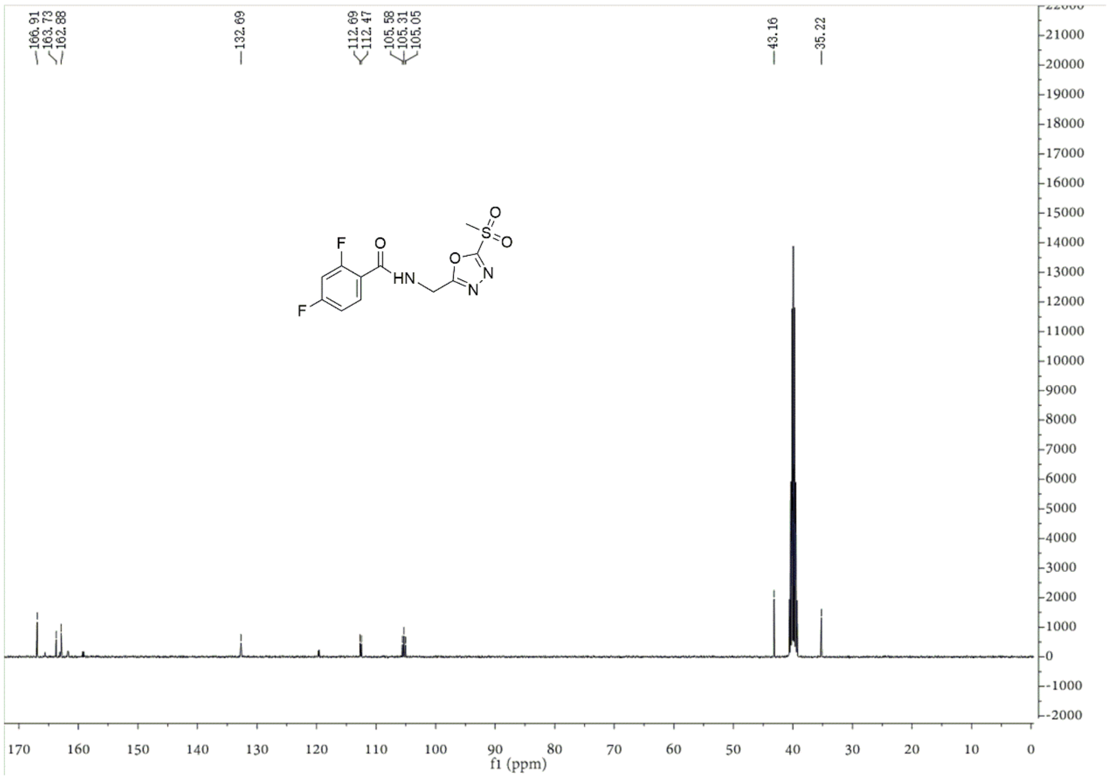

^{13}C NMR for compound **23**

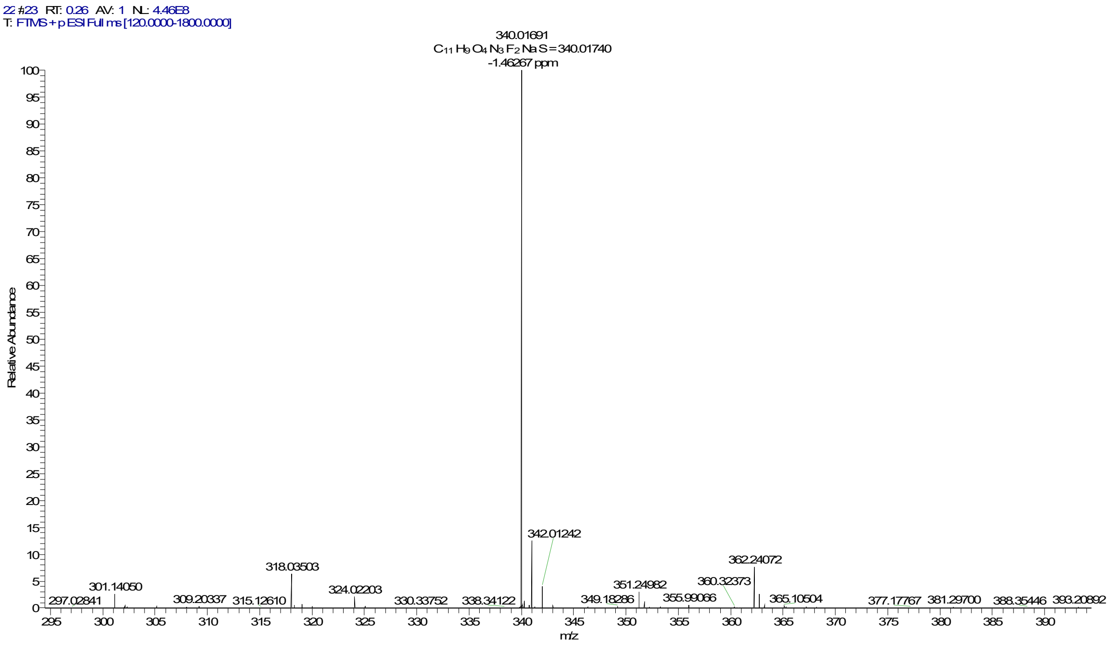

HRMS for compound **23**

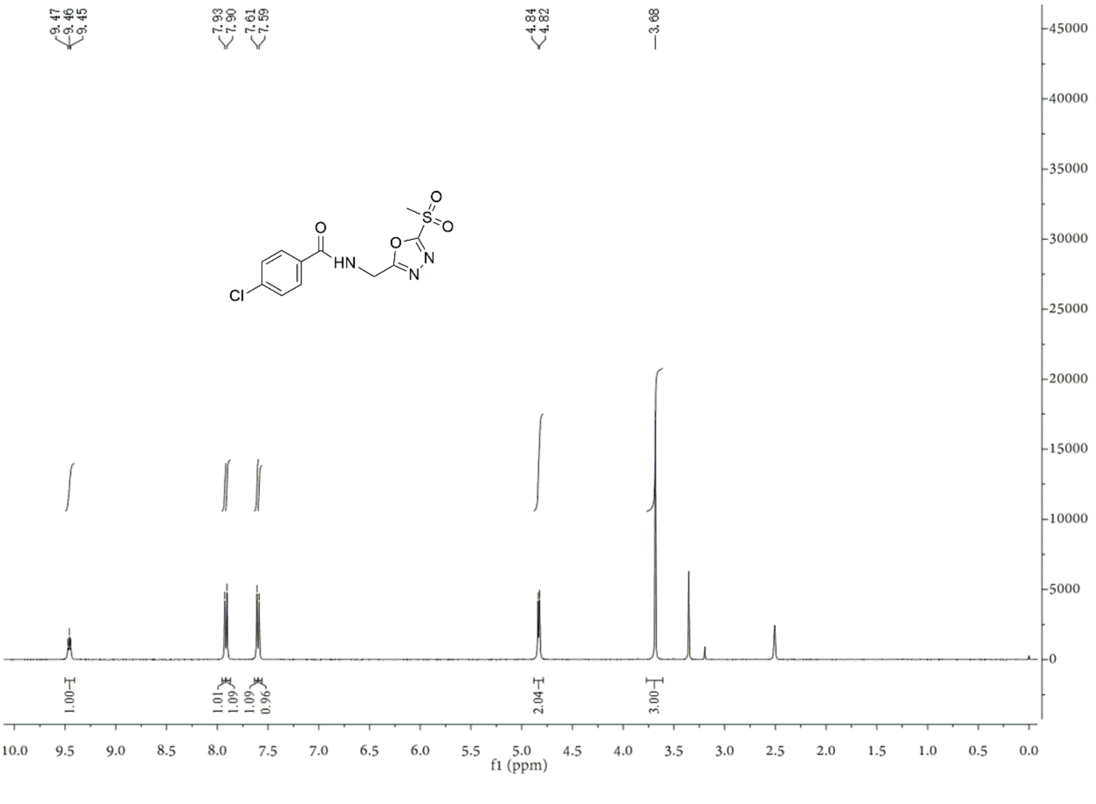

^{1}H NMR for compound **24**

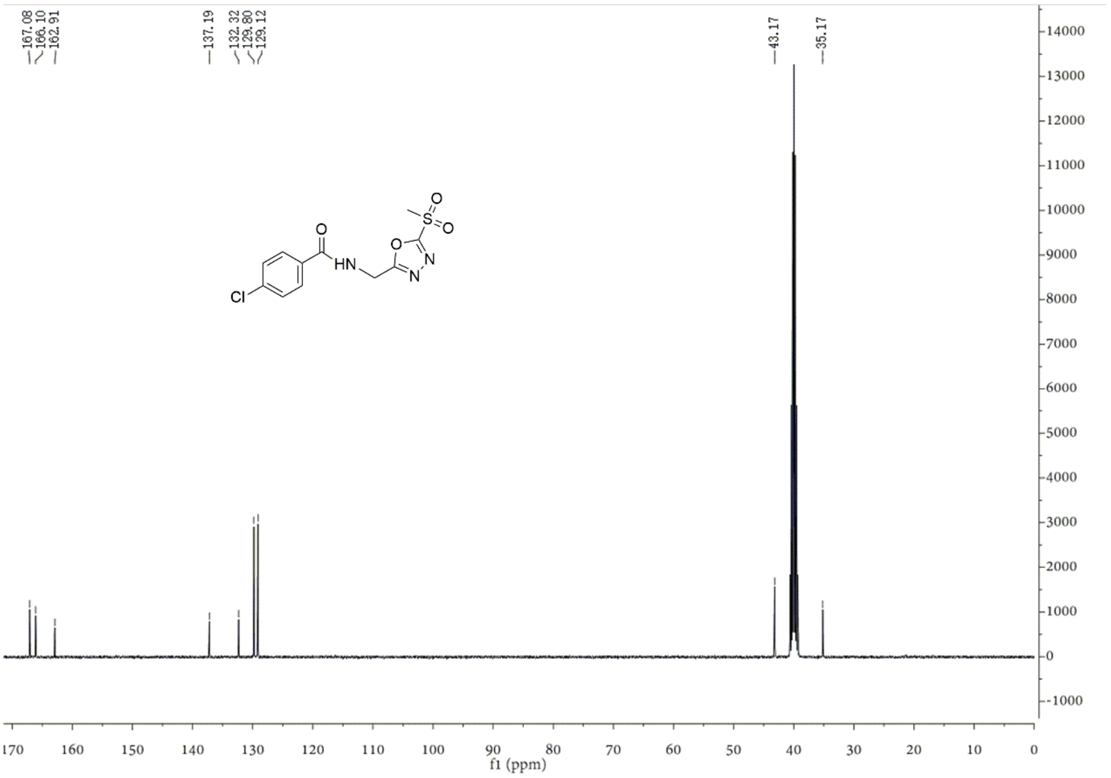

^{13}C NMR for compound **24**

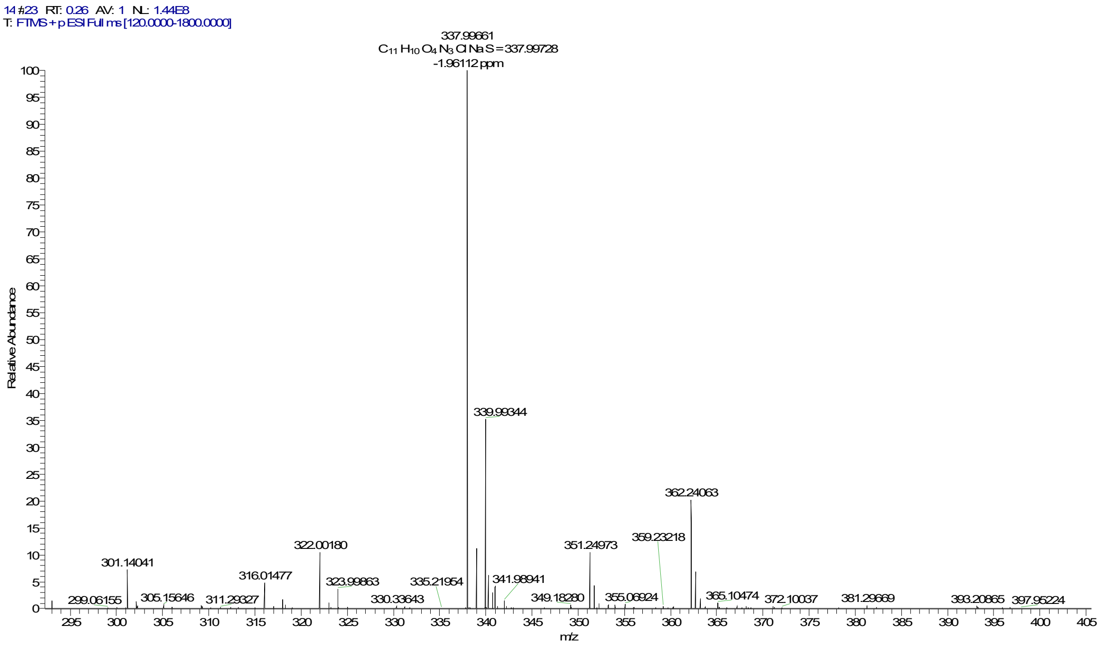

HRMS for compound **24**

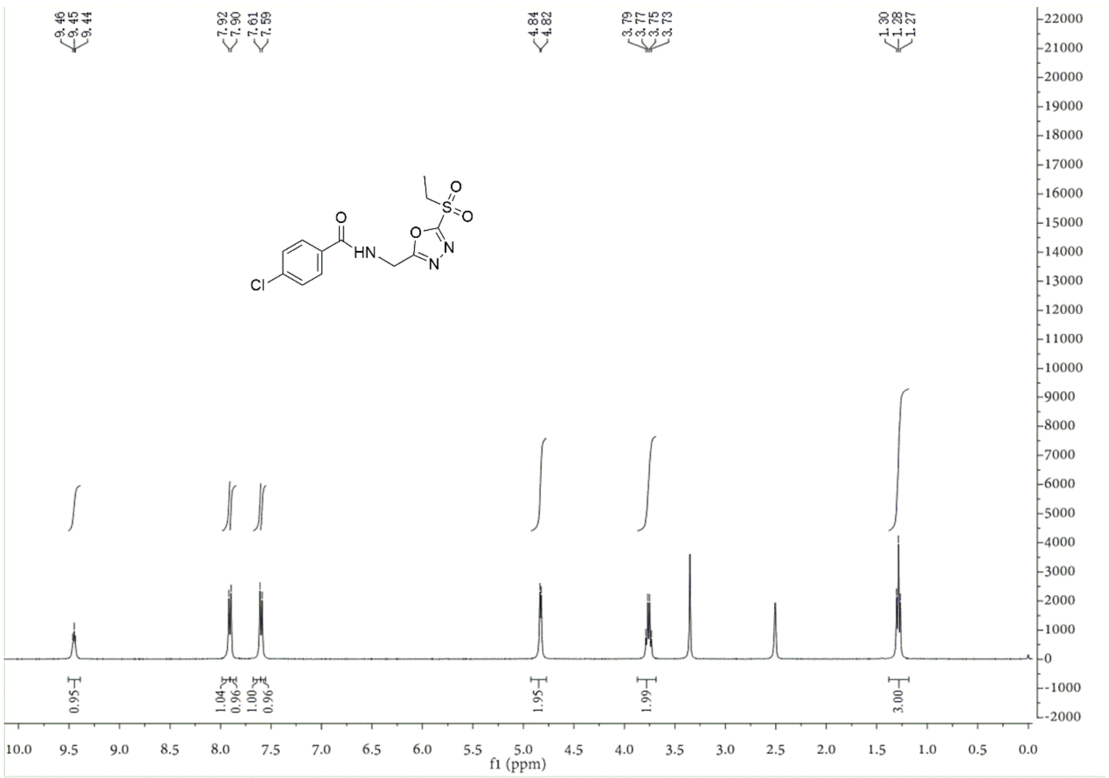

^{1}H NMR for compound **25**

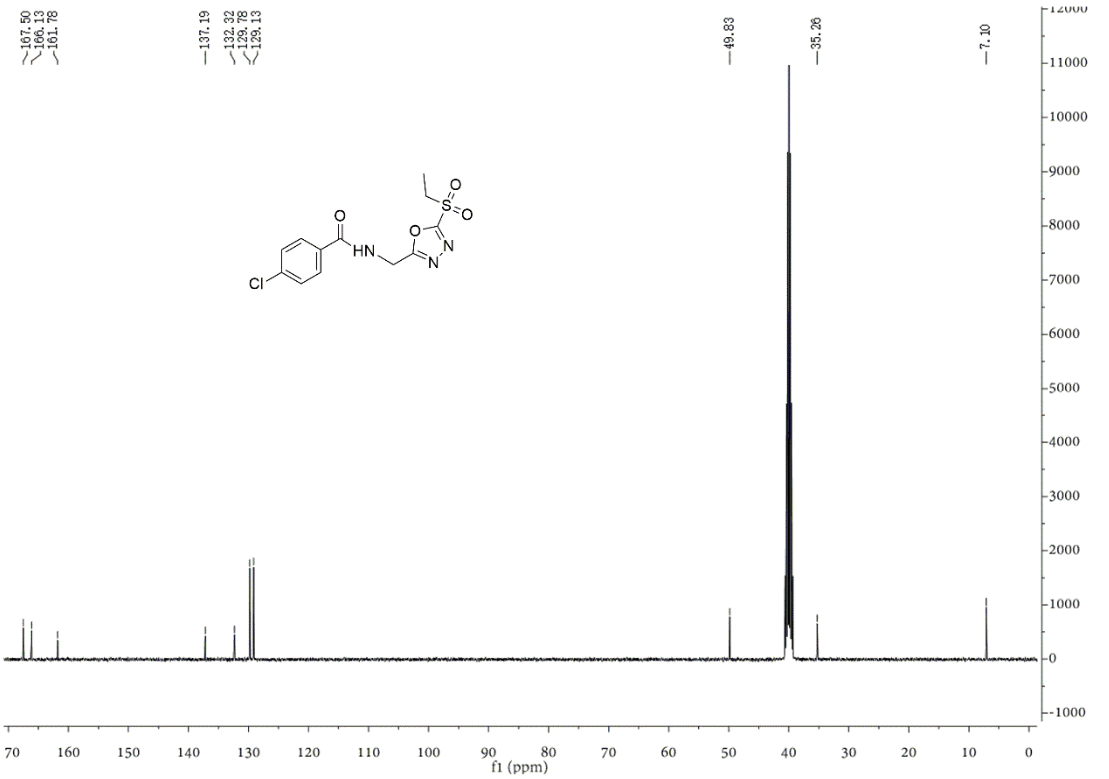

^{13}C NMR for compound **25**

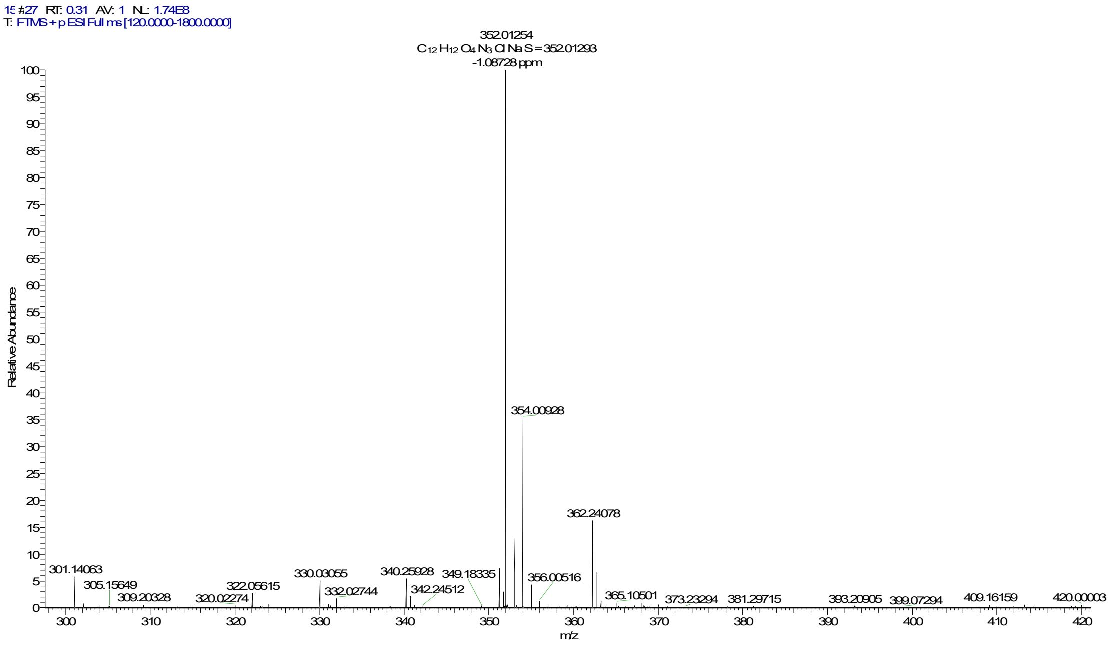

HRMS for compound **25**

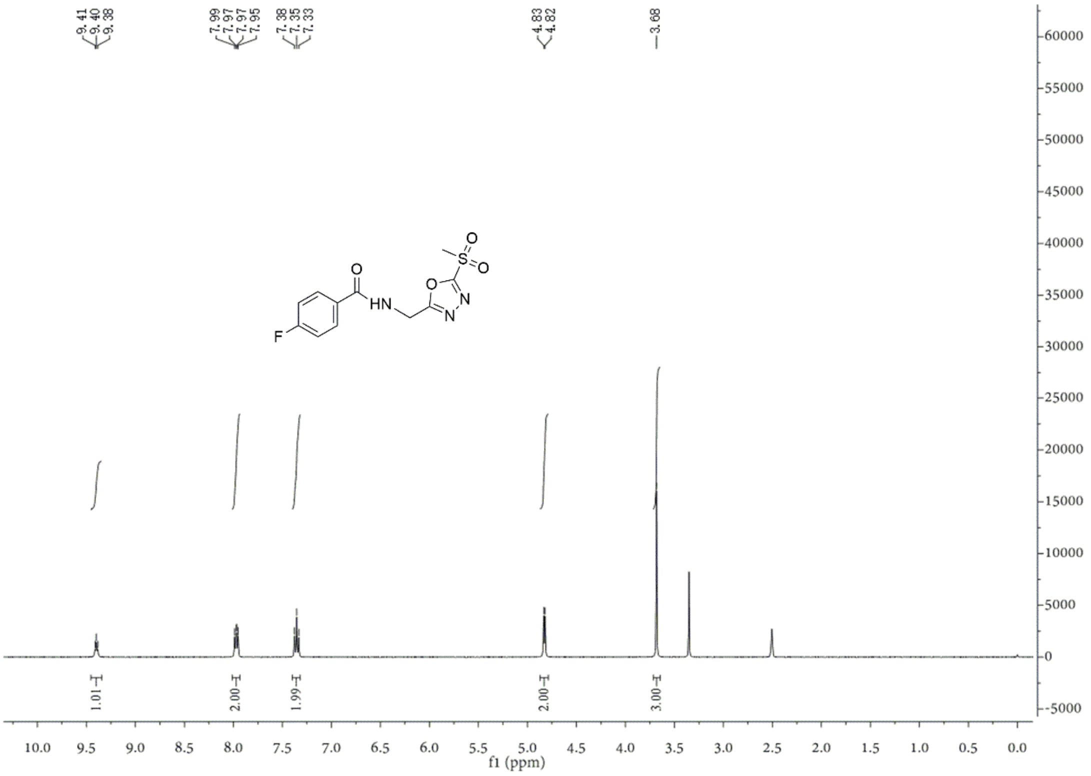

^{1}H NMR for compound **26**

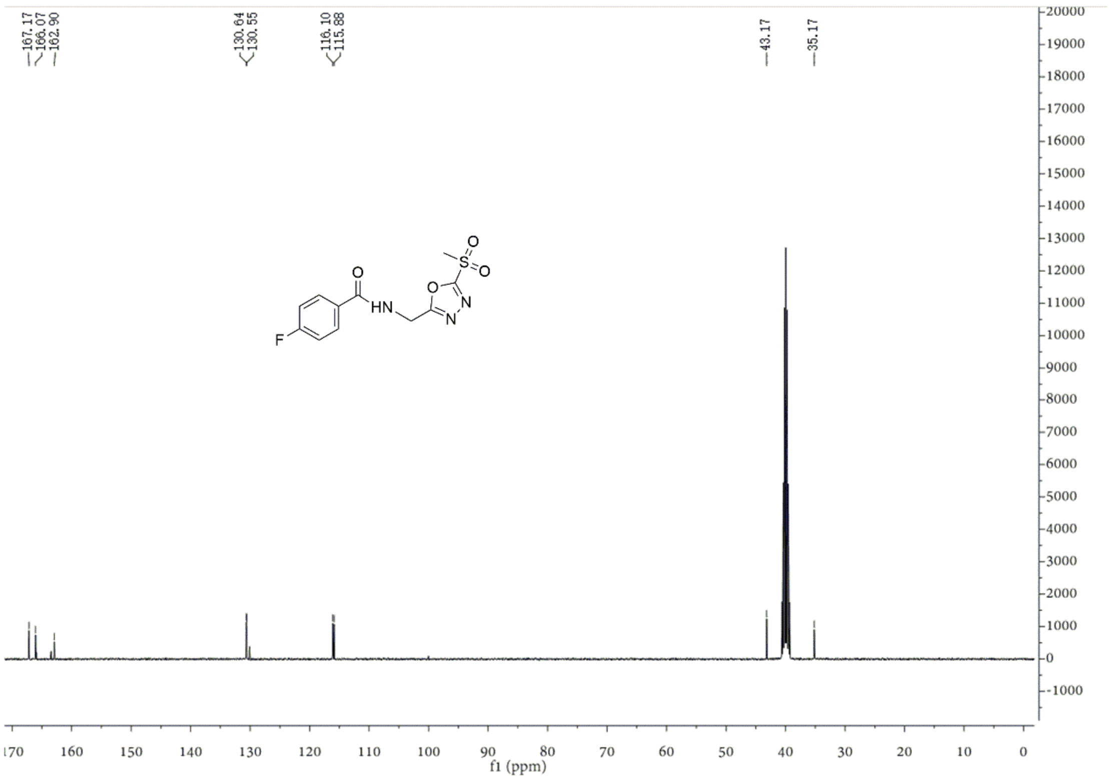

^{13}C NMR for compound **26**

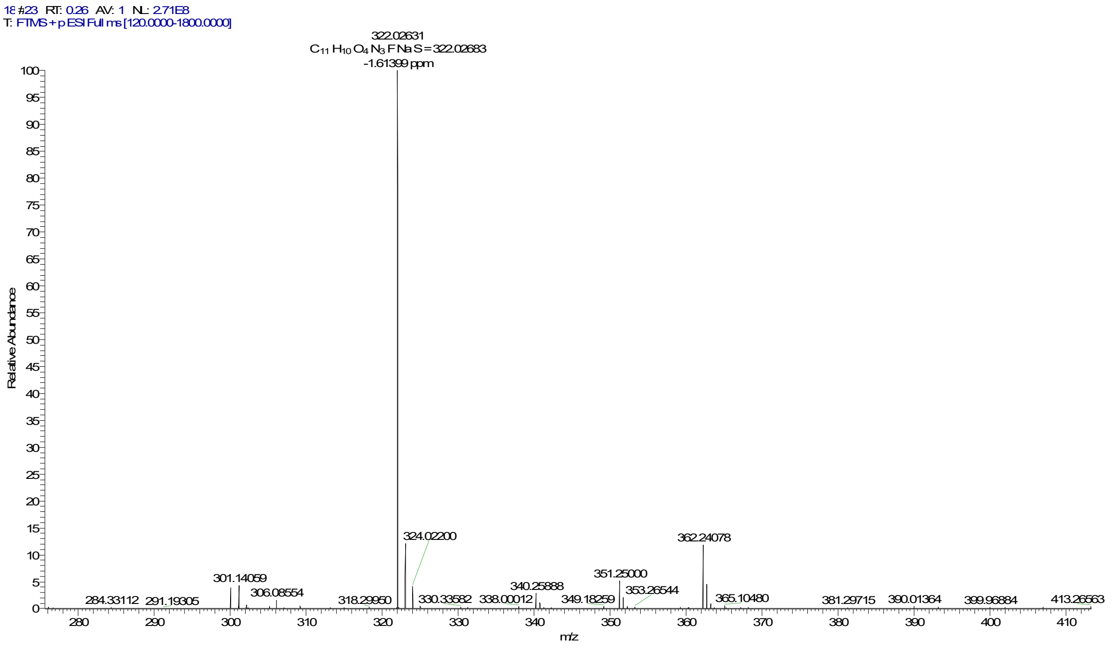

HRMS for compound **26**
